# A novel diagnostic approach for the classification of small B-cell lymphoid neoplasms based on the NanoString platform

**DOI:** 10.1038/s41379-021-00954-z

**Published:** 2021-11-20

**Authors:** Wei Zhang, Qilin Ao, Yuqi Guan, Zhoujie Zhu, Dong Kuang, Monica M. Q. Li, Kefeng Shen, Meilan Zhang, Jiachen Wang, Li Yang, Haodong Cai, Ying Wang, Ken H. Young, Jianfeng Zhou, Min Xiao

**Affiliations:** 1 Department of Hematology, Tongji Hospital, Tongji Medical College, Huazhong University of Science and Technology, Wuhan, Hubei Province 430000 P.R. China; 2 Institute of Pathology, School of Basic Medical Science, Tongji Medical College, Huazhong University of Science and Technology, Wuhan, 430000 P.R. China; 3 Department of Pathology, Tongji Hospital, Tongji Medical College, Huazhong University of Science and Technology, Wuhan, 430000 P.R. China; 4 Perfectgen Diagnostics, Ezhou, Hubei Province 436032 P.R. China; 5 Department of Computer Science, City University of Hong Kong, Kowloon, 999077 Hong Kong; 6 Division of Hematopathology, Duke University Medical Center and Cancer Institute, Durham, NC 27710 USA

**Keywords:** Reporter genes, Genetic testing

## Abstract

Small B-cell lymphoid neoplasms (SBCLNs) are a heterogeneous group of diseases characterized by malignant clonal proliferation of mature B-cells. However, the classification of SBCLNs remains a challenge, especially in cases where histopathological analysis is unavailable or those with atypical laboratory findings or equivocal pathologic data. In this study, gene expression profiling of 1039 samples from 27 gene expression omnibus (GEO) datasets was first investigated to select highly and differentially expressed genes among SBCLNs. Samples from 57 SBCLN cases and 102 nonmalignant control samples were used to train a classifier using the NanoString platform. The classifier was built by employing a cascade binary classification method based on the random forest algorithm with 35 refined gene signatures. Cases were successively classified as chronic lymphocytic leukemia/small lymphocytic lymphoma, conventional mantle cell lymphoma, follicular lymphoma, leukemic non-nodal mantle cell lymphoma, marginal zone lymphoma, lymphoplasmacytic lymphoma/Waldenström’s macroglobulinemia, and other undetermined. The classifier algorithm was then validated using an independent cohort of 197 patients with SBCLNs. Under the distribution of our validation cohort, the overall sensitivity and specificity of proposed algorithm model were >95%, respectively, for all the cases with tumor cell content greater than 0.72. Combined with additional genetic aberrations including *IGH-BCL2* translocation, *MYD88* L265P mutation, and *BRAF* V600E mutation, the optimal sensitivity and specificity were respectively found at 0.88 and 0.98. In conclusion, the established algorithm demonstrated to be an effective and valuable ancillary diagnostic approach for the sub-classification and pathologic investigation of SBCLN in daily practice.

## Introduction

Small B-cell lymphoid neoplasms (SBCLNs) are a group of diseases characterized by malignant clonal proliferation of mature B-cells, mainly including chronic lymphocytic leukemia/small lymphocytic lymphoma (CLL/SLL), follicular lymphoma (FL), mantle cell lymphoma (MCL), nodal marginal zone lymphoma (NMZL), splenic marginal zone lymphoma (SMZL), extranodal marginal zone lymphoma of mucosa-associated lymphoid tissue (MALTL), lymphoplasmacytic lymphoma/Waldenström’s macroglobulinemia (LPL/WM), and hairy cell leukemia (HCL)^1^. Because of the heterogeneity and complexity, routine diagnosis of SBCLN is still challenging, which usually requires experienced pathological examination and secondary review.

Researchers have revealed that the pathogenesis of some entities is characterized by distinct chromosomal aberrations, somatic mutations, or gene expression signatures, for instance, *IGH-BCL2* in FL, *IGH-CCND1* in MCL, +12 in CLL/SLL, *BIRC3-MALT1* fusion in MALTL, *MYD88* L265P in LPL/WM, *BRAF* V600E in HCL, and SOX11 overexpression in conventional MCL (cMCL)^2–7^. Thus, the diagnosis of SBCLN usually not only depends on pathological examination but also is completed by immunophenotypic, cytogenetic, and genomic methods, especially in cases that mainly present with leukemic involvement. Nonetheless, even with integrated diagnostic approaches, precise diagnosis of SBCLNs remains difficult in some cases with atypical laboratory findings.

Previous studies have demonstrated that the classification of lymphoid neoplasms based on gene expression signatures was feasible and accurate^8–11^. To date, technologies have been developed to reliably quantify low-throughput gene expression in RNA from either fresh/frozen or formalin-fixed paraffin-embedded (FFPE) tissue, allowing the development of clinically relevant RNA assays^9–14^. However, most of these assays focused on binary classification problems and only address a small proportion of lymphoid neoplasms, limiting their application in clinical practice. Here, by taking advantage of the NanoString platform, we developed a 35-gene expression-based classifier used for the classification of 6 main SBCLN entities. Initial candidate gene markers were selected from global gene expression profiling (GEP) analyses. Then, the classifier was trained by 57 SBCLN and 102 nonmalignant control cases, and further validated by an independent cohort of 197 SBCLN cases. In conclusion, we demonstrated a robust, highly accurate, and validated assay for SBCLN distinction using the NanoString platform.

## Materials and methods

### Patients and samples

In this study, total 159 cases were retrospectively enrolled in the training cohort, and 197 SBCLN cases were independently recruited in the validation cohort in our center (Tongji Hospital, Wuhan, China). Among the training cohort, 57 subjects were diagnosed as SBCLN, including 7 CLL/SLL, 13 FL, 9 cMCL, 4 leukemic non-nodal MCL (nnMCL), 19 MZL (NMZL, MALT, or SMZL), 5 LPL/WM cases, and 102 non-malignant biopsies from the sites where SBCLNs frequently developed (Table 1) (Supplementary Tables S1–4). For SBCLN cases, the training cohort included 8 fresh samples and 49 formalin-fixed paraffin-embedded (FFPE) samples, and the validation cohort consisted of 32 fresh samples and 165 FFPE samples. All diagnoses were established by at least three experienced hematopathologists according to the 2016 World Health Classification criteria. Tumor cell content was determined by the flow cytometric analysis in matched fresh samples, which was defined as the ratio of the number of tumor cells to the number of total nucleated cells. For samples with FFPE tissues only, tumor cell content was determined by at least three experienced pathologists (Supplementary Methods). Tumor cell content was required no less than 0.9 in each sample of the training cohort and was at least 0.3 in each sample of the validation cohort. The study was conducted in strict accordance with the guidelines formulated by the Tongji Hospital Ethics Committee (IRB ID: TJ-S1203), Wuhan, China. Written informed consent was obtained from each recruited subject in strict accordance with the Declaration of Helsinki.

**Table 1:** Patient demographic data and disease characteristics of training and testing cohort.

	Training cohort (*n* = 57)	Validation cohort (*n* = 197)
Age of onset (years)		
Median (range)	53 (22~81)	58 (13~85)
Gender		
Male	31	118
Female	26	79
Subtype		
CLL/SLL	7	14
cMCL	9	27
nnMCL	4	3
FL	13	65
MZL	19	67
LPL/WM	5	17
Other SBCLNs	0	4
Sample type		
FFPE tissue	49	166
Fresh/Frozen sample	8	31
Location		
Lymph node/Waldeyer’s ring	35	101
Peripheral blood/bone marrow	0	8
Extranodal tissue	14	65
Other	8	23
Tumor cell content		
>0.9	57	11
0.7~0.9	0	84
0.3~0.7	0	102

*CLL/SLL* chronic lymphocytic leukemia/small lymphocytic lymphoma, *cMCL* conventional mantle cell lymphoma, *nnMCL* leukemic non-nodal mantle cell lymphoma, *FL* follicular lymphoma, *MZL* marginal zone lymphoma, *LPL/WM* lymphoplasmacytic lymphoma/Waldenström’s macroglobulinemia, *FFPE* formalin-fixed paraffin-embedded.

Genomic DNA and RNA were extracted with AllPrep FFPE DNA/RNA Kit (Qiagen, Germany) for FFPE samples or AllPrep DNA/RNA Mini Kit (Qiagen, Germany) for fresh/frozen samples by following the manufacturer’s instructions. The concentrations of genomic DNA and RNA were quantitatively determined on a Qubit 3.0 fluorometer (Thermo Fisher Scientific, USA).

### Immunophenotypic, cytogenetic, and mutational analyses

Immunophenotype, several distinct chromosomal aberrations and somatic mutations were detected and available in all SBCLN cases of the training and validation cohorts at diagnosis. Immunohistochemical (IHC) staining of FFPE tissues and flow cytometry of fresh samples were performed for CD5, CD10 (*MME*), Cyclin D1 (*CCND1*), SOX11, CD38 and other additional biomarkers according to the manufacturer’s protocols. The fluorescence in situ hybridization (FISH) study performed on FFPE tissue sections or fixed cells from cytogenetic cultures was used to interrogate breaks of the loci *BCL2*, *CCND1*, *MALT1*, and *IGH* (dual color, break apart rearrangement probes) and identify chromosomal abnormalities, including +12 and del(7q), using commercially available probes (Abbott Molecular, USA). Sanger Sequencing was conducted for determining at least 5 hotspot mutations including *EZH2* exon18 (Y646), *MYD88* exon5 (L265P), *BRAF* exon15 (V600E), *CXCR4* exon2 (S338X), and *NOTCH2* exon34 (R2400X). (Supplementary Table S5). A next-generation sequencing (NGS) panel targeted 157 lymphoma-associated genes was also designed to identify other mutations in several samples (Designstudio Sequencing, Illumina, USA) (Supplementary Table S6). The sequencing library was prepared with 20 ng of input DNA per sample and sequenced to 1000× coverage. Generated variants were annotated using Annovar^15^. Exonic nonsynonymous or splice donor/acceptor site variants with reads ≥20 were initially filtered. Then, variants with a population frequency > 0.0001 in the gnomAD database (gnomAD.broadinstitute.org) were excluded unless they were relevant to lymphoma according to the COSMIC database (https://cancer.sanger.ac.uk/cosmic/).

### Quantitative gene expression study

Digital gene expression of samples in the training and validation cohorts was performed with 200 ng of RNA input on the NanoString platform (NanoString Technologies, USA), using the “high sensitivity” setting on the nCounter™ PrepStation and 550 field of view (FOV) on the nCounter™ Analyzer. Normalization of digital gene expression was performed using the geometric mean of the counts of appropriate housekeeping genes for the training cohort and the validation cohort separately. The normalized data were then log_2_ transformed for further analyses.

### Statistical analysis

All statistical analyses were conducted by employing R v3.6.2. Differences were evaluated by using Fisher’s exact test for categorical variables. The significance of the co-occurrence or mutual exclusivity was also assessed by utilizing Fisher’s exact test. Nonpaired *t* tests were used to compare the gene expression level between groups, as appropriate. Unless otherwise specified, a two-tailed *P* < 0.05 was considered statistically significant for all analyses.

## Results

### Selecting initial candidate genes for classification

**Fig. 1:**
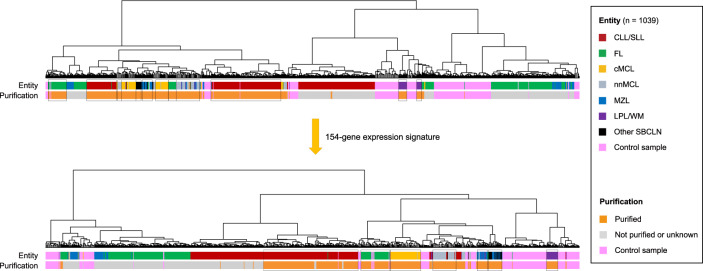
Unsupervised hierarchical clustering of expression profiling of 1039 SBCLN and control cases. A tree diagram based on the whole gene expression signature is shown above. A tree diagram based on the targeted 154-gene expression signature is shown on the bottom. Each black box highlights a major distribution branch of a reliable SBCLN entity.

In a first step towards developing a classification model, the global GEP of 1039 samples from 27 Gene Expression Omnibus (GEO) datasets (Affymetrix U133 plus 2.0 microarrays, Thermo Fisher Scientific, USA) was initially investigated, including 808 SBCLNs and 231 nonmalignant control cases (Supplementary Tables S7-S8) (https://www.ncbi.nlm.nih.gov/geo/query/acc.cgi). Raw data of all the samples were normalized together using the 3′ robust multiarray average (3′ RMA) and analyzed by using one-way between-subject analysis of variance (ANOVA). For each subgroup, differentially expressed genes were selected by one-vs-rest or one-vs-one strategies (Supplementary Table S9). Then, 136 genes were revealed as differentially expressed genes with absolute log_2_-fold change > 1 and a significant FDR (<0.05). Moreover, 18 other differentially expressed genes referring to associated studies were also included as an essential supplement^10,13,16^ (Supplementary Table S9). Based on the geNorm algorithm, 13 genes with stable expression were selected as potential housekeeping genes^17^. Subsequently, these genes were validated in the GEP of 1039 samples using an unsupervised hierarchical clustering approach. Clustering results demonstrated that samples were dominantly distributed in terms of their respective entity and status of sample purification. Compared with the whole gene expression signature, the targeted 154-gene expression signature performed better in clustering samples, because most subgroups had fewer cluster branches and most cluster branches gathered more samples of the same subgroup (Fig. 1) (Supplementary Fig. S1) (Supplementary Table S8). The clustering results indicated that our 154-gene expression signature was feasible for the classification of SBCLN. Thus, a NanoString codeset panel consisting of 154 candidate genes for the classification of SBCLN, along with 13 potential housekeeping genes, was designed for quantitating gene signatures (NanoString Technologies, USA) (Supplementary Table S9). In this panel, capture probes in the codeset were designed to target conserved sequences within all transcripts of each gene.

### Quantification of targeted gene expression based on the NanoString platform

**Fig. 2:**
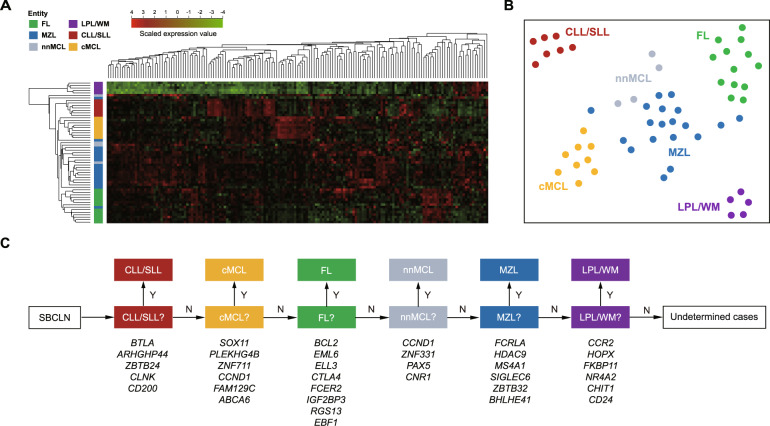
Developing a molecular classifier of SBCLN based on the NanoString Platform. **A** Hierarchical clustering heatmap based on targeted genes of 57 SBCLN cases in the training cohort. Genes are shown in rows, and cases are shown in columns in the heatmap (red: high expression, green: low expression). Each SBCLN entity is represented by a different color. **B** Digital gene expression data of 57 SBCLN cases shown in a two-dimensional tSNE plot. Targeted genes were selected and processed by the tSNE algorithm with a perplexity score of 30. Major SBCLN entities are highlighted in different colors. **C** Schematic representation of the predictor model. In the lower part, the genes used for the classification in each step are indicated.

Targeted gene expression was thereby quantified in 57 SBCLN and 102 control cases of the training cohort. At the beginning of data processing, 4 genes (*ACTB*, *GAPDH*, *GUK1*, and *GUSB*) were selected as the final subset of housekeeping genes because the other 9 potential housekeeping genes had low expression levels or a high coefficient of variation across the samples. First, the normalized 154-gene signature of 57 SBCLN cases was analyzed by an unsupervised hierarchical clustering approach. Cases were dominantly clustered into 5 main branches according to their respective subgroup: one was mostly made up of FL cases (13/14, 93%); one included the majority of MZL (16/19, 84.2%) but mingled with 3 nnMCL cases; one was mainly composed of cMCL cases (9/10, 90%); another consisted of all CLL/SLL cases (7/7, 100%); and the last one was exclusively comprised of LPL cases (5/5, 100%) (Fig. 2A). Overall, ~87.7% (50/57) of cases were appropriately clustered, suggesting the feasibility of subsequent development of a classification model using the NanoString platform.

### Development of SBCLN classification model

**Fig. 3:**
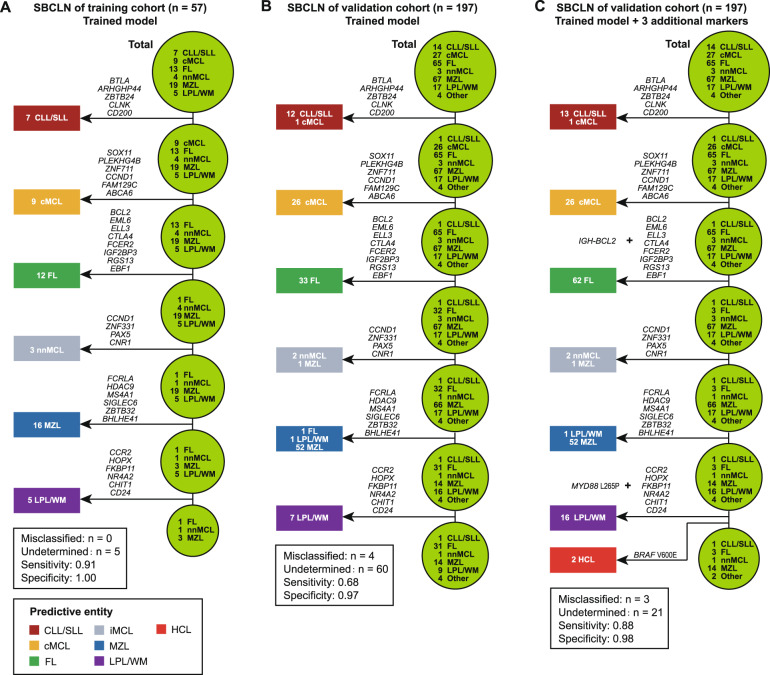
Performance of the model in each cohort. **A** Self validation of the model in the training cohort using the LOO cross-validation strategy. **B** Performance of the model in the validation cohort. **C** Performance of the model combined with 3 additional markers in the validation cohort. HCL cases could be additionally identified by *BRAF* V600E.

To overcome the difficulty of multiclassification, the model was decomposed into a cascade of one-vs-rest binary classifiers used for multistep discriminations, which was similar to that described by Navarro et al.^16^. Each classifier was trained based on the random forest algorithm using the scikit-learn library for the Python programming language (Python Software Foundation, https://www.python.org/) with standard parameters. The order of an SBCLN entity to discriminate was decided according to Davies-Bouldin Index (DBI). Entities with lower DBI were more distinguishable from the other entities and were given priority to discriminate (Supplementary Table S10) (Fig. 2B). In each step, the classifier will determine if a given sample belongs to a designated SBCLN subgroup. Cases were classified as the respective SBCLN if they had a ≥0.5 prediction probability of belonging to the designated entity. The leave-one-out (LOO) cross-validation strategy was used to evaluate the sensitivity and specificity of each classifier. For each classifier, the importance Gini index was used to evaluate the classification ability of all 154 candidate genes, and only the top valuable upregulated genes in discriminating an entity were selected as the final subset of markers to build the one-vs-rest classifier of the entity (Supplementary Table S11). Details of the modeling method are described in the Supplementary Methods. Finally, with a set of 35 genes for the classification in total (Table 2) (Supplementary Fig. S2), our model was trained to successively determine whether a sample belongs to CLL/SLL, cMCL, FL, nnMCL, MZL, LPL/WM, or ultimately annotated as “undetermined cases” (Fig. 2C). CLL/SLL was the first entity to discriminate in the model, with 5 differentially upregulated genes (*BTLA*, *ARHGAP44*, *ZBTB24*, *CLNK*, and *CD200*), and was followed by cMCL with 6 markers, FL with 8 markers, nnMCL with 4 markers, MZL with 6 markers, and LPL/WM with 6 markers. Among them, only *CCND1* was used two or more times for prediction (both cMCL and nnMCL). The entire model was internally evaluated with the LOO cross-validation strategy. Focusing on SBCLN cases, the overall predictive accuracy was 91.2% (52/57) (Fig. 3A). Five SBCLN cases not classified into their belonging entities, including 1 FL, 1 nnMCL, and 3 MZL cases, were all predicted as “undetermined cases”. This result demonstrated a 100% specificity of our model since no case was misclassified (Supplementary Table S12).

**Table 2:** Genes that finally selected in building the molecular classifier.

Classification	Differentially expressed genes
CLL/SLL	*BTLA, ARHGAP44, ZBTB24, CLNK, CD200*
cMCL	*SOX11, PLEKHG4B, ZNF711, CCND1*^*a*^*, FAM129C, ABCA6*
FL	*BCL2, EML6, ELL3, CTLA4, FCER2, IGF2BP3, RGS13, EBF1*
nnMCL	*CCND1*^*a*^*, ZNF331, PAX5, CNR1*
MZL	*FCRLA, HDAC9, MS4A1, SIGLEC6, ZBTB32, BHLHE41*
LPL/WM	*CCR2, HOPX, FKBP11, ANK3, ZNF226, MFAP5, MEF2A*
Additional markers	
FL	*IGH-BCL2* translocation or t(14;18)(q32;q21)
LPL/WM	*MYD88* L265P mutation
HCL	*BRAF* V600E mutation

*CLL/SLL* chronic lymphocytic leukemia/small lymphocytic lymphoma, *cMCL* conventional mantle cell lymphoma, *nnMCL* leukemic non-nodal mantle cell lymphoma, *FL* follicular lymphoma, *MZL* marginal zone lymphoma, *LPL/WM* lymphoplasmacytic lymphoma/Waldenström’s macroglobulinemia, *HCL* hairy-cell leukemia.

^a^*CCND1* gene was selected as marker in both cMCL and nnMCL.

### Performance of the model in validation cohort

**Fig. 4:**
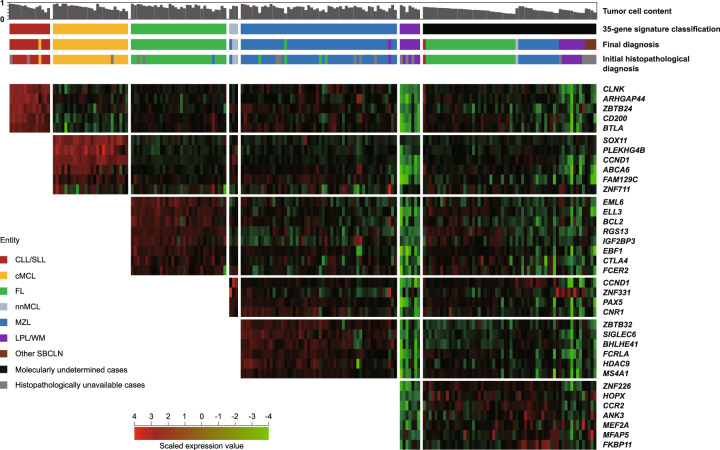
The molecular classifier based on machine learning. A multistep molecular discrimination of 197 SBCLN cases in the validation cohort is shown in a heatmap based on digital expression data. Each column represents an SBCLN case, and each row represents a variable gene in the heatmap (red: high expression, green: low expression; scaled by *z* statistics). In each row, the gene expression of the remaining cases was scaled by *z* statistics. All selected genes were included in the refined subset and clustered according to their corresponding entities. Tumor cell content, pathological diagnosis, predictive subgroup by molecular classifier, and integrated diagnosis are indicated above.

To determine the validity and reproducibility of the model in an extended and independent dataset, we constructed a validation cohort consisting of 197 SBCLN samples. The tumor cell content ranged from 0.32 to 0.94 (mean 0.67). Digital gene expression data of the validation cohort were independently normalized using 4 final housekeeping genes. Using the 35-gene signature model, 137 cases in total were identified as one of the 6 SBCLN entities, and the other 60 cases were ultimately undetermined (Fig. 4). Among those 137 cases, initial pathological diagnosis was incorrect in 1 FL and 3 MZL cases, and histopathological examination was unavailable in the other 13 cases (Supplementary Table S4), but these 17 cases were all accurately classified into their respective entities by our model. However, misclassification of our model was found in 1 cMCL, 1 FL, 1MZL, and 1 LPL/WM. Overall, the sensitivity and specificity of our model were 0.68 (133/197) and 0.97 (133/137), respectively (Fig. 3B). The fixation method did not affect the predictive accuracy of model since the differences of predictive accuracy between fresh samples and FFPE samples was not significant (Supplementary Fig. S3).

**Fig. 5:**
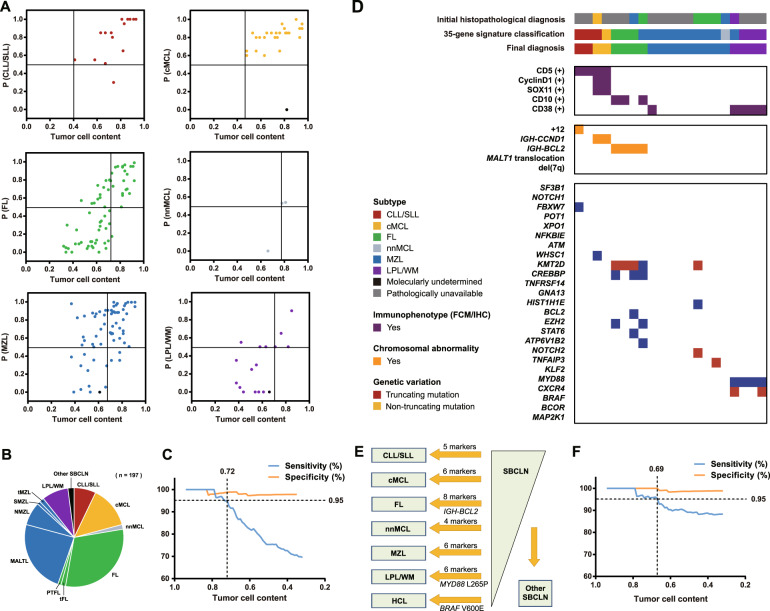
Performance of the model in validation cohort. **A** Scatter plot demonstrating the correlation between the tumor cell content of the sample and the probability of the case belonging to the respective entity. Colored plots indicated correctively classified cases. Black circles represented misclassified cases. **B** Fan chart showing the distribution of cases in the validation cohort. PTFL, pediatric-type FL; tMZL, transformed MZL. **C** Cumulative sensitivity and specificity of the model. The overall sensitivity and specificity of our model were both >95% empirically within cases with tumor cell content no less than 0.72 in our validation cohort. **D** Immunophenotypic and genetic features of 25 cases studied by NGS. Features are shown as rows, and samples are shown as columns. Immunophenotypic analysis was performed by flow cytometry. Cytogenetic studies were examined by FISH study. Genetic variations were generated by targeted NGS. The initial histopathological diagnosis, molecular entity predicted by the model, and final diagnosis are indicated above. **E** Modification of the model with *IGH-BCL2* translocation, *MYD88* L265P mutation, and *BRAF* V600E mutation added. **F** Cumulative sensitivity and specificity of the modified model. The overall sensitivity and specificity of our model were both >95% empirically within cases with tumor cell content no less than 0.69 in our validation cohort.

As expected, the mean tumor cell content in ultimately undetermined cases was significantly lower than that in determined cases (0.55 vs 0.72, *p* < 0.05), which indicated that tumor cell content had a great impact on the predictive probability of a case belonging to the respective entity. To make >90% of cases with a tumor cell content no less than the cutoff value have a >0.5 probability of belonging to their respective SBCLN entity, the minimal cutoffs of the tumor cell content were determined to be 0.41 in CLL/SLL, 0.48 in cMCL, 0.72 in FL, 0.78 in nnMCL, 0.68 in MZL, and 0.71 in LPL/WM (Fig. 5A). These results also indicated that our model is more tolerant to lower tumor cell contents in CLL and cMCL samples than in other SBCLN samples. Under the distribution of our validation cohort (Fig. 5B), the overall sensitivity and specificity of our model were both >95% within cases with tumor cell content ≥0.72 (Fig. 5C) (Supplementary Table S13).

We further studied whether combining with other specific chromosomal aberrations and recurrent mutations used in the diagnosis could improve the sensitivity or specificity of our model (Supplementary Table S4). Among 137 cases identified as one of the 6 frequent SBCLN entities, NGS was additionally performed in 4 pathologically misdiagnosed cases, 4 molecular misclassified cases, and 17 other cases with unavailable pathological examination (Fig. 5D). Additional genetic events were considered a combined marker of the model only if it could accurately reclassify ≥2 cases. Combined with *EZH2* Y646, *MYD88* L265P, and *BRAF* V600E mutations, the overall sensitivity and specificity were 0.77 (151/197) and 0.98 (151/154), respectively. If only *IGH-BCL2* was included as a marker of FL, the overall sensitivity and specificity were 0.82 (162/197) and 0.98 (152/155), respectively. If *IGH-BCL2*, *MYD88* L265P, and *BRAF* V600E were included as supplemental markers of the model, HCL cases could be additionally identified, and the overall sensitivity and specificity could reach 0.88 (173/197) and 0.98 (173/176), respectively (Fig. 3C) (Fig. 5E). More than 95% of cases were correctly classified within cases with tumor cell content ≥0.69 (Fig. 5F).

## Discussion

The diagnosis of SBCLNs has been notoriously difficult, especially in cases that predominantly had lymphocytosis but did not have lymphadenopathy or accessible tissues for diagnosis. Previous studies have indicated that gene expression signatures could facilitate the diagnosis and classification of SBCLNs, but most of them were largely of binary nature. In this study, using the NanoString platform, we developed a 35-gene signature-based RNA assay to identify major subtypes of SBCLNs, which was validated by an independent cohort. The overall accuracy was >95% within highly purified cases and could be higher if *IGH-BCL2*, *MYD88* L265P, and *BRAF* V600E were included as supplemental markers. Since this approach requires only very limited laboratory handling and could reliably quantify the expression of a set of genes without interobserver variability, it exhibited potential clinical application prospects as an ancillary method of classification of SBCLN.

There were many difficulties in the development of the model. First, in consideration of the difficulty of an intralaboratory large-scale GEP or RNA-sequencing study, a total of 1039 available global GEP data was analyzed to select differentially expressed genes, and related reports were also referred to include supplemental gene markers. Thus, a panel with an expanded number of 154 candidate genes was designed, since the unsupervised hierarchical clustering result demonstrated that targeted gene expression signatures were basically capable of SBCLN classification. However, potential deviations could result from different submitters. Therefore, targeted gene expression was also reliably quantified under the same experimental conditions using the NanoString Platform, and the classification capability of each candidate gene was reevaluated. In the subsequent modeling process, a subset of classification markers was finally refined to an optimal size with 35 genes in total, which further increased the clinical applicability of the assay. Second, multiclassification has usually been much more difficult than binary classification. To cope with this problem, we split the multiclass classification model into a cascade of binary classifiers. The order of an SBCLN entity to discriminate and an optimal size of gene markers were determined according to popular machine-learning methods, which are detailed in the Supplementary Methods. Thus, each binary classifier was finally developed based on the random forest algorithm because one-dimensionalization in advance was not necessary, and this method performed best within multiple machine-learning algorithms, especially in the classification of entities difficult to distinguish from one another, including nnMCL, MZL, and LPL/WM^18^. Third, it was more challenging for the multiclassification model that samples for routine tests were usually inconvenient to purify. We did not include more tumor samples to serve the model better since it was difficult to collect highly purified samples with complete immunophenotypic, cytogenetic, and mutational results. Therefore, 102 nonmalignant biopsies from the sites where SBCLNs frequently developed were included as control samples to standardize the model. As expected, the inclusion of a large number of control samples reduced the sensitivity of the model, especially to samples with low tumor cell content; however, it increased the specificity of the model. It should be noted that the validity of our model was evaluated based on our given validation set, and the tumor cell content in each sample was ≥0.3. However, the predictive specificity of the model did not significantly decrease in samples with low tumor cell content (Fig. 5C). Thus, what limited the clinical applicability of the assay is that the predictive sensitivity decreased when the model was applied to samples with low tumor cell content. To address this problem, minimum tumor cell content was determined to be 0.72 for reliable classification, since each qualified sample has a ≥95% probability of being correctly classified. Another approach was, as one of the ancillary diagnostic methods, the accuracy of the classification could be improved if specific genetic or cytogenetic aberrations were also detected. The *IGH-BCL2* translocation is a hallmark of FL and is present in 80% FL cases and even 90% in low-grade FL cases, but rarely identified (3%) in non-FL SBCLN cases^19,20^. Similarly, *MYD88* L265P and *BRAF* V600E mutation was specifically detected in >90% LPL/WM and HCL cases, respectively. By contrast, both mutations were present in <5% other subtypes of SBCLN^3,16,21,22^. Therefore, if *IGH-BCL2* translocation, *MYD88* L265P mutation, and *BRAF* V600E mutation were also detected and the results were combined with the model, the overall predictive sensitivity could increase to 0.88, and was markedly improved in samples with low tumor cell content. However, the modified model still needs to be improved in the discrimination of the MZL cases with low tumor cell content.

We also further focused on our final subset of gene markers. Constitutive *CCND1* and *BCL2* overexpression, which is due to chromatin translocation, is a well-known hallmark of MCL and FL, respectively. *BTLA* is an immune checkpoint suppressor B lymphocyte attenuator, and overexpression of *BTLA* in CLL/SLL, which probably leads to immune escape and involved in the pathogenesis of CLL/SLL, has been described by several studies^23,24^. The neural transcription factor *SOX11* has emerged as a cooperative key oncogenic factor in the pathogenesis of cMCL, whereas it is not expressed in normal B cells or virtually in any other mature B-cell neoplasm^16,25^. *SOX11* is even also highly expressed in a minority of cMCL characterized by *CCND2*/*CCND3* rearrangements with *IGK*/*IGL* enhancers^26^. Therefore, it has also been instrumental to specifically distinguish cMCL from other SBCLNs. Previous research has illustrated that *IGF2BP3* (IMP-3) overexpression is seemingly restricted to several epithelial malignancies correlated with aggressive behavior and lymphomas originating from physiologic germinal center B cells. Hodgkin lymphoma, Burkitt lymphoma, FL, and diffuse large B-cell lymphoma all demonstrated IMP-3 positivity in >80% of cases^27^. Consistent with these findings, our data also showed that IMP-3 was a well-performing biomarker in distinguishing FL from other SBCLN cases. Moreover, some genes encoding clusters of differentiation antigens were also selected in the refined subset, including *MME* (CD10), *FCER2* (CD23), and *MS4A1* (CD20). However, most differentially expressed genes were not reported by previous studies, and further research is necessary since the association between gene overexpression and oncogenesis has not been elucidated.

There are some limitations of the model that need to be improved. First, more highly purified SBCLN cases should be included in further studies, especially cases of entities with limited sample sizes or those not included in our training cohort. Second, a larger panel of genes need to be further evaluated if the challenge lies in discriminating samples with low tumor cell content because it is usually inconvenient to enrich tumor cells in routine practice, especially for samples with only FFPE tissue available. Third, more cases with atypical/inconclusive genetic findings or belong to rare subtypes should also be included in the validation cohort, such as CLL carrying the *IGH-BCL2* translocation, FL without the *IGH-BCL2* translocation, Cyclin D1-negative cMCL, or SBCLN, not otherwise specified (NOS) cases. Regarding experimental methodology, consistency among different tissue types, serial dilution experiments, and intra- and interlaboratory reproducibility of the model should be determined. These works can improve the accuracy and extend the clinical applicability of the model.

In conclusion, we described a feasible model based on a digital gene expression platform that can classify SBCLNs independent of sample type with a good performance. Despite some limitations, our work provides a novel alternative for the routine diagnosis and subclassification of SBCLNs.

## Supplementary information

Supplementary Appendix

## Data Availability

The NanoString profiling of the training cohort and the validation cohort has been deposited in the Gene Expression Omnibus (GEO) and is accessible through GEO Series accession number GSE183030.
