# Supplementary material for: A novel diagnostic approach for the classification of small B-cell lymphoid neoplasms based on the NanoString platform
Source: Mod Pathol. 2021 Nov 20;35(5):632–9. doi: 10.1038/s41379-021-00954-z (PMC9042706; doi:10.1038/s41379-021-00954-z)
Supplement: Supplementary file 1 — Supplementary Appendix [file 41379_2021_954_MOESM1_ESM.docx]

**Supplementary Appendix**

This appendix has been provided by the authors to give readers additional information about their work.

**Table of Contents**

**Supplementary Methods4**

**Determination of tumor cell content 4**

**Building the multiclass predictor 5**

**Supplementary Figures8**

**Supplementary Figure S1 8**

**Supplementary Figure S2 9**

**Supplementary Figure S3 10**

**Supplementary Tables11**

**Supplementary Table S1 11**

**Supplementary Table S2 12**

**Supplementary Table S3 22**

**Supplementary Table S4 23**

**Supplementary Table S5 68**

**Supplementary Table S6 70**

**Supplementary Table S7 71**

**Supplementary Table S8 73**

**Supplementary Table S9 123**

**Supplementary Table S10 125**

**Supplementary Table S11 126**

**Supplementary Table S12 128**

**Supplementary Table S13 129**

**Supplementary Methods**

**Determination of tumor cell content**

Fresh tissues were available in most samples of the training and validation cohort. For samples with fresh tissue available, tumor cell content was determined using the flow cytometry method in fresh samples. The tumor cell content is defined as the ratio of the number of tumor cells to the number of total nucleated cells. We retrospectively collected these results from previous studies. For samples only with FFPE slides available, tumor cell content was determined manually using Leica microscopes and imaging system as previously described[**^1^**](#_ENREF_1). The software can select several random high-power fields of a slide. Then, the central areas of these fields were used for visually counting tumor and non-tumor cells according to their specific staining patterns. Both cells were marked manually and counted by the software. For each visual field, counting was continued until a total of at least 200 cells was reached. The percentage of tumor cells relative to tumor-adjacent cells such as stromal cells, inflammatory infiltrate and preexisting epithelial cells was independently estimated by 3 to 7 pathologists. The estimates were scored in categories of 0–10%, 11–20%, 21-30%, 31–40%, 41–50%, 51–60%, 61–70%, 71–80%, 81–90%, and 91–100% tumor cells. Then, we averaged the intervals, and the mean value of the interval was determined to be the final tumor cell content of the sample.

**Building the multiclass predictor**

Like the approach described by Navarro et al[**^2^**](#_ENREF_2), we split the multiclass classification problem into a cascade of binary classifiers which worked as the following steps:

1) Selected the most distinguishable (see below in step 5) SBCLN entity to discriminate.

2) Selected cases for training model and divide them into two groups: i) cases of the selected entity; ii) the rest samples, including the cases belonging to remaining SBCLN subgroups and all 102 non-malignant control samples. We divided the control samples into 3 entities, including peripheral blood/bone marrow, lymph node/Waldeyer’s ring, and extranodal tissues.

3) Built a binary classifier based on random forest algorithm, using standard parameters. This classifier was trained on cases mentioned in step 2. Thus, importance Gini Index of each gene, which was associated with the difference in gene expression between two groups, could be generated from the model.

4) To refine the classifier with optimal number of genes, we iteratively selected top *k* (*k* = 1, 2, ……) genes with highest Importance Gini Index to reconstruct the random forest model. Leave-one-out cross-validation strategy was used to evaluate the classifier. It should be noted that only upregulated genes were included in model reconstruction. In other words, the average expression level of a candidate gene in selected SBCLN entity should higher than that in any other SBCLN entity or one of the three control entities. Moreover, genes selected as markers of a previously discriminated SBCLN entity could be also used for subsequent classifiers. The accuracy of the classifier using the top *k* genes was valued by ACC

ACC(*k*) = $\sum_{n} \left( \mu_{n}\cdot p_{n} \right)$

Where *μ_n_* = 1 if sample *n* was correctly classified, *μ_n_* = -1 if sample *n* was misclassified, and *p_n_* represents the possibility of sample *n* belonging to the selected entity. The iteration was recommended halted in the minimum *k* ^th^ gene (*k*≥3) if

ACC(*m*)<ACC(*k*), ∀*m*<*k*, and

Max{ACC(*n*)}<(1+5%)·ACC(*k*), ∀*n*>*k*

5) Davies-Bouldin Index (DBI) is a well-performed metric for internal evaluating clustering algorithms. Generally, lower DBI represented more distinguishable of a SBCLN entity. DBI of each SBCLN entity was calculated by a one-vs-rest analysis. The order of a SBCLN entity to discriminate was decided according to DBI of the entity but would be postponed if no top *k* genes met the criteria described in step 4.

6) Removed all samples from the selected entity and repeat previous steps until all the SBCLN entities were well identified, or >50% of cases of each remaining SBCLN entity cannot be determined.

**References**

1 Smits A J, Kummer J A, de Bruin P C, Bol M, van den Tweel J G, Seldenrijk K A *et al.* The estimation of tumor cell percentage for molecular testing by pathologists is not accurate. Modern pathology : an official journal of the United States and Canadian Academy of Pathology, Inc 27, 168-174 (2014).

2 Navarro A, Clot G, Martinez-Trillos A, Pinyol M, Jares P, Gonzalez-Farre B *et al.* Improved classification of leukemic B-cell lymphoproliferative disorders using a transcriptional and genetic classifier. Haematologica 102, e360-e363 (2017).

**Supplementary Figures**

**
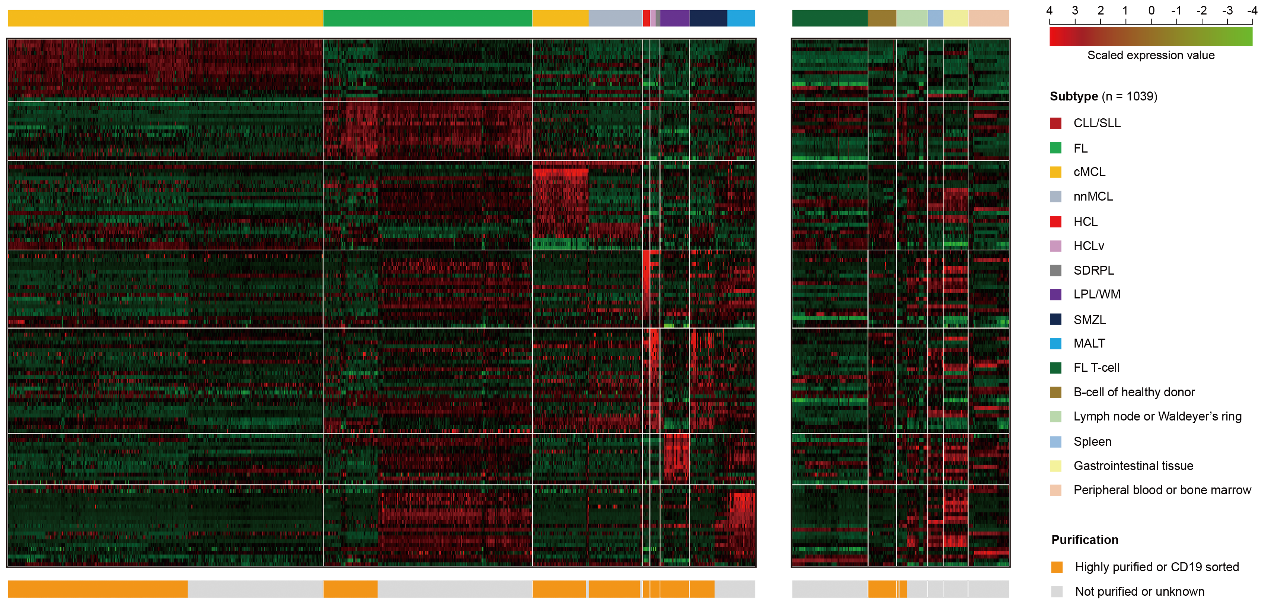
**

**Supplementary Figure S1. Hierarchical clustering of expression profiling of 1039 SBCLN and control cases.** Gene expression profiling of 1039 SBCLN and control samples from 27 GEO datasets was investigated and normalized simultaneously and targeted genes were selected to prepare a heatmap. Each column represents an SBCLN or control case, and each row represents a variable gene in the heatmap (red: high expression, green: low expression; the expression was log_2_ transformed and scaled by *z* statistics for each gene). Cases were clustered by their entities, and variable genes were ordered and clustered according to **Supplementary Table S5**.


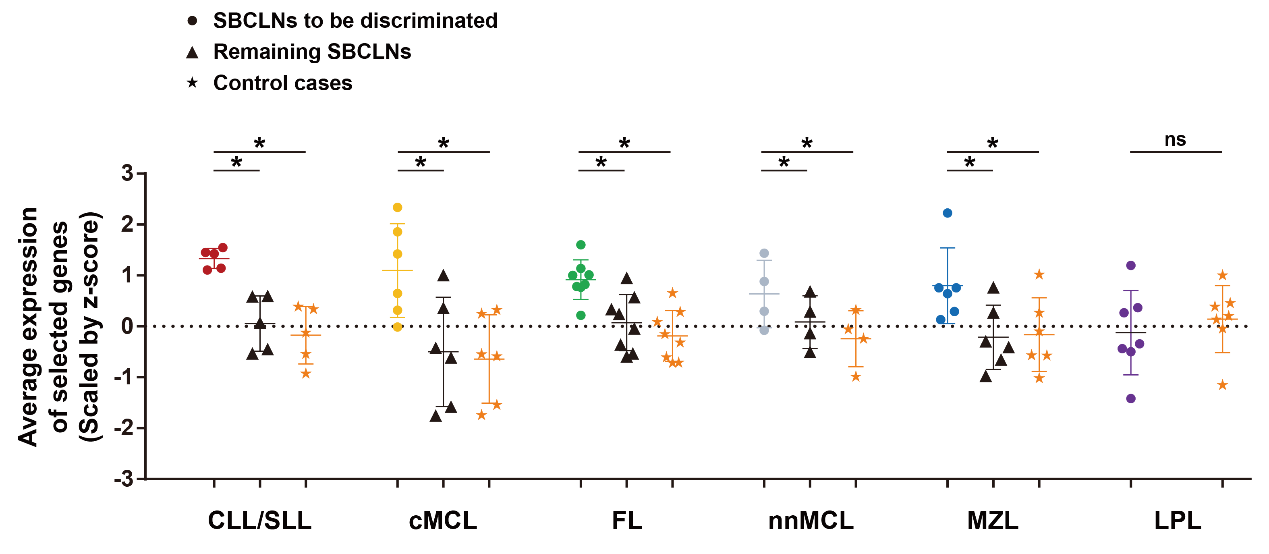


**Supplementary Figure S2.** Average expression of selected genes included in refined subset. Expression was scaled by *z* statistics for each gene. Each SBCLN entity to be determined was marked as colored circle. The remaining SBCLN entities in each step of discrimination was illustrated as black triangle. Control cases were showed as orange stars. * significant statistical differences.

**
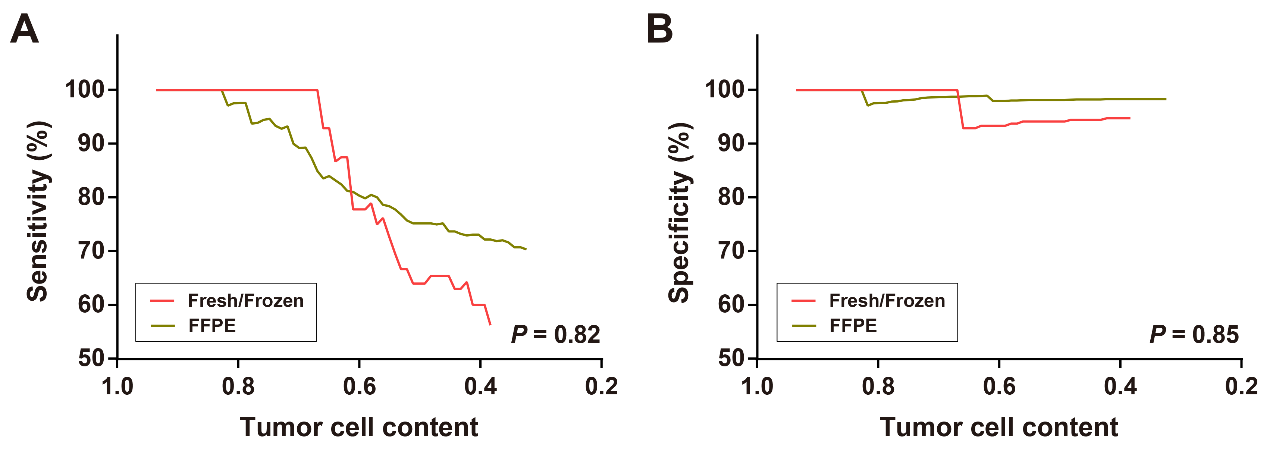
**

**Supplementary Figure S3. The performance of the model in fresh/frozen samples and FFPE samples. A)** The cumulative sensitivity. **(B)** The cumulative specificity.

**Supplementary Tables**

**Supplementary Table S1**. **Entities of SBCLN cases included in our study according to WHO 2016 classification**

| **Entity** | **WHO 2016 classification** | **Training cohort** | **Validation cohort** |
| --- | --- | --- | --- |
| **CLL/SLL** | CLL/SLL | 7 | 14 |
| **cMCL** | MCL (cMCL) | 9 | 27 |
| **nnMCL** | MCL (nnMCL) | 4 | 3 |
| **FL** | FL (grade 1/2) | 13 | 60 |
|  | FL (grade 3 or partial transformed) | 0 | 3 |
|  | Pediatric-type FL | 0 | 2 |
| **MZL** | SMZL | 3 | 2 |
|  | MALTL | 10 | 48 |
|  | NMZL | 6 | 14 |
|  | MZL (partial transformed) | 0 | 3 |
| **LPL/WM** | LPL/WM | 5 | 17 |
| **Other SBCLNs** | HCL | 0 | 2 |

Abbreviations: CLL/SLL, chronic lymphocytic leukemia/small lymphocytic lymphoma; cMCL, conventional mantle cell lymphoma; MCL, mantle cell lymphoma; nnMCL, leukemic non-nodal mantle cell lymphoma; FL, follicular lymphoma; MZL, marginal zone lymphoma; SMZL, splenic marginal zone lymphoma; MALTL, extranodal marginal zone lymphoma of mucosa-associated lymphoid tissue; NMZL, nodal marginal zone lymphoma; LPL/WM, lymphoplasmacytic lymphoma/Waldenström’s macroglobulinemia; HCL, hairy cell leukemia.

**Supplementary Table S2. Patient demographic data and disease characteristics of 57 SBCLNs in training cohort**

| **ID** | **Gender** | **Age** | **Diagnosis** | **Entity** | **Sample type** | **Location^§^** | **Immunophenotype** | **Cytogenetic results*** | **Genetic variation*** |
| --- | --- | --- | --- | --- | --- | --- | --- | --- | --- |
| 1 | Male | 35 | FL (grade 1/2) | FL | FFPE | Lymph node | CD5 (-), CD10 (+), CD19 (+), CD20 (+), CD79α (+), CyclinD1 (-), SOX11 (-), CD23 (+), CD38 (-), CD43 (-), BCL-2 (+), BCL-6 (+), Ki-67 (Li:40%) | *IGH-BCL2* translocation | *EZH2* Y646N |
| 2 | Female | 66 | FL (grade 1/2) | FL | FFPE | Lymph node | CD5 (-), CD10 (+), CD19 (+), CD20 (+), CD79α (+), CyclinD1 (-), SOX11 (-), CD23 (+), CD38 (-), CD43 (-), BCL-2 (+), BCL-6 (+), Ki-67 (Li:30%) | *IGH-BCL2* translocation | *EZH2* Y646N |
| 3 | Male | 49 | CLL/SLL | CLL/SLL | FFPE | Lymph node | CD5 (+), CD10 (-), CD19 (+), CD20 (+), CD79α (+), CyclinD1 (-), SOX11 (-), CD23 (+), CD38 (-), CD43 (+), BCL-2 (+), BCL-6 (-), Ki-67 (Li:50%) | - |  |
| 4 | Male | 48 | FL (grade 1/2) | FL | FFPE | Lymph node | CD5 (-), CD10 (+), CD19 (+), CD20 (+), CD79α (+), CyclinD1 (-), SOX11 (-), CD23 (+), CD38 (-), CD43 (-), BCL-2 (+), BCL-6 (+), Ki-67 (Li:40%) | *IGH-BCL2* translocation |  |
| 5 | Female | 59 | NMZL | MZL | FFPE | Lymph node | CD5 (-), CD10 (-), CD19 (+), CD20 (+), CD79α (+), CyclinD1 (-), SOX11 (-), CD23 (-), CD38(+), CD43 (+), BCL-2 (+), BCL-6 (+), Ki-67 (Li:30%) | - | *MYD88* L265P |
| 6 | Female | 26 | MALTL | MZL | FFPE | Palate | CD5 (-), CD10 (-), CD19 (+), CD20 (+), CD79α (+), CyclinD1 (-), SOX11 (-), CD23 (-), CD38 (-), CD43 (-), BCL-2 (+), BCL-6 (-), Ki-67 (Li:10%) | - |  |
| 7 | Female | 64 | SMZL | MZL | Fresh | Cell | CD5 (-), CD10 (-), CD19 (+), CD20 (+), CD79α (+), CyclinD1 (-), SOX11 (-), CD23 (-), CD38 (-), CD43 (+), BCL-2 (-), BCL-6 (-), Ki-67 (Li:10%) | - |  |
| 8 | Male | 51 | NMZL | MZL | FFPE | Lymph node | CD5 (-), CD10 (-), CD19 (+), CD20 (+), CD79α (+), CyclinD1 (-), SOX11 (-), CD23 (-), CD38 (-), CD43 (-), BCL-2 (+), BCL-6 (-), Ki-67 (Li:20%) | - |  |
| 9 | Female | 22 | MALTL | MZL | FFPE | Mediastinum | CD5 (-), CD10 (-), CD19 (+), CD20 (+), CD79α (+), CyclinD1 (-), SOX11 (-), CD23 (-), CD38 (-), BCL-2 (+), BCL-6 (-), Ki-67 (Li:15%) | *MALT1* translocation |  |
| 10 | Female | 37 | FL (grade 1/2) | FL | FFPE | Lymph node | CD5 (-), CD10 (+), CD19 (+), CD20 (+), CD79α (+), CyclinD1 (-), SOX11 (-), CD23 (+), CD38 (-), CD43 (-), BCL-2 (+), BCL-6 (+), | *IGH-BCL2* translocation |  |
| 11 | Female | 54 | NMZL | MZL | FFPE | Lymph node | CD5 (-), CD10 (-), CD19 (+), CD20 (+), CD79α (+), CyclinD1 (-), SOX11 (-), CD23 (-), CD38 (-), CD43 (+), BCL-2 (+), BCL-6 (-), Ki-67 (Li:15%) | - | *BRAF* V600E |
| 12 | Female | 52 | MALTL | MZL | FFPE | Parotid gland | CD5 (-), CD10 (-), CD19 (+), CD20 (+), CD79α (+), CyclinD1 (-), SOX11 (-), CD23 (-), CD38 (-), BCL-2 (+), BCL-6 (-), Ki-67 (Li:5%) | - |  |
| 13 | Male | 59 | CLL/SLL | CLL/SLL | FFPE | Lymph node | CD5 (+), CD10 (-), CD19 (+), CD20 (+), CD79α (+), CyclinD1 (-), SOX11 (-), CD23 (+), CD38 (-), CD43 (+), BCL-2 (+), BCL-6 (-), Ki-67 (Li:40%) | - |  |
| 14 | Male | 46 | NMZL | MZL | FFPE | Lymph node | CD5 (-), CD10 (-), CD19 (+), CD20 (+), CD79α (+), CyclinD1 (-), SOX11 (-), CD23 (-), CD38 (-), CD43 (+), BCL-2 (+), BCL-6 (-), Ki-67 (Li:30%) | - |  |
| 15 | Male | 67 | nnMCL | nnMCL | FFPE | Ocular adnexa | CD5 (+), CD10 (-), CD19 (+), CD20 (+), CD79α (+), CyclinD1 (+), SOX11 (-), CD23 (-), CD38 (-), CD43 (+), BCL-2 (+), BCL-6 (-), Ki-67 (Li:10%) | *IGH-CCND1* translocation |  |
| 16 | Male | 55 | SMZL | MZL | FFPE | Spleen | CD5 (-), CD10 (-), CD19 (+), CD20 (+), CD79α (+), CyclinD1 (-), SOX11 (-), CD23 (-), CD38 (-), CD43 (-), BCL-2 (-), BCL-6 (-), Ki-67 (Li:5%) | - |  |
| 17 | Female | 46 | MALTL | MZL | FFPE | Parotid gland | CD5 (-), CD10 (-), CD19 (+), CD20 (+), CD79α (+), CyclinD1 (-), SOX11 (-), CD23 (-), CD38 (-), BCL-2 (+), BCL-6 (-), Ki-67 (Li:10%) | Other *IGH* translocation |  |
| 18 | Male | 64 | cMCL | cMCL | FFPE | Lymph node | CD5 (+), CD10 (-), CD19 (+), CD20 (+), CD79α (+), CyclinD1 (+), SOX11 (+), CD38 (-), CD43 (+), BCL-2 (+), BCL-6 (-), Ki-67 (Li:30%) | *IGH-CCND1* translocation |  |
| 19 | Male | 46 | cMCL | cMCL | FFPE | Lymph node | CD5 (+), CD10 (-), CD19 (+), CD20 (+), CD79α (+), CyclinD1 (+), SOX11 (+), CD38 (-), CD43 (+), BCL-2 (+), BCL-6 (-), Ki-67 (Li:20%) | *IGH-CCND1* translocation |  |
| 20 | Male | 53 | SMZL | MZL | FFPE | Spleen | CD5 (-), CD10 (-), CD19 (+), CD20 (+), CD79α (+), CyclinD1 (-), SOX11 (-), CD23 (-), CD38 (-), CD43 (-), BCL-2 (-), BCL-6 (-), Ki-67 (Li:20%) | del(7q) | *NOTCH2* R2400* |
| 21 | Female | 66 | cMCL | cMCL | FFPE | Lymph node | CD5 (+), CD10 (-), CD19 (+), CD20 (+), CD79α (+), CyclinD1 (+), SOX11 (+), CD38 (-), CD43 (+), BCL-2 (+), BCL-6 (-), Ki-67 (Li:40%) | *IGH-CCND1* translocation |  |
| 22 | Female | 52 | MALTL | MZL | FFPE | Parotid gland | CD5 (-), CD10 (-), CD19 (+), CD20 (+), CD79α (+), CyclinD1 (-), SOX11 (-), CD23 (-), CD38 (-), CD43 (-), BCL-2 (+), BCL-6 (-), Ki-67 (Li:15%) | - | *NOTCH2* R2400* |
| 23 | Female | 65 | CLL/SLL | CLL/SLL | FFPE | Lymph node | CD5 (+), CD10 (-), CD19 (+), CD20 (+), CD79α (+), CyclinD1 (-), SOX11 (-), CD23 (+), CD38 (-), CD43 (+), BCL-2 (+), BCL-6 (-), Ki-67 (Li:20%) | +12 |  |
| 24 | Female | 30 | MALTL | MZL | FFPE | Parotid gland | CD5 (-), CD10 (-), CD19 (+), CD20 (+), CD79α (+), CyclinD1 (-), SOX11 (-), CD23 (-), CD38 (-), BCL-2 (+), BCL-6 (-), Ki-67 (Li:35%) | - |  |
| 25 | Male | 68 | CLL/SLL | CLL/SLL | FFPE | Lymph node | CD5 (-), CD10 (-), CD19 (+), CD20 (+), CD79α (+), CyclinD1 (-), SOX11 (-), CD23 (+), CD38 (-), CD43 (+), BCL-2 (+), BCL-6 (-), Ki-67 (Li:30%) | +12 |  |
| 26 | Female | 32 | MALTL | MZL | FFPE | Ocular adnexa | CD5 (-), CD10 (-), CD19 (+), CD20 (+), CD79α (+), CyclinD1 (-), SOX11 (-), CD23 (-), CD38 (-), CD43 (-), BCL-2 (+), BCL-6 (-), Ki-67 (Li:5%) | - |  |
| 27 | Male | 46 | NMZL | MZL | FFPE | Lymph node | CD5 (-), CD10 (-), CD19 (+), CD20 (+), CD79α (+), CyclinD1 (-), SOX11 (-), CD23 (-), CD38 (-), CD43 (+), BCL-2 (+), BCL-6 (-), Ki-67 (Li:10%) | - | *NOTCH2* R2400* |
| 28 | Female | 44 | CLL/SLL | CLL/SLL | FFPE | Lymph node | CD5 (-), CD10 (-), CD19 (+), CD20 (+), CD79α (+), CyclinD1 (-), SOX11 (-), CD23 (+), CD38 (-), CD43 (+), BCL-2 (+), BCL-6 (-), Ki-67 (Li:20%) | - | MYD88 L265P |
| 29 | Male | 46 | FL (grade 1/2) | FL | FFPE | Lymph node | CD5 (-), CD10 (+), CD19 (+), CD20 (+), CD79α (+), CyclinD1 (-), SOX11 (-), CD23 (+), CD38 (-), CD43 (-), BCL-2 (+), BCL-6 (+), Ki-67 (Li:25%) | *IGH-BCL2* translocation |  |
| 30 | Male | 52 | CLL/SLL | CLL/SLL | FFPE | Lymph node | CD5 (+), CD10 (-), CD19 (+), CD20 (+), CD79α (+), CyclinD1 (-), SOX11 (-), CD23 (+), CD38 (-), CD43 (+), BCL-2 (+), BCL-6 (-), Ki-67 (Li:30%) | - |  |
| 31 | Female | 45 | MALTL | MZL | FFPE | Parotid gland | CD5 (-), CD10 (-), CD19 (+), CD20 (+), CD79α (+), CyclinD1 (-), SOX11 (-), CD23 (-), CD38 (-), CD43 (+), BCL-2 (+), BCL-6 (+) | - |  |
| 32 | Male | 53 | CLL/SLL | CLL/SLL | FFPE | Lymph node | CD5 (-), CD10 (-), CD19 (+), CD20 (+), CD79α (+), CyclinD1 (-), SOX11 (-), CD23 (+), CD38 (-), CD43 (+), BCL-2 (+), BCL-6 (-), Ki-67 (Li:20%) | - |  |
| 33 | Male | 70 | cMCL | cMCL | FFPE | Lymph node | CD5 (+), CD10 (-), CD19 (+), CD20 (+), CD79α (+), CyclinD1 (+), SOX11 (+), CD38 (-), CD43 (+), BCL-2 (+), BCL-6 (-), Ki-67 (Li:20%) | *IGH-CCND1* translocation; +12 |  |
| 34 | Female | 39 | MALTL | MZL | FFPE | Mediastinum | CD5 (-), CD10 (-), CD19 (+), CD20 (+), CD79α (+), CyclinD1 (-), SOX11 (-), CD23 (-), CD38 (+), BCL-2 (+), BCL-6 (-), Ki-67 (Li:5%) | - |  |
| 35 | Female | 79 | NMZL | MZL | FFPE | Lymph node | CD5 (-), CD10 (-), CD19 (+), CD20 (+), CD79α (+), CyclinD1 (-), SOX11 (-), CD23 (-), CD38 (-), BCL-2 (+), BCL-6 (-), Ki-67 (Li:10%) | - |  |
| 36 | Female | 56 | MALTL | MZL | FFPE | Ocular adnexa | CD5 (-), CD10 (-), CD19 (+), CD20 (+), CD79α (+), CyclinD1 (-), SOX11 (-), CD23 (-), CD38 (-), BCL-2 (+), BCL-6 (-), Ki-67 (Li:20%) | - |  |
| 37 | Male | 66 | cMCL | cMCL | FFPE | Lymph node | CD5 (+), CD10 (-), CD19 (+), CD20 (+), CD79α (+), CyclinD1 (+), SOX11 (+), CD38 (-), CD43 (+), BCL-2 (+), BCL-6 (-), Ki-67 (Li:10%) | *IGH-CCND1* translocation |  |
| 38 | Male | 62 | cMCL | cMCL | FFPE | Lymph node | CD5 (+), CD10 (-), CD19 (+), CD20 (+), CD79α (+), CyclinD1 (+), SOX11 (+), CD38 (-), CD43 (+), BCL-2 (+), BCL-6 (-), Ki-67 (Li:30%) | *IGH-CCND1* translocation |  |
| 39 | Female | 58 | FL (grade 1/2) | FL | FFPE | Lymph node | CD5 (-), CD10 (+), CD19 (+), CD20 (+), CD79α (+), CyclinD1 (-), SOX11 (-), CD23 (+), CD38 (-), CD43 (-), BCL-2 (+), BCL-6 (+), Ki-67 (Li:30%) | *IGH-BCL2* translocation |  |
| 40 | Female | 43 | FL (grade 1/2) | FL | FFPE | Lymph node | CD5 (-), CD10 (+), CD19 (+), CD20 (+), CD79α (+), CyclinD1 (-), SOX11 (-), CD38 (-), BCL-2 (+), BCL-6 (+) | *IGH-BCL2* translocation | *EZH2* Y646N |
| 41 | Male | 48 | nnMCL | nnMCL | Fresh | Cell | CD5 (+), CD10 (-), CD19 (+), CD20 (+), CD79α (+), CyclinD1 (+), SOX11 (-), CD38 (-), CD43 (+), BCL-2 (+), BCL-6 (-), Ki-67 (Li:20%) | *IGH-CCND1* translocation |  |
| 42 | Male | 56 | nnMCL | nnMCL | FFPE | Gastric mucosa | CD5 (+), CD10 (-), CD19 (+), CD20 (+), CD79α (+), CyclinD1 (+), SOX11 (-), CD38 (-), CD43 (+), BCL-2 (+), BCL-6 (-), Ki-67 (Li:10%) | *IGH-CCND1* translocation |  |
| 43 | Male | 61 | nnMCL | nnMCL | Fresh | Cell | CD5 (+), CD10 (-), CD19 (+), CD20 (+), CD79α (+), CyclinD1 (+), SOX11 (-), CD38 (-), CD43 (+), BCL-2 (+), BCL-6 (-), Ki-67 (Li:0%) | *IGH-CCND1* translocation |  |
| 44 | Male | 60 | cMCL | cMCL | FFPE | Lymph node | CD5 (+), CD10 (-), CD19 (+), CD20 (+), CD79α (+), CyclinD1 (+), SOX11 (+), CD38 (-), CD43 (+), BCL-2 (+), BCL-6 (-), Ki-67 (Li:20%) | *IGH-CCND1* translocation |  |
| 45 | Female | 32 | FL (grade 1/2) | FL | FFPE | Lymph node | CD5 (-), CD10 (+), CD19 (+), CD20 (+), CD79α (+), CyclinD1 (-), SOX11 (-), CD38 (-), BCL-2 (+), BCL-6 (+) | *IGH-BCL2* translocation |  |
| 46 | Male | 50 | cMCL | cMCL | FFPE | Lymph node | CD5 (+), CD10 (-), CD19 (+), CD20 (+), CD79α (+), CyclinD1 (+), SOX11 (+), CD38 (-), CD43 (+), BCL-2 (+), BCL-6 (-), Ki-67 (Li:50%) | *IGH-CCND1* translocation |  |
| 47 | Male | 46 | cMCL | cMCL | FFPE | Lymph node | CD5 (+), CD10 (-), CD19 (+), CD20 (+), CD79α (+), CyclinD1 (+), SOX11 (+), CD38 (-), CD43 (+), BCL-2 (+), BCL-6 (-), Ki-67 (Li:40%) | *IGH-CCND1* translocation |  |
| 48 | Female | 43 | FL (grade 1/2) | FL | FFPE | Lymph node | CD5 (-), CD10 (+), CD19 (+), CD20 (+), CD79α (+), CyclinD1 (-), SOX11 (-), CD38 (-), BCL-2 (+), BCL-6 (+) | *IGH-BCL2* translocation | *EZH2* Y646N |
| 49 | Male | 72 | FL (grade 1/2) | FL | FFPE | Lymph node | CD5 (-), CD10 (+), CD19 (+), CD20 (+), CD79α (+), CyclinD1 (-), SOX11 (-), CD38 (-), BCL-2 (+), BCL-6 (+) | *IGH-BCL2* translocation |  |
| 50 | Male | 55 | FL (grade 1/2) | FL | FFPE | Lymph node | CD5 (-), CD10 (+), CD19 (+), CD20 (+), CD79α (+), CyclinD1 (-), SOX11 (-), CD38 (-), BCL-2 (+), BCL-6 (+) | *IGH-BCL2* translocation |  |
| 51 | Male | 53 | FL (grade 1/2) | FL | FFPE | Lymph node | CD5 (-), CD10 (+), CD19 (+), CD20 (+), CD79α (+), CyclinD1 (-), SOX11 (-), CD38 (-), BCL-2 (+), BCL-6 (+) | *IGH-BCL2* translocation |  |
| 52 | Female | 61 | FL (grade 1/2) | FL | FFPE | Lymph node | CD5 (-), CD10 (+), CD19 (+), CD20 (+), CD79α (+), CyclinD1 (-), SOX11 (-), CD38 (-), BCL-2 (+), BCL-6 (+) | *IGH-BCL2* translocation | *EZH2* Y646S |
| 53 | Male | 48 | LPL/WM | LPL/WM | Fresh | Cell | CD5 (-), CD10 (-), CD19 (+), CD20 (+), CD79α (+), CyclinD1 (-), SOX11 (-), CD23 (-), CD38 (+), BCL-2 (-) | - | *MYD88* L265P |
| 54 | Male | 73 | LPL/WM | LPL/WM | Fresh | Cell | CD5 (-), CD10 (-), CD19 (+), CD20 (+), CD79α (+), CyclinD1 (-), SOX11 (-), CD23 (-), CD38 (+), BCL-2 (-) | - | *MYD88* L265P |
| 55 | Male | 81 | LPL/WM | LPL/WM | Fresh | Cell | CD5 (-), CD10 (-), CD19 (+), CD20 (+), CD79α (+), CyclinD1 (-), SOX11 (-), CD23 (-), CD38 (+), BCL-2 (-) | - | *MYD88* L265P;  *CXCR4* S338* |
| 56 | Female | 71 | LPL/WM | LPL/WM | Fresh | Cell | CD5 (-), CD10 (-), CD19 (+), CD20 (+), CD79α (+), CyclinD1 (-), SOX11 (-), CD23 (-), CD38 (+), BCL-2 (-) | - | *MYD88* L265P |
| 57 | Female | 68 | LPL/WM | LPL/WM | Fresh | Cell | CD5 (-), CD10 (-), CD19 (+), CD20 (+), CD79α (+), CyclinD1 (-), SOX11 (-), CD23 (-), CD38 (+), BCL-2 (-) | - | *MYD88* L265P |

Abbreviations: CLL/SLL, chronic lymphocytic leukemia/small lymphocytic lymphoma; cMCL, conventional mantle cell lymphoma; MCL, mantle cell lymphoma; nnMCL, leukemic non-nodal mantle cell lymphoma; FL, follicular lymphoma; MZL, marginal zone lymphoma; SMZL, splenic marginal zone lymphoma; MALTL, extranodal marginal zone lymphoma of mucosa-associated lymphoid tissue; NMZL, nodal marginal zone lymphoma; LPL/WM, lymphoplasmacytic lymphoma/Waldenström’s macroglobulinemia; FFPE, formalin-fixed paraffin-embedded.

§ Cells for RNA extraction were previously sorted by anti-CD19-coated immunomagnetic beads.

* Chromosomal aberrations and somatic mutations for ancillary diagnosis of SBCLN in our study are listed in Supplementary Table S5.

**Supplementary Table S3. Details of 102 non-malignant control samples in training cohort**

| **Non-malignant control samples** | **Subgroup** | **Sample type** | **Count** |
| --- | --- | --- | --- |
| Peripheral blood/Bone marrow | Peripheral blood/Bone marrow | Fresh | 29 |
| Reactive lymph node hyperplasia | Lymph node/Waldeyer's ring | FFPE | 21 |
| Reactive tonsil | Lymph node/Waldeyer's ring | FFPE | 11 |
| Gastrointestinal inflammation | Extranodal tissues | FFPE | 17 |
| Splenomegaly | Extranodal tissues | FFPE | 12 |
| Thymic hyperplasi | Extranodal tissues | FFPE | 3 |
| Sialadenitis | Extranodal tissues | FFPE | 5 |
| Sinusitis | Extranodal tissues | FFPE | 4 |

Abbreviations: FFPE, formalin-fixed paraffin-embedded.

**Supplementary Table S4. Patient demographic data and disease characteristics of testing cohort**

| **ID** | **Sex** | **Age** | **Initial histopathologic diagnosis** | **Chromosomal aberrations** | **Recurrent mutations** | **Final entity** | **Molecular entity** | **Sample type** | **Location** | **Tumor content** | **Immunohistochemistry** |
| --- | --- | --- | --- | --- | --- | --- | --- | --- | --- | --- | --- |
| 1 | Male | 48 | MALTL | - | - | MZL | MZL | FFPE | Liver | 0.82 | CD5 (-), CD10 (-), CD19 (+), CD20 (+), CD79α (+), CyclinD1 (-), SOX11 (-), CD23 (-), CD38 (-), CD43 (-), BCL-2 (+), BCL-6 (-) |
| 2 | Female | 53 | MALTL | - | - | MZL | MZL | FFPE | Parotid gland | 0.47 | CD5 (-), CD10 (-), CD19 (+), CD20 (+), CD79α (+), CyclinD1 (-), SOX11 (-), CD23 (-), CD38 (+), BCL-2 (+), BCL-6 (-), Ki-67 (Li:20%) |
| 3 | Male | 44 | MALTL | *BIRC3-MALT1* | - | MZL | MZL | FFPE | Gastric mucosa | 0.72 | CD5 (-), CD10 (-), CD19 (+), CD20 (+), CD79α (+), CyclinD1 (-), SOX11 (-), CD23 (-), CD38 (-), CD43 (-), BCL-2 (+), BCL-6 (-), Ki-67 (Li:5%) |
| 4 | Male | 51 | MALTL | - | - | MZL | MZL | FFPE | Ocular adnexa | 0.85 | CD5 (-), CD10 (-), CD19 (+), CD20 (+), CD79α (+), CyclinD1 (-), SOX11 (-), CD23 (-), CD38 (-), CD43 (-), BCL-2 (+), BCL-6 (-), Ki-67 (Li:5%) |
| 5 | Female | 34 | MALTL | - | - | MZL | MZL | FFPE | Parotid gland | 0.76 | CD5 (-), CD10 (-), CD19 (+), CD20 (+), CD79α (+), CyclinD1 (-), SOX11 (-), CD23 (-), CD38 (-), BCL-2 (+), BCL-6 (-), Ki-67 (Li:10%) |
| 6 | Male | 69 | cMCL | *IGH-CCND1* | - | cMCL | cMCL | FFPE | Lymph node | 0.65 | CD5 (+), CD10 (-), CD19 (+), CD20 (+), CD79α (+), CyclinD1 (+), SOX11 (+), CD38 (-), CD43 (+), BCL-2 (+), BCL-6 (-), Ki-67 (Li:20%) |
| 7 | Female | 50 | FL (grade 1/2) | *IGH-BCL2* | - | FL | FL | FFPE | Lymph node | 0.92 | CD5 (-), CD10 (+), CD19 (+), CD20 (+), CD79α (+), CyclinD1 (-), SOX11 (-), CD23 (+), CD38 (-), CD43 (-), BCL-2 (+), BCL-6 (+) |
| 8 | Female | 51 | CLL/SLL | *-* | - | CLL/SLL | CLL/SLL | FFPE | Lymph node | 0.83 | CD5 (+), CD10 (-), CD19 (+), CD20 (+), CD79α (+), CyclinD1 (-), SOX11 (-), CD23 (+), CD38 (-), CD43 (+), BCL-2 (+), BCL-6 (-), Ki-67 (Li: 15%) |
| 9 | Female | 41 | FL (grade 1/2) | *IGH-BCL2* | *EZH2* Y646S | FL | FL | FFPE | Lymph node | 0.66 | CD5 (-), CD10 (+), CD19 (+), CD20 (+), CD79α (+), CyclinD1 (-), SOX11 (-), CD23 (+), CD38 (-), CD43 (-), BCL-2 (+), BCL-6 (-), Ki-67 (Li:5%) |
| 10 | Male | 54 | CLL/SLL | - | *MYD88* L265P | CLL/SLL | CLL/SLL | FFPE | Lymph node | 0.67 | CD5 (-), CD10 (-), CD19 (+), CD20 (+), CD79α (+), CyclinD1 (-), SOX11 (-), CD23 (+), CD38 (-), CD43 (+), BCL-2 (+), BCL-6 (-), Ki-67 (Li: 10%) |
| 11 | Female | 51 | FL (grade 1/2) | *IGH-BCL2* | *EZH2* Y646N | FL | FL | FFPE | Mesentery | 0.86 | CD5 (-), CD10 (+), CD19 (+), CD20 (+), CD79α (+), CyclinD1 (-), SOX11 (-), CD23 (+), CD38 (-), CD43 (-), BCL-2 (+), BCL-6 (+), Ki-67 (Li:50%) |
| 12 | Male | 49 | NMZL | - | - | MZL | Unclassified | FFPE | Lymph node | 0.45 | CD5 (-), CD10 (-), CD19 (+), CD20 (+), CD79α (+), CyclinD1 (-), SOX11 (-), CD23 (-), CD38 (+), CD43 (-), BCL-2 (+), BCL-6 (-), Ki-67 (Li:30%) |
| 13 | Female | 71 | MALTL | *BIRC3-MALT1* | - | MZL | MZL | FFPE | Thyroid gland | 0.7 | CD5 (-), CD10 (-), CD19 (+), CD20 (+), CD79α (+), CyclinD1 (-), SOX11 (-), CD23 (-), CD38 (-), CD43 (+), BCL-2 (+), BCL-6 (+) |
| 14 | Male | 76 | CLL/SLL | - | - | CLL/SLL | Unclassified | FFPE | Prostate | 0.74 | CD5 (+), CD10 (-), CD19 (+), CD20 (+), CD79α (+), CyclinD1 (-), SOX11 (-), CD23 (+), CD38 (-), CD43 (+), BCL-2 (+), BCL-6 (-) |
| 15 | Female | 52 | MALTL |  | - | MZL | nnMCL | FFPE | Gastric mucosa | 0.61 | CD5 (-), CD10 (-), CD19 (+), CD20 (+), CD79α (+), CyclinD1 (-), SOX11 (-), CD23 (-), CD38 (-), CD43 (+), BCL-2 (+), BCL-6 (-), Ki-67 (Li:10%) |
| 16 | Male | 79 | NMZL | *IGH-BCL2* | - | FL | FL | FFPE | Lymph node | 0.47 | CD5 (-), CD10 (-), CD19 (+), CD20 (+), CD79α (+), CyclinD1 (-), SOX11 (-), CD23 (+), CD38 (-), CD43 (-), BCL-2 (+), BCL-6 (-), Ki-67 (Li:25%) |
| 17 | Female | 52 | MALTL | *-* | *MYD88* L265P | MZL | MZL | FFPE | Ocular adnexa | 0.58 | CD5 (-), CD10 (-), CD19 (+), CD20 (+), CD79α (+), CyclinD1 (-), SOX11 (-), CD23 (-), CD38 (-), CD43 (-), BCL-2 (+), BCL-6 (-), Ki-67 (Li:10%) |
| 18 | Male | 35 | FL (grade 1/2) | *IGH-BCL2* | *EZH2* Y646S | FL | Unclassified | FFPE | Lymph node | 0.54 | CD5 (-), CD10 (+), CD19 (+), CD20 (+), CD79α (+), CyclinD1 (-), SOX11 (-), CD23 (+), CD38 (-), CD43 (-), BCL-2 (+), BCL-6 (+) |
| 19 | Male | 53 | CLL/SLL | *-* | - | CLL/SLL | CLL/SLL | FFPE | Lymph node | 0.91 | CD5 (+), CD10 (-), CD19 (+), CD20 (+), CD79α (+), CyclinD1 (-), SOX11 (-), CD23 (+), CD38 (-), CD43 (+), BCL-2 (+), BCL-6 (-), Ki-67 (Li:5%) |
| 20 | Male | 53 | cMCL | *IGH-CCND1* | - | cMCL | cMCL | FFPE | Lymph node | 0.73 | CD5 (+), CD10 (-), CD19 (+), CD20 (+), CD79α (+), CyclinD1 (+), SOX11 (+), CD38 (-), CD43 (+), BCL-2 (+), BCL-6 (-), Ki-67 (Li:10%) |
| 21 | Male | 35 | FL (grade 1/2) | *IGH-BCL2* | - | FL | Unclassified | FFPE | Cutaneous tissue | 0.6 | CD5 (-), CD10 (+), CD19 (+), CD20 (+), CD79α (+), CyclinD1 (-), SOX11 (-), CD23 (+), CD38 (-), CD43 (-), BCL-2 (+), BCL-6 (+), Ki-67 (Li:25%) |
| 22 | Male | 69 | cMCL | *IGH-CCND1* | - | cMCL | cMCL | FFPE | Lymph node | 0.9 | CD5 (+), CD10 (-), CD19 (+), CD20 (+), CD79α (+), CyclinD1 (+), SOX11 (+), CD38 (-), CD43 (+), BCL-2 (+), BCL-6 (-), Ki-67 (Li:30%) |
| 23 | Male | 51 | MALTL | *-* | - | MZL | MZL | FFPE | Parotid gland | 0.83 | CD5 (-), CD10 (-), CD19 (+), CD20 (+), CD79α (+), CyclinD1 (-), SOX11 (-), CD23 (-), CD38 (-), BCL-2 (+), BCL-6 (-), Ki-67 (Li:5%) |
| 24 | Female | 52 | MALTL | *-* | - | MZL | MZL | FFPE | Mediastinum | 0.75 | CD5 (-), CD10 (-), CD19 (+), CD20 (+), CD79α (+), CyclinD1 (-), SOX11 (-), CD23 (-), CD38 (-), CD43 (-), BCL-2 (+), BCL-6 (-), Ki-67 (Li:15%) |
| 25 | Female | 34 | FL (grade 1/2) | *IGH-BCL2* | - | FL | FL | FFPE | Pelvic mass | 0.86 | CD5 (-), CD10 (+), CD19 (+), CD20 (+), CD79α (+), CyclinD1 (-), SOX11 (-), CD43 (-), CD38 (-), BCL-2 (+), BCL-6 (+), Ki-67 (Li:30%) |
| 26 | Male | 72 | FL (grade 1/2) | *IGH-BCL2* | - | FL | FL | FFPE | Lymph node | 0.81 | CD5 (-), CD10 (+), CD19 (+), CD20 (+), CD79α (+), CyclinD1 (-), SOX11 (-), CD23 (+), CD38 (-), CD43 (-), BCL-2 (+), BCL-6 (+), Ki-67 (Li:50%) |
| 27 | Male | 27 | FL (grade 1/2) | *IGH-BCL2* | - | FL | Unclassified | FFPE | Parotid gland | 0.42 | CD5 (-), CD10 (+), CD19 (+), CD20 (+), CD79α (+), CyclinD1 (-), SOX11 (-), CD23 (+), CD38 (-), CD43 (+), BCL-2 (+), BCL-6 (+), Ki-67 (Li:40%) |
| 28 | Female | 45 | MALTL | *-* | - | MZL | Unclassified | FFPE | Mediastinum | 0.51 | CD5 (-), CD10 (-), CD19 (+), CD20 (+), CD79α (+), CyclinD1 (-), SOX11 (-), CD23 (-), CD38 (-), CD43 (+), BCL-2 (+), BCL-6 (-), Ki-67 (Li:15%) |
| 29 | Female | 47 | FL (grade 1/2) | *IGH-BCL2* | *EZH2* Y646H | FL | FL | FFPE | Lymph node | 0.56 | CD5 (-), CD10 (+), CD19 (+), CD20 (+), CD79α (+), CyclinD1 (-), SOX11 (-), CD23 (+), CD38 (-), CD43 (-), BCL-2 (+), BCL-6 (+) |
| 30 | Male | 57 | FL (grade 1/2) | *-* | *NOTCH2* R2400* | MZL | MZL | FFPE | Lymph node | 0.42 | CD5 (-), CD10 (+), CD19 (+), CD20 (+), CD79α (+), CyclinD1 (-), SOX11 (-), CD23 (+), CD38 (-), CD43 (+), BCL-2 (+), BCL-6 (+) |
| 31 | Female | 52 | FL (grade 1/2) | *IGH-BCL2* | - | FL | FL | FFPE | Lymph node | 0.92 | CD5 (-), CD10 (+), CD19 (+), CD20 (+), CD79α (+), CyclinD1 (-), SOX11 (-), CD23 (-), CD38 (-), CD43 (-), BCL-2 (+), BCL-6 (+), Ki-67 (Li:15%) |
| 32 | Male | 33 | FL (grade 1/2) | *IGH-BCL2* | *EZH2* Y646N | FL | MZL | FFPE | Lymph node | 0.35 | CD5 (-), CD10 (+), CD19 (+), CD20 (+), CD79α (+), CyclinD1 (-), SOX11 (-), CD23 (-), CD38 (-), CD43 (-), BCL-2 (-), BCL-6 (+) |
| 33 | Female | 52 | FL (grade 1/2) | *IGH-BCL2* | - | FL | FL | FFPE | Mesentery | 0.82 | CD5 (-), CD10 (+), CD19 (+), CD20 (+), CD79α (+), CyclinD1 (-), SOX11 (-), CD23 (-), CD38 (-), CD43 (-), BCL-2 (+), BCL-6 (+), Ki-67 (Li:5%) |
| 34 | Female | 45 | MALTL | *-* | - | MZL | MZL | FFPE | Palate | 0.86 | CD5 (-), CD10 (-), CD19 (+), CD20 (+), CD79α (+), CyclinD1 (-), SOX11 (-), CD23 (-), CD38 (+), CD43 (-), BCL-2 (+), BCL-6 (-), Ki-67 (Li:5%) |
| 35 | Male | 36 | FL (grade 1/2) | *IGH-BCL2* | - | FL | FL | FFPE | Lymph node | 0.78 | CD5 (-), CD10 (+), CD19 (+), CD20 (+), CD79α (+), CyclinD1 (-), SOX11 (-), CD23 (+), CD38 (-), CD43 (-), BCL-2 (+), BCL-6 (+) |
| 36 | Male | 25 | FL (grade 1/2) | *-* | - | FL | Unclassified | FFPE | Lymph node | 0.63 | CD5 (-), CD10 (+), CD19 (+), CD20 (+), CD79α (+), CyclinD1 (-), SOX11 (-), CD38 (-),CD43 (-), BCL-2 (-), BCL-6 (+), Ki-67 (Li:75%) |
| 37 | Male | 47 | cMCL | *IGH-CCND1* | - | cMCL | cMCL | FFPE | Lymph node | 0.63 | CD5 (+), CD10 (-), CD19 (+), CD20 (+), CD79α (+), CyclinD1 (+), SOX11 (+), CD38 (-), CD43 (+), BCL-2 (+), BCL-6 (-), Ki-67 (Li:5%) |
| 38 | Male | 58 | cMCL | *IGH-CCND1*; +12 | - | cMCL | cMCL | FFPE | Lymph node | 0.73 | CD5 (+), CD10 (-), CD19 (+), CD20 (+), CD79α (+), CyclinD1 (+), SOX11 (+), CD38 (-), CD43 (+), BCL-2 (+), BCL-6 (-), Ki-67 (Li:30%) |
| 39 | Female | 60 | MALTL | - | - | MZL | MZL | FFPE | Ocular adnexa | 0.65 | CD5 (+), CD10 (-), CD19 (+), CD20 (+), CD79α (+), CyclinD1 (-), SOX11 (-), CD23 (-), CD38 (-), CD43 (-), BCL-2 (+), BCL-6 (-), Ki-67 (Li:10%) |
| 40 | Female | 56 | MALTL | - | - | MZL | Unclassified | FFPE | Thyroid gland | 0.56 | CD5 (-), CD10 (-), CD19 (+), CD20 (+), CD79α (+), CyclinD1 (-), SOX11 (-), CD23 (-), CD38 (-), BCL-2 (+), BCL-6 (-), Ki-67 (Li:25%) |
| 41 | Male | 34 | MALTL | - | - | MZL | MZL | FFPE | Gastric mucosa | 0.73 | CD5 (-), CD10 (-), CD19 (+), CD20 (+), CD79α (+), CyclinD1 (-), SOX11 (-), CD23 (-), CD38 (-), CD43 (+), BCL-2 (+), BCL-6 (-), Ki-67 (Li:5%) |
| 42 | Male | 61 | FL (grade 1/2) | *IGH-BCL2* | *EZH2* Y646N | FL | Unclassified | FFPE | Retroperitoneum mass | 0.47 | CD5 (-), CD10 (+), CD19 (+), CD20 (+), CD79α (+), CyclinD1 (-), SOX11 (-), CD23 (+), CD38 (-), CD43 (-), BCL-2 (+), BCL-6 (+), Ki-67 (Li:40%) |
| 43 | Female | 72 | SMZL | del(7q) | - | MZL | MZL | FFPE | Spleen | 0.62 | CD5 (-), CD10 (-), CD19 (+), CD20 (+), CD79α (+), CyclinD1 (-), SOX11 (-), CD23 (-), CD38 (-), CD43 (-), BCL-2 (-), BCL-6 (-), Ki-67 (Li:5%) |
| 44 | Female | 53 | FL (grade 1/2) | *IGH-BCL2* | - | FL | Unclassified | FFPE | Lymph node | 0.34 | CD5 (-), CD10 (+), CD19 (+), CD20 (+), CD79α (+), CyclinD1 (-), SOX11 (-), CD23 (-), CD38 (-), CD43 (+), BCL-2 (+), BCL-6 (+), Ki-67 (Li:10%) |
| 45 | Female | 68 | NMZL | - | *NOTCH2* R2400* | MZL | MZL | FFPE | Lymph node | 0.55 | CD5 (-), CD10 (-), CD19 (+), CD20 (+), CD79α (+), CyclinD1 (-), SOX11 (-), CD23 (-), CD38 (-), CD43 (+), BCL-2 (+), BCL-6 (-), Ki-67 (Li:15%) |
| 46 | Female | 61 | FL (grade 1/2) | - | - | MZL | MZL | FFPE | Lymph node | 0.48 | CD5 (-), CD10 (+), CD19 (+), CD20 (+), CD79α (+), CyclinD1 (-), SOX11 (-), CD23 (-), CD38 (-), CD43 (+), BCL-2 (+), BCL-6 (+), Ki-67 (Li:30%) |
| 47 | Female | 25 | MALTL | - | - | MZL | MZL | FFPE | Parotid gland | 0.81 | CD5 (-), CD10 (-), CD19 (+), CD20 (+), CD79α (+), CyclinD1 (-), SOX11 (-), CD23 (-), CD38 (-), CD43 (-), BCL-2 (+), BCL-6 (-), Ki-67 (Li:5%) |
| 48 | Male | 58 | FL (grade 1/2) | *IGH-BCL2* | - | FL | Unclassified | FFPE | Kidney | 0.43 | CD5 (-), CD10 (-), CD19 (+), CD20 (+), CD79α (+), CyclinD1 (-), SOX11 (-), CD23 (+), CD38 (-), CD43 (-), BCL-2 (+), BCL-6 (+), Ki-67 (Li:50%) |
| 49 | Female | 53 | MALTL | *-* | - | MZL | MZL | FFPE | Palate | 0.71 | CD5 (-), CD10 (-), CD19 (+), CD20 (+), CD79α (+), CyclinD1 (-), SOX11 (-), CD23 (-), CD38 (-), CD43 (-), BCL-2 (+), BCL-6 (-), Ki-67 (Li:15%) |
| 50 | Female | 62 | MALTL | *-* | - | MZL | MZL | FFPE | Mediastinum | 0.72 | CD5 (-), CD10 (-), CD19 (+), CD20 (+), CD79α (+), CyclinD1 (-), SOX11 (-), CD23 (-), CD38 (-), CD43 (+), BCL-2 (+), BCL-6 (-), Ki-67 (Li:10%) |
| 51 | Female | 75 | MALTL | *-* | - | MZL | MZL | FFPE | Parotid gland | 0.46 | CD5 (-), CD10 (-), CD19 (+), CD20 (+), CD79α (+), CyclinD1 (-), SOX11 (-), CD23 (-), CD38 (+), BCL-2 (+), BCL-6 (-), Ki-67 (Li:20%) |
| 52 | Female | 41 | MALTL | *-* | - | MZL | Unclassified | FFPE | Parotid gland | 0.71 | CD5 (-), CD10 (-), CD19 (+), CD20 (+), CD79α (+), CyclinD1 (-), SOX11 (-), CD23 (-), CD38 (-), CD43 (+), BCL-2 (+), BCL-6 (-), Ki-67 (Li:3%) |
| 53 | Female | 47 | NMZL | *-* | - | MZL | MZL | FFPE | Lymph node | 0.84 | CD5 (-), CD10 (-), CD19 (+), CD20 (+), CD79α (+), CyclinD1 (-), SOX11 (-), CD23 (-), CD38 (-), CD43 (-), BCL-2 (+), BCL-6 (-), Ki-67 (Li:20%) |
| 54 | Male | 63 | CLL/SLL | *-* | - | CLL/SLL | CLL/SLL | FFPE | Lymph node | 0.73 | CD5 (+), CD10 (-), CD19 (+), CD20 (+), CD79α (+), CyclinD1 (-), SOX11 (-), CD23 (+), CD38 (-), CD43 (+), BCL-2 (+), BCL-6 (-), Ki-67 (Li:10%) |
| 55 | Male | 24 | MALTL | *-* | - | MZL | MZL | FFPE | Parotid gland | 0.56 | CD5 (-), CD10 (-), CD19 (+), CD20 (+), CD79α (+), CyclinD1 (-), SOX11 (-), CD23 (-), CD38 (-), CD43 (+), BCL-2 (+), BCL-6 (-), Ki-67 (Li:45%) |
| 56 | Male | 63 | cMCL | *IGH-CCND1* | - | cMCL | cMCL | FFPE | Waldeyer's ring | 0.69 | CD5 (+), CD10 (-), CD19 (+), CD20 (+), CD79α (+), CyclinD1 (+), SOX11 (+), CD38 (-), CD43 (+), BCL-2 (+), BCL-6 (-), Ki-67 (Li:25%) |
| 57 | Male | 65 | MALTL | *-* | - | MZL | Unclassified | FFPE | Ocular adnexa | 0.37 | CD5 (+), CD10 (-), CD19 (+), CD20 (+), CD79α (+), CyclinD1 (-), SOX11 (-), CD23 (-), CD38 (-), CD43 (+), BCL-2 (+), BCL-6 (-), Ki-67 (Li:20%) |
| 58 | Male | 69 | cMCL | *IGH-CCND1* | - | cMCL | cMCL | FFPE | Lymph node | 0.59 | CD5 (+), CD10 (-), CD19 (+), CD20 (+), CD79α (+), CyclinD1 (+), SOX11 (+), CD38 (+), CD43 (+), BCL-2 (+), BCL-6 (-), Ki-67 (Li:30%) |
| 59 | Male | 64 | FL (grade 1/2) | *IGH-BCL2* | - | FL | FL | FFPE | Lymph node | 0.71 | CD5 (-), CD10 (+), CD19 (+), CD20 (+), CD79α (+), CyclinD1 (-), SOX11 (-), CD23 (+), CD38 (-), CD43 (-), BCL-2 (+), BCL-6 (+), Ki-67 (Li:25%) |
| 60 | Male | 13 | FL (grade 1/2) | *-* | - | FL | Unclassified | FFPE | Lymph node | 0.59 | CD5 (-), CD10 (+), CD19 (+), CD20 (+), CD79α (+), CyclinD1 (-), SOX11 (-), CD38 (-),CD43 (-), BCL-2 (-), BCL-6 (+), Ki-67 (Li:75%) |
| 61 | Female | 64 | MALTL | *BIRC3-MALT1* | - | MZL | MZL | FFPE | Lung | 0.77 | CD5 (-), CD10 (-), CD19 (+), CD20 (+), CD79α (+), CyclinD1 (-), SOX11 (-), CD23 (-), CD38 (-), CD43 (-), BCL-2 (+), BCL-6 (-), Ki-67 (Li:10%) |
| 62 | Female | 51 | SMZL | *-* | *NOTCH2* R2400* | MZL | Unclassified | FFPE | Spleen | 0.66 | CD5 (-), CD10 (-), CD19 (+), CD20 (+), CD79α (+), CyclinD1 (-), SOX11 (-), CD23 (-), CD38 (-), CD43 (-), BCL-2 (-), BCL-6 (-) |
| 63 | Female | 40 | FL (grade 1/2) | *IGH-BCL2* | - | FL | Unclassified | FFPE | Mesentery | 0.34 | CD5 (-), CD10 (+), CD19 (+), CD20 (+), CD79α (+), CyclinD1 (-), SOX11 (-), CD23 (+), CD38 (-), CD43 (-), BCL-2 (+), Ki-67 (Li:15%) |
| 64 | Female | 52 | FL (grade 1/2) | *IGH-BCL2* | - | FL | FL | FFPE | Retroperitoneum mass | 0.63 | CD5 (-), CD10 (+), CD19 (+), CD20 (+), CD79α (+), CyclinD1 (-), SOX11 (-), CD43 (-), BCL-2 (+), BCL-6 (+), Ki-67 (Li:15%) |
| 65 | Female | 64 | FL (grade 1/2) | *IGH-BCL2* | - | FL | Unclassified | FFPE | Lymph node | 0.32 | CD5 (-), CD10 (-), CD19 (+), CD20 (+), CD79α (+), CyclinD1 (-), SOX11 (-), CD43 (-), BCL-2 (-), BCL-6 (-), Ki-67 (Li:70%) |
| 66 | Male | 16 | NMZL | *-* | - | MZL | Unclassified | FFPE | Lymph node | 0.45 | CD5 (-), CD10 (-), CD19 (+), CD20 (+), CD79α (+), CyclinD1 (-), SOX11 (-), CD23 (-), CD38 (-), CD43 (-), BCL-2 (+), BCL-6 (-), Ki-67 (Li:45%) |
| 67 | Male | 50 | NMZL | *-* | - | MZL | MZL | FFPE | Lymph node | 0.91 | CD5 (-), CD10 (-), CD19 (+), CD20 (+), CD79α (+), CyclinD1 (-), SOX11 (-), CD23 (-), CD38 (-), CD43 (-), BCL-2 (+), BCL-6 (-), Ki-67 (Li:10%) |
| 68 | Male | 35 | N/A | *-* | - | MZL | MZL | Fresh | Leukemic cell | 0.48 | N/A |
| 69 | Female | 52 | CLL/SLL | *-* | - | CLL/SLL | CLL/SLL | FFPE | Lymph node | 0.63 | CD5 (+), CD10 (-), CD19 (+), CD20 (+), CD79α (+), CyclinD1 (-), SOX11 (-), CD23 (+), CD38 (-), CD43 (+), BCL-2 (+), BCL-6 (+), Ki-67 (Li:20%) |
| 70 | Male | 65 | NMZL | *-* | *NOTCH2* R2400* | MZL | MZL | FFPE | Lymph node | 0.78 | CD5 (-), CD10 (-), CD19 (+), CD20 (+), CD79α (+), CyclinD1 (-), SOX11 (-), CD23 (+), CD38 (-), CD43 (-), BCL-2 (+), BCL-6 (-), Ki-67 (Li:20%) |
| 71 | Male | 57 | cMCL | *IGH-CCND1* | - | cMCL | cMCL | FFPE | Lymph node | 0.76 | CD5 (+), CD10 (-), CD19 (+), CD20 (+), CD79α (+), CyclinD1 (+), SOX11 (+), CD38 (-), CD43 (+), BCL-2 (+), BCL-6 (-), Ki-67 (Li:30%) |
| 72 | Female | 54 | FL (grade 1/2) | *IGH-BCL2* | - | FL | FL | FFPE | Lymph node | 0.7 | CD5 (-), CD10 (+), CD19 (+), CD20 (+), CD79α (+), CyclinD1 (-), SOX11 (-), CD23 (+), CD38 (-), CD43 (-), BCL-2 (+), BCL-6 (+), Ki-67 (Li:50%) |
| 73 | Male | 62 | FL (grade 1/2) | *IGH-BCL2* | *EZH2* Y646N | FL | FL | FFPE | Spleen | 0.61 | CD5 (-), CD10 (+), CD19 (+), CD20 (+), CD79α (+), CyclinD1 (-), SOX11 (-), CD23 (+), CD38 (-), CD43 (-), BCL-2 (+), BCL-6 (-), Ki-67 (Li:2%) |
| 74 | Male | 60 | CLL/SLL | +12 | - | CLL/SLL | CLL/SLL | FFPE | Lymph node | 0.67 | CD5 (+), CD10 (-), CD19 (+), CD20 (+), CD79α (+), CyclinD1 (-), SOX11 (-), CD23 (+), CD38 (-), CD43 (+), BCL-2 (+), BCL-6 (-), Ki-67 (Li:20%) |
| 75 | Female | 63 | FL (grade 1/2) | *IGH-BCL2* | - | FL | Unclassified | FFPE | Lymph node | 0.55 | CD5 (-), CD10 (-), CD19 (+), CD20 (+), CD79α (+), CyclinD1 (-), SOX11 (-), CD23 (+), CD38 (-), CD43 (+), BCL-2 (-), BCL-6 (+), Ki-67 (Li:60%) |
| 76 | Male | 67 | CLL/SLL | *-* | - | CLL/SLL | CLL/SLL | FFPE | Lymph node | 0.58 | CD5 (+), CD10 (-), CD19 (+), CD20 (+), CD79α (+), CyclinD1 (-), SOX11 (-), CD23 (+), CD38 (-), CD43 (+), BCL-2 (+), BCL-6 (-), Ki-67 (Li:15%) |
| 77 | Female | 52 | FL (grade 1/2) | *IGH-BCL2* | - | FL | Unclassified | FFPE | Lymph node | 0.56 | CD5 (-), CD10 (+), CD19 (+), CD20 (+), CD79α (+), CyclinD1 (-), SOX11 (-), CD23 (-), CD38 (-), CD43 (+), BCL-2 (+), BCL-6 (+), Ki-67 (Li:50%) |
| 78 | Female | 58 | MALTL | *-* | - | MZL | MZL | FFPE | Ocular adnexa | 0.66 | CD5 (-), CD10 (-), CD19 (+), CD20 (+), CD79α (+), CyclinD1 (-), SOX11 (-), CD23 (-), CD38 (-), CD43 (+), BCL-2 (+), BCL-6 (-), Ki-67 (Li:10%) |
| 79 | Male | 61 | MALTL | *BIRC3-MALT1* | - | MZL | MZL | FFPE | Intestinal mucosa | 0.61 | CD5 (-), CD10 (-), CD19 (+), CD20 (+), CD79α (+), CyclinD1 (-), SOX11 (-), CD23 (-), CD38 (-), CD43 (-), BCL-2 (+), BCL-6 (-), Ki-67 (Li:10%) |
| 80 | Female | 53 | FL (grade 1/2) | *IGH-BCL2* | - | FL | FL | FFPE | Lymph node | 0.84 | CD5 (-), CD10 (+), CD19 (+), CD20 (+), CD79α (+), CyclinD1 (-), SOX11 (-), CD23 (+), CD38 (-), CD43 (-), BCL-2 (+), BCL-6 (+), Ki-67 (Li:15%) |
| 81 | Female | 65 | MALTL | *-* | - | MZL | MZL | FFPE | Thyroid gland | 0.78 | CD5 (-), CD10 (-), CD19 (+), CD20 (+), CD79α (+), CyclinD1 (-), SOX11 (-), CD23 (-), CD38 (+), BCL-2 (-), BCL-6 (-), Ki-67 (Li:20%) |
| 82 | Male | 75 | MALTL | *BIRC3-MALT1* | - | MZL | MZL | FFPE | Lung | 0.76 | CD5 (-), CD10 (-), CD19 (+), CD20 (+), CD79α (+), CyclinD1 (-), SOX11 (-), CD23 (-), CD38 (-), CD43 (+), BCL-2 (+), BCL-6 (-), Ki-67 (Li:10%) |
| 83 | Male | 38 | cMCL | *IGH-CCND1* | - | cMCL | cMCL | FFPE | Lymph node | 0.93 | CD5 (+), CD10 (-), CD19 (+), CD20 (+), CD79α (+), CyclinD1 (+), SOX11 (+), CD38 (-), CD43 (+), BCL-2 (+), BCL-6 (-), Ki-67 (Li:60%) |
| 84 | Male | 55 | MALTL | *-* | - | MZL | MZL | FFPE | Ocular adnexa | 0.65 | CD5 (-), CD10 (-), CD19 (+), CD20 (+), CD79α (+), CyclinD1 (-), SOX11 (-), CD23 (-), CD38 (-), CD43 (-), BCL-2 (+), BCL-6 (-) |
| 85 | Female | 49 | NMZL | *-* | - | MZL | MZL | FFPE | Lymph node | 0.74 | CD5 (-), CD10 (-), CD19 (+), CD20 (+), CD79α (+), CyclinD1 (-), SOX11 (-), CD23 (-), CD38 (-), CD43 (-), BCL-2 (+), BCL-6 (-), Ki-67 (Li:15%) |
| 86 | Female | 61 | MALTL | *-* | - | MZL | Unclassified | FFPE | Palate | 0.78 | CD5 (-), CD10 (-), CD19 (+), CD20 (+), CD79α (+), CyclinD1 (-), SOX11 (-), CD23 (-), CD38 (+), CD43 (+), BCL-2 (+), BCL-6 (-), Ki-67 (Li:40%) |
| 87 | Female | 53 | MALTL | *-* | - | MZL | MZL | FFPE | Gastric mucosa | 0.73 | CD5 (-), CD10 (-), CD19 (+), CD20 (+), CD79α (+), CyclinD1 (-), SOX11 (-), CD23 (-), CD38 (-), CD43 (-), BCL-2 (+), BCL-6 (-), Ki-67 (Li:5%) |
| 88 | Male | 68 | FL (grade 1/2) | *IGH-BCL2* | - | FL | FL | FFPE | Lymph node | 0.74 | CD5 (-), CD10 (+), CD19 (+), CD20 (+), CD79α (+), CyclinD1 (-), SOX11 (-), CD23 (+), CD38 (-), CD43 (-), BCL-2 (-), BCL-6 (+), Ki-67 (Li:30%) |
| 89 | Female | 81 | FL (grade 1/2) | *IGH-BCL2* | - | FL | FL | FFPE | Parotid gland | 0.82 | CD5 (-), CD10 (+), CD19 (+), CD20 (+), CD79α (+), CyclinD1 (-), SOX11 (-), CD23 (+), CD38 (-), CD43 (-), BCL-2 (+), BCL-6 (+), Ki-67 (Li:30%) |
| 90 | Male | 53 | CLL/SLL | *-* | - | CLL/SLL | CLL/SLL | FFPE | Lymph node | 0.88 | CD5 (+), CD10 (-), CD19 (+), CD20 (+), CD79α (+), CyclinD1 (-), SOX11 (-), CD23 (+), CD38 (-), CD43 (+), BCL-2 (+), BCL-6 (-), Ki-67 (Li:20%) |
| 91 | Female | 61 | NMZL | *-* | - | MZL | MZL | FFPE | Lymph node | 0.81 | CD5 (-), CD10 (-), CD19 (+), CD20 (+), CD79α (+), CyclinD1 (-), SOX11 (-), CD23 (-), CD38 (-), CD43 (-), BCL-2 (+), BCL-6 (-), Ki-67 (Li:40%) |
| 92 | Male | 53 | CLL/SLL | *-* | - | CLL/SLL | CLL/SLL | FFPE | Waldeyer's ring | 0.41 | CD5 (+), CD10 (-), CD19 (+), CD20 (+), CD79α (+), CyclinD1 (-), SOX11 (-), CD23 (+), CD38 (-), CD43 (-), BCL-2 (+), BCL-6 (-), Ki-67 (Li:20%) |
| 93 | Male | 49 | NMZL | *-* | - | MZL | MZL | FFPE | Lymph node | 0.71 | CD5 (-), CD10 (-), CD19 (+), CD20 (+), CD79α (+), CyclinD1 (-), SOX11 (-), CD23 (+), CD38 (-), CD43 (-), BCL-2 (+), BCL-6 (-), Ki-67 (Li:10%) |
| 94 | Male | 60 | cMCL | *IGH-CCND1* | - | cMCL | cMCL | FFPE | Lymph node | 0.84 | CD5 (-), CD10 (-), CD19 (+), CD20 (+), CD79α (+), CyclinD1 (+), SOX11 (+), CD38 (-), CD43 (+), BCL-2 (+), BCL-6 (-), Ki-67 (Li:10%) |
| 95 | Male | 68 | cMCL | *IGH-CCND1* | - | cMCL | cMCL | FFPE | Spleen | 0.94 | CD5 (+), CD10 (-), CD19 (+), CD20 (+), CD79α (+), CyclinD1 (+), SOX11 (+), CD38 (-), CD43 (+), BCL-2 (+), BCL-6 (-), Ki-67 (Li:40%) |
| 96 | Male | 66 | MALTL | - | - | MZL | MZL | FFPE | Ocular adnexa | 0.88 | CD5 (-), CD10 (-), CD19 (+), CD20 (+), CD79α (+), CyclinD1 (-), SOX11 (-), CD23 (-), CD38 (-), CD43 (+), BCL-2 (+), BCL-6 (-), Ki-67 (Li:10%) |
| 97 | Male | 46 | CLL/SLL | +12 | - | CLL/SLL | CLL/SLL | FFPE | Lymph node | 0.86 | CD5 (+), CD10 (-), CD19 (+), CD20 (+), CD79α (+), CyclinD1 (-), SOX11 (-), CD23 (+), CD38 (-), CD43 (+), BCL-2 (+), BCL-6 (-), Ki-67 (Li:2%) |
| 98 | Female | 41 | MALTL | - | - | MZL | MZL | FFPE | Ocular adnexa | 0.73 | CD5 (-), CD10 (-), CD19 (+), CD20 (+), CD79α (+), CyclinD1 (-), SOX11 (-), CD23 (-), CD38 (-), CD43 (-), BCL-2 (+), BCL-6 (-), Ki-67 (Li:5%) |
| 99 | Female | 59 | MALTL | - | - | MZL | MZL | FFPE | Ocular adnexa | 0.91 | CD5 (-), CD10 (-), CD19 (+), CD20 (+), CD79α (+), CyclinD1 (-), SOX11 (-), CD23 (-), CD38 (-), CD43 (-), BCL-2 (+), BCL-6 (-), Ki-67 (Li:10%) |
| 100 | Female | 61 | MALTL | - | - | MZL | MZL | FFPE | Palate | 0.55 | CD5 (-), CD10 (-), CD19 (+), CD20 (+), CD79α (+), CyclinD1 (-), SOX11 (-), CD23 (-), CD38 (-), BCL-2 (+), BCL-6 (-) |
| 101 | Male | 47 | FL (grade 1/2) | *IGH-BCL2* | *EZH2* Y646H | FL | Unclassified | FFPE | Lymph node | 0.71 | CD5 (-), CD10 (+), CD19 (+), CD20 (+), CD79α (+), CyclinD1 (-), SOX11 (-), CD23 (+), CD38 (-), CD43 (-), BCL-2 (+), BCL-6 (+) |
| 102 | Male | 56 | N/A | *IGH-BCL2* | *EZH2* Y646H | FL | FL | Fresh | Leukemic cell | 0.91 | N/A |
| 103 | Female | 47 | N/A | *-* | - | MZL | MZL | Fresh | Leukemic cell | 0.68 | N/A |
| 104 | Female | 57 | N/A | *-* | - | Other SBCLN | Unclassified | Fresh | Leukemic cell | 0.64 | N/A |
| 105 | Female | 28 | N/A | *IGH-BCL2* | - | FL | FL | Fresh | Leukemic cell | 0.82 | N/A |
| 106 | Male | 49 | N/A | *IGH-CCND1* | - | cMCL | cMCL | Fresh | Hydrothorax cells | 0.56 | N/A |
| 107 | Male | 55 | N/A | +12 | - | CLL/SLL | CLL/SLL | Fresh | Leukemic cell | 0.72 | N/A |
| 108 | Male | 50 | FL (grade 1/2) | *IGH-BCL2* | - | FL | Unclassified | FFPE | Lymph node | 0.45 | CD5 (-), CD10 (+), CD19 (+), CD20 (+), CD79α (+), CyclinD1 (-), SOX11 (-), CD38 (-), BCL-2 (+), BCL-6 (+) |
| 109 | Male | 67 | cMCL | *IGH-CCND1* | - | cMCL | cMCL | FFPE | Gastric mucosa | 0.93 | CD5 (+), CD10 (-), CD19 (+), CD20 (+), CD79α (+), CyclinD1 (+), SOX11 (+), CD38 (-), CD43 (+), BCL-2 (+), BCL-6 (-), Ki-67 (Li:40%) |
| 110 | Male | 63 | tMALT | *-* | - | MZL | MZL | FFPE | Lymph node | 0.72 | CD5 (-), CD10 (-), CD19 (+), CD20 (+), CD79α (+), CyclinD1 (-), SOX11 (-), CD23 (-), CD38 (-), BCL-2 (+), BCL-6 (-), |
| 111 | Male | 70 | nnMCL | *IGH-CCND1* | - | nnMCL | nnMCL | Fresh | Bone marrow | 0.81 | CD5 (+), CD10 (-), CD19 (+), CD20 (+), CD79α (+), CyclinD1 (+), SOX11 (+), CD38 (-), CD43 (-), BCL-2 (+), BCL-6 (-), Ki-67 (Li:30%) |
| 112 | Male | 70 | cMCL | *IGH-CCND1* | - | cMCL | CLL/SLL | FFPE | Lymph node | 0.82 | CD5 (+), CD10 (-), CD19 (+), CD20 (+), CD79α (+), CyclinD1 (+), SOX11 (+), CD38 (-), CD43 (+), BCL-2 (+), BCL-6 (-), Ki-67 (Li:10%) |
| 113 | Male | 64 | cMCL | *IGH-CCND1* | - | cMCL | cMCL | FFPE | Lymph node | 0.76 | CD5 (+), CD10 (-), CD19 (+), CD20 (+), CD79α (+), CyclinD1 (+), SOX11 (+), CD38 (-), CD43 (+), BCL-2 (+), BCL-6 (-), Ki-67 (Li:10%) |
| 114 | Male | 66 | cMCL | *IGH-CCND1* | - | cMCL | cMCL | FFPE | Waldeyer's ring | 0.78 | CD5 (+), CD10 (-), CD19 (+), CD20 (+), CD79α (+), CyclinD1 (+), SOX11 (+), CD38 (-), CD43 (+), BCL-2 (+), BCL-6 (-), Ki-67 (Li:20%) |
| 115 | Female | 64 | nnMCL | *-* | - | nnMCL | Unclassified | FFPE | Lung | 0.66 | CD5 (+), CD10 (-), CD19 (+), CD20 (+), CD79α (+), CyclinD1 (+), SOX11 (-), CD38 (-), CD43 (+), BCL-2 (+), BCL-6 (-), Ki-67 (Li:0%) |
| 116 | Male | 63 | cMCL | *IGH-CCND1* | - | cMCL | cMCL | FFPE | Lymph node | 0.48 | CD5 (+), CD10 (-), CD19 (+), CD20 (+), CD79α (+), CyclinD1 (+), SOX11 (+), CD38 (-), CD43 (+), BCL-2 (+), BCL-6 (-), Ki-67 (Li:0%) |
| 117 | Female | 81 | FL (grade 1/2) | *IGH-BCL2* | - | FL | Unclassified | FFPE | Lymph node | 0.64 | CD5 (-), CD10 (+), CD19 (+), CD20 (+), CD79α (+), CyclinD1 (-), SOX11 (-), CD38 (-), BCL-2 (+), BCL-6 (+) |
| 118 | Female | 59 | FL (grade 1/2) | *IGH-BCL2* | *EZH2* Y646H | FL | FL | FFPE | Lymph node | 0.58 | CD5 (-), CD10 (+), CD19 (+), CD20 (+), CD79α (+), CyclinD1 (-), SOX11 (-), CD38 (-), BCL-2 (+), BCL-6 (+) |
| 119 | Male | 55 | N/A | *-* | *BRAF* V600E | Other SBCLN | Unclassified | Fresh | Leukemic cell | 0.41 | N/A |
| 120 | Female | 60 | MALTL | *-* | - | MZL | MZL | FFPE | Lymph node | 0.62 | CD5 (-), CD10 (-), CD19 (+), CD20 (+), CD79α (+), CyclinD1 (-), SOX11 (-), CD23 (-), CD38 (-), BCL-2 (+), BCL-6 (-) |
| 121 | Female | 50 | MALTL | *BIRC3-MALT1* | - | MZL | Unclassified | FFPE | Lung | 0.39 | CD5 (-), CD10 (-), CD19 (+), CD20 (+), CD79α (+), CyclinD1 (-), SOX11 (-), CD23 (-), CD38 (-), BCL-2 (+), BCL-6 (-) |
| 122 | Male | 75 | MALTL | *-* | *MYD88* L265P | MZL | MZL | FFPE | Gastric mucosa | 0.81 | CD5 (-), CD10 (-), CD19 (+), CD20 (+), CD79α (+), CyclinD1 (-), SOX11 (-), CD23 (-), CD38 (-), BCL-2 (+), BCL-6 (-) |
| 123 | Female | 75 | MALTL | *-* | *MYD88* L265P | MZL | MZL | FFPE | Lung | 0.73 | CD5 (-), CD10 (-), CD19 (+), CD20 (+), CD79α (+), CyclinD1 (-), SOX11 (-), CD23 (-), CD38 (-), BCL-2 (+), BCL-6 (-) |
| 124 | Female | 60 | MALTL | *-* | - | MZL | Unclassified | FFPE | Duodenum | 0.53 | CD5 (-), CD10 (-), CD19 (+), CD20 (+), CD79α (+), CyclinD1 (-), SOX11 (-), CD23 (-), CD38 (-), BCL-2 (+), BCL-6 (-) |
| 125 | Male | 73 | MALTL | *-* | - | MZL | Unclassified | FFPE | Gastric mucosa | 0.48 | CD5 (-), CD10 (-), CD19 (+), CD20 (+), CD79α (+), CyclinD1 (-), SOX11 (-), CD23 (-), CD38 (-), BCL-2 (+), BCL-6 (-) |
| 126 | Male | 73 | MALTL | *-* | - | MZL | Unclassified | FFPE | Gastric mucosa | 0.78 | CD5 (-), CD10 (-), CD19 (+), CD20 (+), CD79α (+), CyclinD1 (-), SOX11 (-), CD23 (-), CD38 (-), BCL-2 (+), BCL-6 (-) |
| 127 | Male | 64 | MALTL | *BIRC3-MALT1* | - | MZL | MZL | FFPE | Retroperitoneum mass | 0.57 | CD5 (-), CD10 (-), CD19 (+), CD20 (+), CD79α (+), CyclinD1 (-), SOX11 (-), CD23 (-), CD38 (-), CD43 (+), BCL-2 (+), BCL-6 (-), Ki-67 (Li:10%) |
| 128 | Female | 66 | MALTL | *-* | - | MZL | Unclassified | FFPE | Lung | 0.67 | CD5 (-), CD10 (-), CD19 (+), CD20 (+), CD79α (+), CyclinD1 (-), SOX11 (-), CD23 (-), CD38 (-), BCL-2 (+), BCL-6 (-) |
| 129 | Female | 70 | MALTL | *-* | - | MZL | MZL | FFPE | Subcutaneous tissue | 0.58 | CD5 (-), CD10 (-), CD19 (+), CD20 (+), CD79α (+), CyclinD1 (-), SOX11 (-), CD23 (-), CD38 (-), BCL-2 (+), BCL-6 (-) |
| 130 | Male | 77 | MALTL | *-* | - | MZL | MZL | FFPE | Lung | 0.75 | CD5 (-), CD10 (-), CD19 (+), CD20 (+), CD79α (+), CyclinD1 (-), SOX11 (-), CD23 (-), CD38 (-), BCL-2 (+), BCL-6 (-) |
| 131 | Male | 58 | MALTL | *-* | - | MZL | Unclassified | FFPE | Gastric mucosa | 0.52 | CD5 (-), CD10 (-), CD19 (+), CD20 (+), CD79α (+), CyclinD1 (-), SOX11 (-), CD23 (-), CD38 (-), BCL-2 (+), BCL-6 (-) |
| 132 | Female | 85 | MALTL | *-* | - | MZL | MZL | FFPE | Lung | 0.82 | CD5 (-), CD10 (-), CD19 (+), CD20 (+), CD79α (+), CyclinD1 (-), SOX11 (-), CD23 (-), CD38 (-), BCL-2 (+), BCL-6 (-) |
| 133 | Male | 49 | FL (grade 1/2) | *-* | - | MZL | MZL | FFPE | Lymph node | 0.37 | CD5 (-), CD10 (-), CD19 (+), CD20 (+), CD79α (+), CyclinD1 (-), SOX11 (-), CD23 (+), CD38 (-), CD43 (-), BCL-2 (+), BCL-6 (+) |
| 134 | Male | 57 | N/A | *-* | - | Other SBCLN | Unclassified | Fresh | Leukemic cell | 0.61 | N/A |
| 135 | Female | 36 | N/A | *-* | - | MZL | MZL | Fresh | Leukemic cell | 0.71 | N/A |
| 136 | Female | 62 | FL (grade 1/2) | *IGH-BCL2* | - | FL | FL | FFPE | Subcutaneous tissue | 0.68 | CD5 (-), CD10 (+), CD19 (+), CD20 (+), CD79α (+), CyclinD1 (-), SOX11 (-), CD38 (-), CD43 (-), BCL-2 (+), BCL-6 (+), Ki-67 (Li:15%) |
| 137 | Male | 40 | FL (grade 1/2) | *IGH-BCL2* | - | FL | FL | FFPE | Lymph node | 0.63 | CD5 (-), CD10 (+), CD19 (+), CD20 (+), CD79α (+), CyclinD1 (-), SOX11 (-), CD38 (-), CD43 (-), BCL-2 (+), BCL-6 (+), Ki-67 (Li:20%) |
| 138 | Male | 71 | nnMCL | *IGH-CCND1* | - | nnMCL | nnMCL | FFPE | Prostate | 0.78 | CD5 (+), CD10 (-), CD19 (+), CD20 (+), CD79α (+), CyclinD1 (+), SOX11 (-), CD38 (-), CD43 (+), BCL-2 (+), BCL-6 (-), Ki-67 (Li:0%) |
| 139 | Male | 62 | cMCL | *IGH-CCND1* | - | cMCL | cMCL | FFPE | Lymph node | 0.63 | CD5 (+), CD10 (-), CD19 (+), CD20 (+), CD79α (+), CyclinD1 (+), SOX11 (+), CD38 (-), CD43 (+), BCL-2 (+), BCL-6 (-), Ki-67 (Li:10%) |
| 140 | Male | 48 | cMCL | *IGH-CCND1* | - | cMCL | cMCL | FFPE | Parotid gland | 0.74 | CD5 (+), CD10 (-), CD19 (+), CD20 (+), CD79α (+), CyclinD1 (+), SOX11 (+), CD38 (-), CD43 (+), BCL-2 (+), BCL-6 (-), Ki-67 (Li:20%) |
| 141 | Male | 63 | cMCL | *IGH-CCND1* | - | cMCL | cMCL | FFPE | Lymph node | 0.94 | CD5 (+), CD10 (-), CD19 (+), CD20 (+), CD79α (+), CyclinD1 (+), SOX11 (+), CD38 (-), CD43 (+), BCL-2 (+), BCL-6 (-), Ki-67 (Li:20%) |
| 142 | Male | 52 | cMCL | *IGH-CCND1* | - | cMCL | cMCL | FFPE | Lymph node | 0.56 | CD5 (+), CD10 (-), CD19 (+), CD20 (+), CD79α (+), CyclinD1 (+), SOX11 (+), CD38 (-), CD43 (+), BCL-2 (+), BCL-6 (-), Ki-67 (Li:10%) |
| 143 | Female | 57 | cMCL | *IGH-CCND1* | - | cMCL | cMCL | FFPE | Waldeyer's ring | 0.53 | CD5 (+), CD10 (-), CD19 (+), CD20 (+), CD79α (+), CyclinD1 (+), SOX11 (+), CD38 (-), CD43 (+), BCL-2 (+), BCL-6 (-), Ki-67 (Li:90%) |
| 144 | Male | 54 | cMCL | *IGH-CCND1* | - | cMCL | cMCL | FFPE | Lymph node | 0.72 | CD5 (+), CD10 (-), CD19 (+), CD20 (+), CD79α (+), CyclinD1 (+), SOX11 (+), CD38 (-), CD43 (+), BCL-2 (+), BCL-6 (-), Ki-67 (Li:70%) |
| 145 | Male | 79 | cMCL | *IGH-CCND1* | - | cMCL | cMCL | FFPE | Lymph node | 0.81 | CD5 (+), CD10 (-), CD19 (+), CD20 (+), CD79α (+), CyclinD1 (+), SOX11 (+), CD38 (-), CD43 (+), BCL-2 (+), BCL-6 (-), Ki-67 (Li:40%) |
| 146 | Male | 83 | cMCL | *IGH-CCND1* | - | cMCL | cMCL | FFPE | Retroperitoneum mass | 0.82 | CD5 (+), CD10 (-), CD19 (+), CD20 (+), CD79α (+), CyclinD1 (+), SOX11 (+), CD38 (-), CD43 (+), BCL-2 (+), BCL-6 (-), Ki-67 (Li:50%) |
| 147 | Female | 60 | cMCL | *IGH-CCND1* | - | cMCL | cMCL | FFPE | Lymph node | 0.48 | CD5 (+), CD10 (-), CD19 (+), CD20 (+), CD79α (+), CyclinD1 (+), SOX11 (+), CD38 (-), CD43 (+), BCL-2 (+), BCL-6 (-), Ki-67 (Li:100%) |
| 148 | Male | 51 | cMCL | *IGH-CCND1* | - | cMCL | cMCL | FFPE | Lymph node | 0.8 | CD5 (+), CD10 (-), CD19 (+), CD20 (+), CD79α (+), CyclinD1 (+), SOX11 (+), CD38 (-), CD43 (+), BCL-2 (+), BCL-6 (-), Ki-67 (Li:80%) |
| 149 | Female | 56 | FL (grade 1/2) | *IGH-BCL2* | *EZH2* Y646N | FL | Unclassified | FFPE | Lymph node | 0.7 | CD5 (-), CD10 (+), CD19 (+), CD20 (+), CD79α (+), CyclinD1 (-), SOX11 (-), CD38 (-), BCL-2 (+), BCL-6 (+) |
| 150 | Female | 65 | FL (grade 1/2) | *IGH-BCL2* | - | FL | Unclassified | FFPE | Retroperitoneum mass | 0.68 | CD5 (-), CD10 (+), CD19 (+), CD20 (+), CD79α (+), CyclinD1 (-), SOX11 (-), CD38 (-), BCL-2 (-), BCL-6 (+) |
| 151 | Female | 68 | FL (grade 1/2) | *IGH-BCL2* | - | FL | FL | FFPE | Lymph node | 0.85 | CD5 (-), CD10 (+), CD19 (+), CD20 (+), CD79α (+), CyclinD1 (-), SOX11 (-), CD38 (-), BCL-2 (+), BCL-6 (-) |
| 152 | Male | 56 | FL (grade 1/2) | *IGH-BCL2* | - | FL | Unclassified | FFPE | Lymph node | 0.67 | CD5 (-), CD10 (+), CD19 (+), CD20 (+), CD79α (+), CyclinD1 (-), SOX11 (-), CD38 (-), BCL-2 (+), BCL-6 (+) |
| 153 | Male | 64 | FL (grade 1/2) | *IGH-BCL2* | - | FL | Unclassified | FFPE | Lymph node | 0.53 | CD5 (-), CD10 (+), CD19 (+), CD20 (+), CD79α (+), CyclinD1 (-), SOX11 (-), CD38 (-), BCL-2 (+), BCL-6 (+) |
| 154 | Male | 59 | FL (grade 1/2) | *IGH-BCL2* | - | FL | FL | FFPE | Lymph node | 0.88 | CD5 (-), CD10 (+), CD19 (+), CD20 (+), CD79α (+), CyclinD1 (-), SOX11 (-), CD38 (-), BCL-2 (+), BCL-6 (+) |
| 155 | Male | 39 | FL (grade 1/2) | *IGH-BCL2* | *EZH2* Y646N | FL | Unclassified | FFPE | Lymph node | 0.52 | CD5 (-), CD10 (+), CD19 (+), CD20 (+), CD79α (+), CyclinD1 (-), SOX11 (-), CD38 (-), BCL-2 (+), BCL-6 (+) |
| 156 | Female | 53 | FL (grade 1/2) | *IGH-BCL2* | - | FL | Unclassified | FFPE | Lymph node | 0.68 | CD5 (-), CD10 (+), CD19 (+), CD20 (+), CD79α (+), CyclinD1 (-), SOX11 (-), CD38 (-), BCL-2 (-), BCL-6 (+) |
| 157 | Male | 79 | FL (grade 1/2) | *IGH-BCL2* | - | FL | Unclassified | FFPE | Lymph node | 0.62 | CD5 (-), CD10 (+), CD19 (+), CD20 (+), CD79α (+), CyclinD1 (-), SOX11 (-), CD38 (-), BCL-2 (+), BCL-6 (+) |
| 158 | Female | 70 | FL (grade 1/2) | *IGH-BCL2* | - | FL | Unclassified | FFPE | Lymph node | 0.67 | CD5 (-), CD10 (+), CD19 (+), CD20 (+), CD79α (+), CyclinD1 (-), SOX11 (-), CD38 (-), BCL-2 (+), BCL-6 (+) |
| 159 | Male | 68 | FL (grade 1/2) | *IGH-BCL2* | - | FL | FL | FFPE | Lymph node | 0.89 | CD5 (-), CD10 (+), CD19 (+), CD20 (+), CD79α (+), CyclinD1 (-), SOX11 (-), CD38 (-), BCL-2 (+), BCL-6 (+ |
| 160 | Male | 75 | FL (grade 1/2) | *IGH-BCL2* | - | FL | Unclassified | FFPE | Lymph node | 0.39 | CD5 (-), CD10 (+), CD19 (+), CD20 (+), CD79α (+), CyclinD1 (-), SOX11 (-), CD23 (+), CD38 (-), CD43 (-), BCL-2 (+), BCL-6 (+) |
| 161 | Female | 69 | FL (grade 1/2) | *IGH-BCL2* | - | FL | Unclassified | FFPE | Parotid gland | 0.71 | CD5 (-), CD10 (+), CD19 (+), CD20 (+), CD79α (+), CyclinD1 (-), SOX11 (-), CD38 (-), BCL-2 (+), BCL-6 (+) |
| 162 | Male | 70 | FL (grade 1/2) | *IGH-BCL2* | - | FL | Unclassified | FFPE | Lymph node | 0.73 | CD5 (-), CD10 (+), CD19 (+), CD20 (+), CD79α (+), CyclinD1 (-), SOX11 (-), CD38 (-), BCL-2 (+), BCL-6 (+) |
| 163 | Male | 64 | FL (grade 1/2) | *IGH-BCL2* | - | FL | FL | FFPE | Lymph node | 0.61 | CD5 (-), CD10 (+), CD19 (+), CD20 (+), CD79α (+), CyclinD1 (-), SOX11 (-), CD38 (-), BCL-2 (+), BCL-6 (+) |
| 164 | Male | 57 | FL (grade 1/2) | *IGH-BCL2* | - | FL | Unclassified | FFPE | Lymph node | 0.62 | CD5 (-), CD10 (+), CD19 (+), CD20 (+), CD79α (+), CyclinD1 (-), SOX11 (-), CD38 (-), BCL-2 (+), BCL-6 (+) |
| 165 | Female | 60 | FL (grade 1/2) | *IGH-BCL2* | - | FL | FL | FFPE | Lymph node | 0.82 | CD5 (-), CD10 (+), CD19 (+), CD20 (+), CD79α (+), CyclinD1 (-), SOX11 (-), CD38 (-), BCL-2 (+), BCL-6 (+) |
| 166 | Male | 69 | FL (grade 1/2) | *IGH-BCL2* | - | FL | FL | FFPE | Lymph node | 0.72 | CD5 (-), CD10 (+), CD19 (+), CD20 (+), CD79α (+), CyclinD1 (-), SOX11 (-), CD38 (-), BCL-2 (+), BCL-6 (+) |
| 167 | Female | 62 | FL (grade 1/2) | *IGH-BCL2* | *EZH2* Y646S | FL | Unclassified | FFPE | Lymph node | 0.57 | CD5 (-), CD10 (+), CD19 (+), CD20 (+), CD79α (+), CyclinD1 (-), SOX11 (-), CD38 (-), BCL-2 (+), BCL-6 (+) |
| 168 | Male | 57 | FL (grade 1/2) | *IGH-BCL2* | - | FL | Unclassified | FFPE | Lymph node | 0.36 | CD5 (-), CD10 (+), CD19 (+), CD20 (+), CD79α (+), CyclinD1 (-), SOX11 (-), CD38 (-), BCL-2 (+), BCL-6 (-) |
| 169 | Male | 68 | FL (grade 1/2) | *-* | - | FL | Unclassified | FFPE | Lymph node | 0.63 | CD5 (-), CD10 (+), CD19 (+), CD20 (+), CD79α (+), CyclinD1 (-), SOX11 (-), CD38 (-), BCL-2 (+), BCL-6 (+) |
| 170 | Male | 54 | FL (grade 1/2) | *IGH-BCL2* | - | FL | Unclassified | FFPE | Lymph node | 0.56 | CD5 (-), CD10 (+), CD19 (+), CD20 (+), CD79α (+), CyclinD1 (-), SOX11 (-), CD38 (-), BCL-2 (+), BCL-6 (+) |
| 171 | Male | 62 | N/A | - | - | CLL/SLL | CLL/SLL | Fresh | Leukemic cell | 0.93 | N/A |
| 172 | Male | 37 | FL (grade 1/2) | *IGH-BCL2* | - | FL | FL | FFPE | Lymph node | 0.76 | CD5 (-), CD10 (+), CD19 (+), CD20 (+), CD79α (+), CyclinD1 (-), SOX11 (-), CD38 (-), BCL-2 (+), BCL-6 (+) |
| 173 | Female | 71 | N/A | - | - | MZL | MZL | Fresh | Leukemic cell | 0.81 | N/A |
| 174 | Male | 73 | FL (grade 3) | - | *EZH2* Y646N | FL | FL | FFPE | Lymph node | 0.84 | CD5 (-), CD10 (+), CD19 (+), CD20 (+), CD79α (+), CyclinD1 (-), SOX11 (-), CD38 (-), BCL-2 (+), BCL-6 (+) |
| 175 | Male | 73 | FL (grade 3) | *IGH-BCL2* | - | FL | FL | FFPE | Lymph node | 0.67 | CD5 (-), CD10 (+), CD19 (+), CD20 (+), CD79α (+), CyclinD1 (-), SOX11 (-), CD38 (-), BCL-2 (+), BCL-6 (+) |
| 176 | Male | 59 | FL (grade 3) | - | - | FL | FL | FFPE | Retroperitoneum mass | 0.83 | CD5 (-), CD10 (+), CD19 (+), CD20 (+), CD79α (+), CyclinD1 (-), SOX11 (-), CD38 (-), BCL-2 (+), BCL-6 (+) |
| 177 | Male | 61 | LPL/WM | - | *MYD88* L265P | LPL/WM | LPL/WM | Fresh | Bone marrow | 0.82 | CD5 (-), CD10 (-), CD19 (+), CD20 (+), CD79α (+), CyclinD1 (-), SOX11 (-), CD23 (-), CD38 (+), BCL-2 (-) |
| 178 | Male | 62 | LPL/WM | - | *MYD88* L265P | LPL/WM | LPL/WM | Fresh | Bone marrow | 0.58 | CD5 (-), CD10 (-), CD19 (+), CD20 (+), CD79α (+), CyclinD1 (-), SOX11 (-), CD23 (-), CD38 (+), BCL-2 (-) |
| 179 | Male | 85 | LPL/WM | - | *MYD88* L265P; *CXCR4* S335fs | LPL/WM | LPL/WM | Fresh | Bone marrow | 0.76 | CD5 (-), CD10 (-), CD19 (+), CD20 (+), CD79α (+), CyclinD1 (-), SOX11 (-), CD23 (-), CD38 (+), BCL-2 (-) |
| 180 | Female | 56 | LPL/WM | - | *MYD88* L265P | LPL/WM | Unclassified | Fresh | Bone marrow | 0.44 | CD5 (-), CD10 (-), CD19 (+), CD20 (+), CD79α (+), CyclinD1 (-), SOX11 (-), CD23 (-), CD38 (+), BCL-2 (-) |
| 181 | Male | 57 | LPL/WM | - | *MYD88* L265P | LPL/WM | Unclassified | Fresh | Bone marrow | 0.41 | CD5 (-), CD10 (-), CD19 (+), CD20 (+), CD79α (+), CyclinD1 (-), SOX11 (-), CD23 (-), CD38 (+), BCL-2 (-) |
| 182 | Male | 55 | N/A | - | *MYD88* L265P | LPL/WM | LPL/WM | Fresh | Leukemic cell | 0.42 | N/A |
| 183 | Male | 53 | N/A | - | *MYD88* L265P | LPL/WM | LPL/WM | Fresh | Leukemic cell | 0.71 | N/A |
| 184 | Male | 83 | N/A | - | *MYD88* L265P; *CXCR4* S338* | LPL/WM | LPL/WM | Fresh | Leukemic cell | 0.85 | N/A |
| 185 | Male | 66 | LPL/WM | - | *MYD88* L265P | LPL/WM | Unclassified | Fresh | Bone marrow | 0.51 | CD5 (-), CD10 (-), CD19 (+), CD20 (+), CD79α (+), CyclinD1 (-), SOX11 (-), CD23 (-), CD38 (+), BCL-2 (-) |
| 186 | Male | 62 | LPL/WM | - | *MYD88* L265P | LPL/WM | Unclassified | Fresh | Bone marrow | 0.55 | CD5 (-), CD10 (-), CD19 (+), CD20 (+), CD79α (+), CyclinD1 (-), SOX11 (-), CD23 (-), CD38 (+), BCL-2 (-) |
| 187 | Female | 66 | LPL/WM | - | *MYD88* L265P | LPL/WM | Unclassified | Fresh | Bone marrow | 0.54 | CD5 (-), CD10 (-), CD19 (+), CD20 (+), CD79α (+), CyclinD1 (-), SOX11 (-), CD23 (-), CD38 (+), BCL-2 (-) |
| 188 | Male | 61 | LPL/WM | - | *MYD88* L265P | LPL/WM | Unclassified | Fresh | Bone marrow | 0.38 | CD5 (-), CD10 (-), CD19 (+), CD20 (+), CD79α (+), CyclinD1 (-), SOX11 (-), CD23 (-), CD38 (+), BCL-2 (-) |
| 189 | Female | 56 | LPL/WM | - | *MYD88* L265P | LPL/WM | LPL/WM | Fresh | Bone marrow | 0.63 | CD5 (-), CD10 (-), CD19 (+), CD20 (+), CD79α (+), CyclinD1 (-), SOX11 (-), CD23 (-), CD38 (+), BCL-2 (-) |
| 190 | Male | 73 | LPL/WM | - | *MYD88* L265P | LPL/WM | Unclassified | Fresh | Bone marrow | 0.57 | CD5 (-), CD10 (-), CD19 (+), CD20 (+), CD79α (+), CyclinD1 (-), SOX11 (-), CD23 (-), CD38 (+), BCL-2 (-) |
| 191 | Male | 60 | LPL/WM | - | *MYD88* L265P; *CXCR4* S338* | LPL/WM | MZL | Fresh | Bone marrow | 0.66 | CD5 (-), CD10 (-), CD19 (+), CD20 (+), CD79α (+), CyclinD1 (-), SOX11 (-), CD23 (-), CD38 (+), BCL-2 (-) |
| 192 | Male | 63 | N/A | - | - | MZL | MZL | Fresh | Leukemic cell | 0.71 | N/A |
| 193 | Male | 53 | CLL/SLL | - | - | CLL/SLL | CLL/SLL | FFPE | Lymph node | 0.81 | CD5 (+), CD10 (-), CD19 (+), CD20 (+), CD79α (+), CyclinD1 (-), SOX11 (-), CD23 (+), CD38 (-), CD43 (+), BCL-2 (+), BCL-6 (-), Ki-67 (Li:20%) |
| 194 | Male | 44 | FL (grade 1/2) | *IGH-BCL2* | EZH2 Y646S | FL | FL | FFPE | Lymph node | 0.73 | CD5 (-), CD10 (+), CD19 (+), CD20 (+), CD79α (+), CyclinD1 (-), SOX11 (-), CD38 (-), BCL-2 (+), BCL-6 (+) |
| 195 | Female | 76 | N/A | - | *MYD88* L265P | LPL/WM | Unclassified | Fresh | Leukemic cell | 0.61 | N/A |
| 196 | Male | 65 | N/A | - | *BRAF* V600E | Other SBCLN | Unclassified | Fresh | Leukemic cell | 0.53 | N/A |
| 197 | Female | 57 | N/A | - | *MYD88* L265P; *CXCR4* S337fs | LPL/WM | Unclassified | Fresh | Leukemic cell | 0.38 | N/A |

Abbreviations: CLL/SLL, chronic lymphocytic leukemia/small lymphocytic lymphoma; cMCL, conventional mantle cell lymphoma; MCL, mantle cell lymphoma; nnMCL, leukemic non-nodal mantle cell lymphoma; FL, follicular lymphoma; MZL, marginal zone lymphoma; SMZL, splenic marginal zone lymphoma; MALTL, extranodal marginal zone lymphoma of mucosa-associated lymphoid tissue; NMZL, nodal marginal zone lymphoma; LPL/WM, lymphoplasmacytic lymphoma/Waldenström’s macroglobulinemia; FFPE, formalin-fixed paraffin-embedded.

**Supplementary Table S5. Distinct chromosomal aberrations and somatic mutations for ancillary diagnosis of SBCLN in our study**

| **Subtype** | **Distinct genetic aberration** | **CLL/SLL** | **FL** | **cMCL** | **nnMCL** | **NMZL** | **SMZL** | **MALT** | **LPL/WM** | **HCL** | **Cases that detected** |  |
| --- | --- | --- | --- | --- | --- | --- | --- | --- | --- | --- | --- | --- |
|  |  |  |  |  |  |  |  |  |  |  |  |  |
| **Translocation** | *BCL2* | R | C/S | R | R | R | R | R | R | R | All cases |  |
|  | *CCND1* | R | R | C/S | C/S | R | R | R | R | R | All cases |  |
|  | *MALT1* | R | R | R | R | R | R | C/S | R | R | All cases |  |
|  | *CCND2/CCND3* | R | R | C/S | C/S | R | R | R | R | R | If necessary |  |
|  | *IGH* (other partner) | I | R | R | R | I | I | I | R | R | If necessary |  |
| **Copy number** | +12 | C/S | R | I | I | R | R | R | R | R | All cases |  |
|  | del(7q) | R | R | R | R | R | C/S | R | R | R | All cases |  |
|  | del(17p) | C/S | R | C/S | C/S | R | I | R | R | R | If necessary |  |
|  | del(11q) | C/S | R | C/S | R | R | I | R | R | R | If necessary |  |
|  | del(13q) | C/S | R | C/S | C/S | R | I | R | R | R | If necessary |  |
| **Mutation** | *EZH2* exon18 (Y646) | R | C/S | R | R | R | R | R | R | R | All cases |  |
|  | *MYD88* exon5 (L265P) | I | R | R | R | I | R | I | C/S | R | All cases |  |
|  | *BRAF* exon15 (V600E) | R | R | R | R | I | R | R | R | C/S | All cases |  |
|  | *CXCR4* exon2 (S338X) | R | R | R | R | I | R | R | C/S | R | All cases |  |
|  | *NOTCH2* exon34 (R2400X) | R | R | I | I | C/S | C/S | I | R | R | All cases |  |
|  | *STAT6* exon12 (D419) | R | C/S | R | R | R | R | R | R | R | If necessary |  |
|  | *XPO1* exon15 (E517K) | I | R | R | R | R | R | R | R | R | If necessary |  |
|  | *SF3B1* exon15 (K700E) | I | R | R | R | R | R | R | R | R | If necessary |  |
|  | *NOTCH1* exon34 (P2514fs) | I | R | R | R | R | R | R | R | R | If necessary |  |
|  | *CREBBP* exon26 (R1446) | R | C/S | R | R | I | R | I | R | R | If necessary |  |
|  | ATP6V1B2 exon12 (R400Q) | R | C/S | R | R | R | R | R | R | R | If necessary |  |
|  | *ATM* exon63 (R3008) | I | R | I | R | R | R | R | R | R | If necessary |  |
|  | *MAP2K1* exon2 (F53) | R | R | R | R | I | R | R | R | R | If necessary |  |

Abbreviations: CLL/SLL, chronic lymphocytic leukemia/small lymphocytic lymphoma; cMCL, conventional mantle cell lymphoma; MCL, mantle cell lymphoma; nnMCL, leukemic non-nodal mantle cell lymphoma; FL, follicular lymphoma; MZL, marginal zone lymphoma; SMZL, splenic marginal zone lymphoma; MALTL, extranodal marginal zone lymphoma of mucosa-associated lymphoid tissue; NMZL, nodal marginal zone lymphoma; LPL/WM, lymphoplasmacytic lymphoma/Waldenström’s macroglobulinemia.

* The frequency data refers to numerous published literatures and public database, which was also only for reference

C/S: Common/Specific; I: Infrequent; R: rare or not seen in

**Supplementary Table S6. Gene list in targeted next-generation sequencing study**

| **Gene list** |
| --- |
| *ABL1, ACTG1, AKT1, ARID1A, ATM, ATP6AP1, ATP6V1B2, B2M, BCL10, BCL11B, BCL2, BCL6, BCOR, BIRC3, BRAF, BTG1, BTG2, BTK, CARD11, CASP10, CCND1, CCND3, CCR4, CCR7, CD28, CD58, CD70, CD79A, CD79B, CD83, CDKN1B, CDKN2A, CHD2, CNOT3, CREBBP, CRLF2, CTNNB1, CXCR4, DDX3X, DIS3, DNM2, DNMT3A, DTX1, DUSP2, EBF1, EED, EGR1, EGR2, EIF2A, EP300, ETV6, EZH2, FAM46C, FAS, FAT1, FBXW7, FGFR3, FLT3, FOXO1, FYN, GATA3, GNA13, GNAQ, GPR183, HIF1A, HIST1H1B, HIST1H1C, HIST1H1D, HIST1H1E, HNRNPA2B1, HRAS, HVCN1, ID3, IDH1, IDH2, IGLL5, IKBKB, IKZF1, IKZF3, IL7R, IRF4, ITPKB, JAK1, JAK2, JAK3, KDM6A, KIT, KLF2, KLHL6, KMT2C, KMT2D, KRAS, LTB, MAP2K1, MAP3K14, MAPK1, MAX, MED12, MEF2B, MYC, MYD88, NF1, NFE2, NFKBIE, NOTCH1, NOTCH2, NRAS, NT5C2, PAX5, PHF6, PIK3CA, PIK3R1, PIM1, PLCG1, PLCG2, POT1, POU2AF1, POU2F2, PRDM1, PRKCB, PTEN, PTPN1, PTPN11, RB1, RHOA, RPL10, RPS15, RRAGC, SAMHD1, SETD2, SF3B1, SGK1, SH2B3, SMARCA4, SMARCB1, SOCS1, STAT3, STAT5B, STAT6, TBL1XR1, TCF3, TET1, TET2, TMSB4X, TNFAIP3, TNFRSF14, TNFRSF1B, TP53, TRAF3, TRRAP, U2AF1, USP7, VAV1, VMA21, WHSC1, WT1, XPO1* |

**Supplementary Table S7. GEO datasets investigated in this study**

| **GEO Accession** | **Tumor purification** | **Subtype** | **CLL/SLL** | **FL** | **MCL** | **Other SBCLN** | **LPL/WM** | **MZL** | **Control** |  |
| --- | --- | --- | --- | --- | --- | --- | --- | --- | --- | --- |
|  |  |  |  |  |  |  |  |  |  |  |
|  |  | **No. of cases** | 343 | 227 | 119 | 17 | 31 | 71 | 231 |  |
| 50006 | Yes/No | 218 | 41/147 | 0 | 0 | 0 | 0 | 0 | 30 |  |
| 79196 | Yes | 159 | 54 | 12 | 54 | 12 | 4 | 23 | 0 |  |
| 53820 | No | 75 | 0 | 75 | 0 | 0 | 0 | 0 | 0 |  |
| 55267 | No | 69 | 0 | 63 | 0 | 0 | 0 | 0 | 6 |  |
| 56311 | Yes | 68 | 0 | 35 | 0 | 0 | 0 | 0 | 33 |  |
| 21029 | Yes | 62 | 62 | 0 | 0 | 0 | 0 | 0 | 0 |  |
| 16455 | Yes | 55 | 17 | 7 | 22 | 5 | 0 | 4 | 0 |  |
| 36000 | Yes | 38 | 0 | 0 | 38 | 0 | 0 | 0 | 0 |  |
| 27928 | - | 35 | 0 | 0 | 0 | 0 | 0 | 0 | 35 |  |
| 39577 | No | 32 | 0 | 2 | 0 | 0 | 0 | 30 | 0 |  |
| 7307 | - | 29 | 0 | 0 | 0 | 0 | 0 | 0 | 29 |  |
| 3526 | - | 27 | 0 | 0 | 0 | 0 | 0 | 0 | 27 |  |
| 9656 | Yes | 27 | 0 | 0 | 0 | 0 | 27 | 0 | 0 |  |
| 11504 | - | 25 | 0 | 0 | 0 | 0 | 0 | 0 | 25 |  |
| 65135 | No | 24 | 0 | 14 | 0 | 0 | 0 | 0 | 10 |  |
| 21554 | No | 24 | 0 | 5 | 5 | 0 | 0 | 14 | 0 |  |
| 29605 | Yes | 22 | 22 | 0 | 0 | 0 | 0 | 0 | 0 |  |
| 66384 | - | 14 | 0 | 0 | 0 | 0 | 0 | 0 | 14 |  |
| 86613 | No | 9 | 0 | 9 | 0 | 0 | 0 | 0 | 0 |  |
| 99316 | - | 6 | 0 | 0 | 0 | 0 | 0 | 0 | 6 |  |
| 12453 | Yes | 5 | 0 | 5 | 0 | 0 | 0 | 0 | 0 |  |
| 26725 | - | 5 | 0 | 0 | 0 | 0 | 0 | 0 | 5 |  |
| 18674 | - | 3 | 0 | 0 | 0 | 0 | 0 | 0 | 3 |  |
| 33846 | - | 3 | 0 | 0 | 0 | 0 | 0 | 0 | 3 |  |
| 57520 | - | 3 | 0 | 0 | 0 | 0 | 0 | 0 | 3 |  |
| 18490 | - | 1 | 0 | 0 | 0 | 0 | 0 | 0 | 1 |  |
| 7788 | - | 1 | 0 | 0 | 0 | 0 | 0 | 0 | 1 |  |

Abbreviations: CLL/SLL, chronic lymphocytic leukemia/small lymphocytic lymphoma; MCL, mantle cell lymphoma; FL, follicular lymphoma; MZL, marginal zone lymphoma; LPL/WM, lymphoplasmacytic lymphoma/Waldenström’s macroglobulinemia.

**Supplementary Table S8. Details of 1039 samples in GEP study**

| **ID** | **Entity** | **Diagnosis** | **Purification** | **GEO Accession** | **GSM** | **Microarray** | **Order of unsupervised hierarchical clustering based on pan genomic gene expression profiling (Figure 1, from left to right)** | **Order of unsupervised hierarchical clustering based on targeted gene expression signature (figure 1, from left to right)** |
| --- | --- | --- | --- | --- | --- | --- | --- | --- |
| 1 | CLL/SLL | CLL/SLL | Purified | GSE21029 | GSM525340 | Affymetrix U133 plus 2.0 | 431 | 430 |
| 2 | CLL/SLL | CLL/SLL | Purified | GSE21029 | GSM525341 | Affymetrix U133 plus 2.0 | 407 | 466 |
| 3 | CLL/SLL | CLL/SLL | Purified | GSE21029 | GSM525342 | Affymetrix U133 plus 2.0 | 425 | 496 |
| 4 | CLL/SLL | CLL/SLL | Purified | GSE21029 | GSM525343 | Affymetrix U133 plus 2.0 | 380 | 473 |
| 5 | CLL/SLL | CLL/SLL | Purified | GSE21029 | GSM525344 | Affymetrix U133 plus 2.0 | 396 | 455 |
| 6 | CLL/SLL | CLL/SLL | Purified | GSE21029 | GSM525345 | Affymetrix U133 plus 2.0 | 428 | 433 |
| 7 | CLL/SLL | CLL/SLL | Purified | GSE21029 | GSM525346 | Affymetrix U133 plus 2.0 | 401 | 441 |
| 8 | CLL/SLL | CLL/SLL | Purified | GSE21029 | GSM525347 | Affymetrix U133 plus 2.0 | 421 | 498 |
| 9 | CLL/SLL | CLL/SLL | Purified | GSE21029 | GSM525348 | Affymetrix U133 plus 2.0 | 404 | 749 |
| 10 | CLL/SLL | CLL/SLL | Purified | GSE21029 | GSM525349 | Affymetrix U133 plus 2.0 | 439 | 426 |
| 11 | CLL/SLL | CLL/SLL | Purified | GSE21029 | GSM525350 | Affymetrix U133 plus 2.0 | 419 | 484 |
| 12 | CLL/SLL | CLL/SLL | Purified | GSE21029 | GSM525351 | Affymetrix U133 plus 2.0 | 417 | 503 |
| 13 | CLL/SLL | CLL/SLL | Purified | GSE21029 | GSM525352 | Affymetrix U133 plus 2.0 | 415 | 501 |
| 14 | CLL/SLL | CLL/SLL | Purified | GSE21029 | GSM525353 | Affymetrix U133 plus 2.0 | 409 | 448 |
| 15 | CLL/SLL | CLL/SLL | Purified | GSE21029 | GSM525354 | Affymetrix U133 plus 2.0 | 393 | 459 |
| 16 | CLL/SLL | CLL/SLL | Purified | GSE21029 | GSM525355 | Affymetrix U133 plus 2.0 | 391 | 463 |
| 17 | CLL/SLL | CLL/SLL | Purified | GSE21029 | GSM525356 | Affymetrix U133 plus 2.0 | 436 | 438 |
| 18 | CLL/SLL | CLL/SLL | Purified | GSE21029 | GSM525357 | Affymetrix U133 plus 2.0 | 389 | 469 |
| 19 | CLL/SLL | CLL/SLL | Purified | GSE21029 | GSM525358 | Affymetrix U133 plus 2.0 | 434 | 436 |
| 20 | CLL/SLL | CLL/SLL | Purified | GSE29605 | GSM733547 | Affymetrix U133 plus 2.0 | 332 | 557 |
| 21 | CLL/SLL | CLL/SLL | Purified | GSE29605 | GSM733548 | Affymetrix U133 plus 2.0 | 330 | 551 |
| 22 | CLL/SLL | CLL/SLL | Purified | GSE29605 | GSM733549 | Affymetrix U133 plus 2.0 | 346 | 554 |
| 23 | CLL/SLL | CLL/SLL | Purified | GSE29605 | GSM733550 | Affymetrix U133 plus 2.0 | 347 | 478 |
| 24 | CLL/SLL | CLL/SLL | Purified | GSE29605 | GSM733551 | Affymetrix U133 plus 2.0 | 342 | 555 |
| 25 | CLL/SLL | CLL/SLL | Purified | GSE29605 | GSM733552 | Affymetrix U133 plus 2.0 | 333 | 558 |
| 26 | CLL/SLL | CLL/SLL | Purified | GSE29605 | GSM733553 | Affymetrix U133 plus 2.0 | 348 | 553 |
| 27 | CLL/SLL | CLL/SLL | Purified | GSE29605 | GSM733554 | Affymetrix U133 plus 2.0 | 328 | 552 |
| 28 | CLL/SLL | CLL/SLL | Purified | GSE29605 | GSM733555 | Affymetrix U133 plus 2.0 | 344 | 452 |
| 29 | CLL/SLL | CLL/SLL | Purified | GSE29605 | GSM733556 | Affymetrix U133 plus 2.0 | 349 | 547 |
| 30 | CLL/SLL | CLL/SLL | Purified | GSE29605 | GSM733557 | Affymetrix U133 plus 2.0 | 329 | 805 |
| 31 | CLL/SLL | CLL/SLL | Purified | GSE29605 | GSM733558 | Affymetrix U133 plus 2.0 | 345 | 581 |
| 32 | CLL/SLL | CLL/SLL | Purified | GSE29605 | GSM733559 | Affymetrix U133 plus 2.0 | 331 | 444 |
| 33 | CLL/SLL | CLL/SLL | Purified | GSE29605 | GSM733560 | Affymetrix U133 plus 2.0 | 337 | 445 |
| 34 | CLL/SLL | CLL/SLL | Purified | GSE29605 | GSM733561 | Affymetrix U133 plus 2.0 | 343 | 556 |
| 35 | CLL/SLL | CLL/SLL | Purified | GSE29605 | GSM733562 | Affymetrix U133 plus 2.0 | 338 | 486 |
| 36 | CLL/SLL | CLL/SLL | Purified | GSE29605 | GSM733563 | Affymetrix U133 plus 2.0 | 335 | 483 |
| 37 | CLL/SLL | CLL/SLL | Purified | GSE29605 | GSM733564 | Affymetrix U133 plus 2.0 | 334 | 808 |
| 38 | CLL/SLL | CLL/SLL | Purified | GSE29605 | GSM733565 | Affymetrix U133 plus 2.0 | 340 | 797 |
| 39 | CLL/SLL | CLL/SLL | Purified | GSE29605 | GSM733566 | Affymetrix U133 plus 2.0 | 339 | 443 |
| 40 | CLL/SLL | CLL/SLL | Purified | GSE29605 | GSM733567 | Affymetrix U133 plus 2.0 | 341 | 807 |
| 41 | CLL/SLL | CLL/SLL | Purified | GSE29605 | GSM733568 | Affymetrix U133 plus 2.0 | 336 | 491 |
| 42 | CLL/SLL | CLL/SLL | Purified | GSE16455 | GSM413634 | Affymetrix U133 plus 2.0 | 448 | 577 |
| 43 | CLL/SLL | CLL/SLL | Purified | GSE16455 | GSM413635 | Affymetrix U133 plus 2.0 | 443 | 480 |
| 44 | CLL/SLL | CLL/SLL | Purified | GSE16455 | GSM413636 | Affymetrix U133 plus 2.0 | 455 | 600 |
| 45 | CLL/SLL | CLL/SLL | Purified | GSE16455 | GSM413637 | Affymetrix U133 plus 2.0 | 454 | 567 |
| 46 | CLL/SLL | CLL/SLL | Purified | GSE16455 | GSM413638 | Affymetrix U133 plus 2.0 | 456 | 568 |
| 47 | CLL/SLL | CLL/SLL | Purified | GSE16455 | GSM413639 | Affymetrix U133 plus 2.0 | 452 | 574 |
| 48 | CLL/SLL | CLL/SLL | Purified | GSE16455 | GSM413640 | Affymetrix U133 plus 2.0 | 457 | 575 |
| 49 | CLL/SLL | CLL/SLL | Purified | GSE16455 | GSM413641 | Affymetrix U133 plus 2.0 | 453 | 571 |
| 50 | CLL/SLL | CLL/SLL | Purified | GSE16455 | GSM413642 | Affymetrix U133 plus 2.0 | 449 | 582 |
| 51 | CLL/SLL | CLL/SLL | Purified | GSE16455 | GSM413643 | Affymetrix U133 plus 2.0 | 451 | 573 |
| 52 | CLL/SLL | CLL/SLL | Purified | GSE16455 | GSM413644 | Affymetrix U133 plus 2.0 | 450 | 803 |
| 53 | CLL/SLL | CLL/SLL | Purified | GSE16455 | GSM413645 | Affymetrix U133 plus 2.0 | 447 | 576 |
| 54 | CLL/SLL | CLL/SLL | Purified | GSE16455 | GSM413646 | Affymetrix U133 plus 2.0 | 442 | 566 |
| 55 | CLL/SLL | CLL/SLL | Purified | GSE16455 | GSM413647 | Affymetrix U133 plus 2.0 | 444 | 493 |
| 56 | CLL/SLL | CLL/SLL | Purified | GSE16455 | GSM413648 | Affymetrix U133 plus 2.0 | 445 | 494 |
| 57 | CLL/SLL | CLL/SLL | Purified | GSE16455 | GSM413649 | Affymetrix U133 plus 2.0 | 446 | 481 |
| 58 | CLL/SLL | CLL/SLL | Purified | GSE16455 | GSM413650 | Affymetrix U133 plus 2.0 | 458 | 564 |
| 59 | CLL/SLL | CLL/SLL | Purified | GSE50006 | GSM1211903 | Affymetrix U133 plus 2.0 | 558 | 387 |
| 60 | CLL/SLL | CLL/SLL | Purified | GSE50006 | GSM1211904 | Affymetrix U133 plus 2.0 | 472 | 1013 |
| 61 | CLL/SLL | CLL/SLL | Purified | GSE50006 | GSM1211905 | Affymetrix U133 plus 2.0 | 557 | 341 |
| 62 | CLL/SLL | CLL/SLL | Purified | GSE50006 | GSM1211906 | Affymetrix U133 plus 2.0 | 556 | 405 |
| 63 | CLL/SLL | CLL/SLL | Purified | GSE50006 | GSM1211934 | Affymetrix U133 plus 2.0 | 350 | 589 |
| 64 | CLL/SLL | CLL/SLL | Purified | GSE50006 | GSM1211936 | Affymetrix U133 plus 2.0 | 374 | 599 |
| 65 | CLL/SLL | CLL/SLL | Purified | GSE50006 | GSM1211937 | Affymetrix U133 plus 2.0 | 359 | 594 |
| 66 | CLL/SLL | CLL/SLL | Purified | GSE50006 | GSM1211938 | Affymetrix U133 plus 2.0 | 354 | 804 |
| 67 | CLL/SLL | CLL/SLL | Purified | GSE50006 | GSM1211939 | Affymetrix U133 plus 2.0 | 375 | 508 |
| 68 | CLL/SLL | CLL/SLL | Purified | GSE50006 | GSM1211940 | Affymetrix U133 plus 2.0 | 376 | 518 |
| 69 | CLL/SLL | CLL/SLL | Purified | GSE50006 | GSM1211942 | Affymetrix U133 plus 2.0 | 364 | 516 |
| 70 | CLL/SLL | CLL/SLL | Purified | GSE50006 | GSM1211943 | Affymetrix U133 plus 2.0 | 355 | 583 |
| 71 | CLL/SLL | CLL/SLL | Purified | GSE50006 | GSM1211944 | Affymetrix U133 plus 2.0 | 372 | 505 |
| 72 | CLL/SLL | CLL/SLL | Purified | GSE50006 | GSM1211945 | Affymetrix U133 plus 2.0 | 360 | 595 |
| 73 | CLL/SLL | CLL/SLL | Purified | GSE50006 | GSM1211946 | Affymetrix U133 plus 2.0 | 365 | 509 |
| 74 | CLL/SLL | CLL/SLL | Purified | GSE50006 | GSM1211948 | Affymetrix U133 plus 2.0 | 358 | 479 |
| 75 | CLL/SLL | CLL/SLL | Purified | GSE50006 | GSM1211949 | Affymetrix U133 plus 2.0 | 373 | 506 |
| 76 | CLL/SLL | CLL/SLL | Purified | GSE50006 | GSM1211950 | Affymetrix U133 plus 2.0 | 325 | 511 |
| 77 | CLL/SLL | CLL/SLL | Purified | GSE50006 | GSM1211951 | Affymetrix U133 plus 2.0 | 157 | 781 |
| 78 | CLL/SLL | CLL/SLL | Purified | GSE50006 | GSM1211953 | Affymetrix U133 plus 2.0 | 363 | 510 |
| 79 | CLL/SLL | CLL/SLL | Purified | GSE50006 | GSM1211954 | Affymetrix U133 plus 2.0 | 371 | 512 |
| 80 | CLL/SLL | CLL/SLL | Purified | GSE50006 | GSM1211955 | Affymetrix U133 plus 2.0 | 377 | 519 |
| 81 | CLL/SLL | CLL/SLL | Purified | GSE50006 | GSM1211956 | Affymetrix U133 plus 2.0 | 378 | 588 |
| 82 | CLL/SLL | CLL/SLL | Purified | GSE50006 | GSM1211957 | Affymetrix U133 plus 2.0 | 352 | 453 |
| 83 | CLL/SLL | CLL/SLL | Purified | GSE50006 | GSM1211958 | Affymetrix U133 plus 2.0 | 326 | 824 |
| 84 | CLL/SLL | CLL/SLL | Purified | GSE50006 | GSM1211959 | Affymetrix U133 plus 2.0 | 137 | 569 |
| 85 | CLL/SLL | CLL/SLL | Purified | GSE50006 | GSM1211972 | Affymetrix U133 plus 2.0 | 138 | 607 |
| 86 | CLL/SLL | CLL/SLL | Purified | GSE50006 | GSM1211976 | Affymetrix U133 plus 2.0 | 356 | 507 |
| 87 | CLL/SLL | CLL/SLL | Purified | GSE50006 | GSM1211980 | Affymetrix U133 plus 2.0 | 353 | 477 |
| 88 | CLL/SLL | CLL/SLL | Purified | GSE50006 | GSM1211982 | Affymetrix U133 plus 2.0 | 361 | 517 |
| 89 | CLL/SLL | CLL/SLL | Purified | GSE50006 | GSM1211983 | Affymetrix U133 plus 2.0 | 357 | 492 |
| 90 | CLL/SLL | CLL/SLL | Purified | GSE50006 | GSM1211985 | Affymetrix U133 plus 2.0 | 362 | 514 |
| 91 | CLL/SLL | CLL/SLL | Purified | GSE50006 | GSM1211986 | Affymetrix U133 plus 2.0 | 369 | 515 |
| 92 | CLL/SLL | CLL/SLL | Purified | GSE50006 | GSM1211987 | Affymetrix U133 plus 2.0 | 370 | 522 |
| 93 | CLL/SLL | CLL/SLL | Purified | GSE50006 | GSM1211988 | Affymetrix U133 plus 2.0 | 351 | 590 |
| 94 | CLL/SLL | CLL/SLL | Purified | GSE50006 | GSM1211989 | Affymetrix U133 plus 2.0 | 368 | 521 |
| 95 | CLL/SLL | CLL/SLL | Purified | GSE50006 | GSM1211991 | Affymetrix U133 plus 2.0 | 366 | 513 |
| 96 | CLL/SLL | CLL/SLL | Purified | GSE50006 | GSM1211992 | Affymetrix U133 plus 2.0 | 323 | 782 |
| 97 | CLL/SLL | CLL/SLL | Purified | GSE50006 | GSM1211994 | Affymetrix U133 plus 2.0 | 367 | 523 |
| 98 | CLL/SLL | CLL/SLL | Purified | GSE50006 | GSM1211997 | Affymetrix U133 plus 2.0 | 116 | 520 |
| 99 | CLL/SLL | CLL/SLL | Purified | GSE50006 | GSM1212015 | Affymetrix U133 plus 2.0 | 136 | 549 |
| 100 | CLL/SLL | CLL/SLL | Purified | GSE79196 | GSM2087714 | Affymetrix U133 plus 2.0 | 106 | 488 |
| 101 | CLL/SLL | CLL/SLL | Purified | GSE79196 | GSM2087715 | Affymetrix U133 plus 2.0 | 110 | 604 |
| 102 | CLL/SLL | CLL/SLL | Purified | GSE79196 | GSM2087716 | Affymetrix U133 plus 2.0 | 123 | 585 |
| 103 | CLL/SLL | CLL/SLL | Purified | GSE79196 | GSM2087717 | Affymetrix U133 plus 2.0 | 84 | 528 |
| 104 | CLL/SLL | CLL/SLL | Purified | GSE79196 | GSM2087718 | Affymetrix U133 plus 2.0 | 118 | 586 |
| 105 | CLL/SLL | CLL/SLL | Purified | GSE79196 | GSM2087719 | Affymetrix U133 plus 2.0 | 124 | 559 |
| 106 | CLL/SLL | CLL/SLL | Purified | GSE79196 | GSM2087720 | Affymetrix U133 plus 2.0 | 92 | 539 |
| 107 | CLL/SLL | CLL/SLL | Purified | GSE79196 | GSM2087721 | Affymetrix U133 plus 2.0 | 104 | 572 |
| 108 | CLL/SLL | CLL/SLL | Purified | GSE79196 | GSM2087722 | Affymetrix U133 plus 2.0 | 130 | 596 |
| 109 | CLL/SLL | CLL/SLL | Purified | GSE79196 | GSM2087723 | Affymetrix U133 plus 2.0 | 108 | 489 |
| 110 | CLL/SLL | CLL/SLL | Purified | GSE79196 | GSM2087724 | Affymetrix U133 plus 2.0 | 101 | 606 |
| 111 | CLL/SLL | CLL/SLL | Purified | GSE79196 | GSM2087725 | Affymetrix U133 plus 2.0 | 94 | 533 |
| 112 | CLL/SLL | CLL/SLL | Purified | GSE79196 | GSM2087726 | Affymetrix U133 plus 2.0 | 97 | 537 |
| 113 | CLL/SLL | CLL/SLL | Purified | GSE79196 | GSM2087727 | Affymetrix U133 plus 2.0 | 113 | 800 |
| 114 | CLL/SLL | CLL/SLL | Purified | GSE79196 | GSM2087728 | Affymetrix U133 plus 2.0 | 102 | 602 |
| 115 | CLL/SLL | CLL/SLL | Purified | GSE79196 | GSM2087729 | Affymetrix U133 plus 2.0 | 126 | 548 |
| 116 | CLL/SLL | CLL/SLL | Purified | GSE79196 | GSM2087730 | Affymetrix U133 plus 2.0 | 132 | 562 |
| 117 | CLL/SLL | CLL/SLL | Purified | GSE79196 | GSM2087731 | Affymetrix U133 plus 2.0 | 114 | 801 |
| 118 | CLL/SLL | CLL/SLL | Purified | GSE79196 | GSM2087732 | Affymetrix U133 plus 2.0 | 134 | 561 |
| 119 | CLL/SLL | CLL/SLL | Purified | GSE79196 | GSM2087733 | Affymetrix U133 plus 2.0 | 81 | 541 |
| 120 | CLL/SLL | CLL/SLL | Purified | GSE79196 | GSM2087734 | Affymetrix U133 plus 2.0 | 119 | 587 |
| 121 | CLL/SLL | CLL/SLL | Purified | GSE79196 | GSM2087736 | Affymetrix U133 plus 2.0 | 88 | 540 |
| 122 | CLL/SLL | CLL/SLL | Purified | GSE79196 | GSM2087737 | Affymetrix U133 plus 2.0 | 109 | 490 |
| 123 | CLL/SLL | CLL/SLL | Purified | GSE79196 | GSM2087738 | Affymetrix U133 plus 2.0 | 121 | 605 |
| 124 | CLL/SLL | CLL/SLL | Purified | GSE79196 | GSM2087739 | Affymetrix U133 plus 2.0 | 125 | 584 |
| 125 | CLL/SLL | CLL/SLL | Purified | GSE79196 | GSM2087740 | Affymetrix U133 plus 2.0 | 82 | 535 |
| 126 | CLL/SLL | CLL/SLL | Purified | GSE79196 | GSM2087741 | Affymetrix U133 plus 2.0 | 86 | 543 |
| 127 | CLL/SLL | CLL/SLL | Purified | GSE79196 | GSM2087742 | Affymetrix U133 plus 2.0 | 112 | 802 |
| 128 | CLL/SLL | CLL/SLL | Purified | GSE79196 | GSM2087743 | Affymetrix U133 plus 2.0 | 135 | 531 |
| 129 | CLL/SLL | CLL/SLL | Purified | GSE79196 | GSM2087744 | Affymetrix U133 plus 2.0 | 85 | 529 |
| 130 | CLL/SLL | CLL/SLL | Purified | GSE79196 | GSM2087745 | Affymetrix U133 plus 2.0 | 122 | 592 |
| 131 | CLL/SLL | CLL/SLL | Purified | GSE79196 | GSM2087746 | Affymetrix U133 plus 2.0 | 95 | 532 |
| 132 | CLL/SLL | CLL/SLL | Purified | GSE79196 | GSM2087747 | Affymetrix U133 plus 2.0 | 107 | 482 |
| 133 | CLL/SLL | CLL/SLL | Purified | GSE79196 | GSM2087748 | Affymetrix U133 plus 2.0 | 98 | 536 |
| 134 | CLL/SLL | CLL/SLL | Purified | GSE79196 | GSM2087749 | Affymetrix U133 plus 2.0 | 127 | 597 |
| 135 | CLL/SLL | CLL/SLL | Purified | GSE79196 | GSM2087750 | Affymetrix U133 plus 2.0 | 83 | 542 |
| 136 | CLL/SLL | CLL/SLL | Purified | GSE79196 | GSM2087751 | Affymetrix U133 plus 2.0 | 89 | 545 |
| 137 | CLL/SLL | CLL/SLL | Purified | GSE79196 | GSM2087752 | Affymetrix U133 plus 2.0 | 128 | 598 |
| 138 | CLL/SLL | CLL/SLL | Purified | GSE79196 | GSM2087753 | Affymetrix U133 plus 2.0 | 117 | 565 |
| 139 | CLL/SLL | CLL/SLL | Purified | GSE79196 | GSM2087754 | Affymetrix U133 plus 2.0 | 96 | 538 |
| 140 | CLL/SLL | CLL/SLL | Purified | GSE79196 | GSM2087755 | Affymetrix U133 plus 2.0 | 93 | 560 |
| 141 | CLL/SLL | CLL/SLL | Purified | GSE79196 | GSM2087756 | Affymetrix U133 plus 2.0 | 103 | 603 |
| 142 | CLL/SLL | CLL/SLL | Purified | GSE79196 | GSM2087757 | Affymetrix U133 plus 2.0 | 90 | 546 |
| 143 | CLL/SLL | CLL/SLL | Purified | GSE79196 | GSM2087758 | Affymetrix U133 plus 2.0 | 87 | 530 |
| 144 | CLL/SLL | CLL/SLL | Purified | GSE79196 | GSM2087759 | Affymetrix U133 plus 2.0 | 133 | 563 |
| 145 | CLL/SLL | CLL/SLL | Purified | GSE79196 | GSM2087760 | Affymetrix U133 plus 2.0 | 111 | 608 |
| 146 | CLL/SLL | CLL/SLL | Purified | GSE79196 | GSM2087761 | Affymetrix U133 plus 2.0 | 131 | 534 |
| 147 | CLL/SLL | CLL/SLL | Purified | GSE79196 | GSM2087762 | Affymetrix U133 plus 2.0 | 105 | 487 |
| 148 | CLL/SLL | CLL/SLL | Purified | GSE79196 | GSM2087763 | Affymetrix U133 plus 2.0 | 120 | 593 |
| 149 | CLL/SLL | CLL/SLL | Purified | GSE79196 | GSM2087765 | Affymetrix U133 plus 2.0 | 99 | 601 |
| 150 | CLL/SLL | CLL/SLL | Purified | GSE79196 | GSM2087768 | Affymetrix U133 plus 2.0 | 100 | 591 |
| 151 | CLL/SLL | CLL/SLL | Purified | GSE79196 | GSM2087772 | Affymetrix U133 plus 2.0 | 115 | 809 |
| 152 | CLL/SLL | CLL/SLL | Purified | GSE79196 | GSM2087835 | Affymetrix U133 plus 2.0 | 282 | 806 |
| 153 | CLL/SLL | CLL/SLL | Purified | GSE79196 | GSM2087836 | Affymetrix U133 plus 2.0 | 91 | 544 |
| 154 | CLL/SLL | CLL/SLL | Purified | GSE21029 | GSM525359 | Affymetrix U133 plus 2.0 | 432 | 429 |
| 155 | CLL/SLL | CLL/SLL | Purified | GSE21029 | GSM525360 | Affymetrix U133 plus 2.0 | 406 | 465 |
| 156 | CLL/SLL | CLL/SLL | Purified | GSE21029 | GSM525361 | Affymetrix U133 plus 2.0 | 426 | 495 |
| 157 | CLL/SLL | CLL/SLL | Purified | GSE21029 | GSM525362 | Affymetrix U133 plus 2.0 | 382 | 471 |
| 158 | CLL/SLL | CLL/SLL | Purified | GSE21029 | GSM525363 | Affymetrix U133 plus 2.0 | 411 | 450 |
| 159 | CLL/SLL | CLL/SLL | Purified | GSE21029 | GSM525364 | Affymetrix U133 plus 2.0 | 398 | 475 |
| 160 | CLL/SLL | CLL/SLL | Purified | GSE21029 | GSM525365 | Affymetrix U133 plus 2.0 | 384 | 461 |
| 161 | CLL/SLL | CLL/SLL | Purified | GSE21029 | GSM525366 | Affymetrix U133 plus 2.0 | 395 | 454 |
| 162 | CLL/SLL | CLL/SLL | Purified | GSE21029 | GSM525367 | Affymetrix U133 plus 2.0 | 427 | 432 |
| 163 | CLL/SLL | CLL/SLL | Purified | GSE21029 | GSM525368 | Affymetrix U133 plus 2.0 | 402 | 442 |
| 164 | CLL/SLL | CLL/SLL | Purified | GSE21029 | GSM525369 | Affymetrix U133 plus 2.0 | 422 | 499 |
| 165 | CLL/SLL | CLL/SLL | Purified | GSE21029 | GSM525370 | Affymetrix U133 plus 2.0 | 403 | 748 |
| 166 | CLL/SLL | CLL/SLL | Purified | GSE21029 | GSM525371 | Affymetrix U133 plus 2.0 | 438 | 425 |
| 167 | CLL/SLL | CLL/SLL | Purified | GSE21029 | GSM525372 | Affymetrix U133 plus 2.0 | 386 | 457 |
| 168 | CLL/SLL | CLL/SLL | Purified | GSE21029 | GSM525373 | Affymetrix U133 plus 2.0 | 413 | 446 |
| 169 | CLL/SLL | CLL/SLL | Purified | GSE21029 | GSM525374 | Affymetrix U133 plus 2.0 | 388 | 468 |
| 170 | CLL/SLL | CLL/SLL | Purified | GSE21029 | GSM525375 | Affymetrix U133 plus 2.0 | 433 | 435 |
| 171 | CLL/SLL | CLL/SLL | Purified | GSE21029 | GSM525314 | Affymetrix U133 plus 2.0 | 430 | 431 |
| 172 | CLL/SLL | CLL/SLL | Purified | GSE21029 | GSM525315 | Affymetrix U133 plus 2.0 | 408 | 467 |
| 173 | CLL/SLL | CLL/SLL | Purified | GSE21029 | GSM525316 | Affymetrix U133 plus 2.0 | 424 | 497 |
| 174 | CLL/SLL | CLL/SLL | Purified | GSE21029 | GSM525317 | Affymetrix U133 plus 2.0 | 383 | 474 |
| 175 | CLL/SLL | CLL/SLL | Purified | GSE21029 | GSM525318 | Affymetrix U133 plus 2.0 | 381 | 472 |
| 176 | CLL/SLL | CLL/SLL | Purified | GSE21029 | GSM525319 | Affymetrix U133 plus 2.0 | 412 | 451 |
| 177 | CLL/SLL | CLL/SLL | Purified | GSE21029 | GSM525320 | Affymetrix U133 plus 2.0 | 399 | 476 |
| 178 | CLL/SLL | CLL/SLL | Purified | GSE21029 | GSM525321 | Affymetrix U133 plus 2.0 | 385 | 462 |
| 179 | CLL/SLL | CLL/SLL | Purified | GSE21029 | GSM525322 | Affymetrix U133 plus 2.0 | 397 | 456 |
| 180 | CLL/SLL | CLL/SLL | Purified | GSE21029 | GSM525323 | Affymetrix U133 plus 2.0 | 429 | 434 |
| 181 | CLL/SLL | CLL/SLL | Purified | GSE21029 | GSM525324 | Affymetrix U133 plus 2.0 | 400 | 440 |
| 182 | CLL/SLL | CLL/SLL | Purified | GSE21029 | GSM525325 | Affymetrix U133 plus 2.0 | 423 | 500 |
| 183 | CLL/SLL | CLL/SLL | Purified | GSE21029 | GSM525326 | Affymetrix U133 plus 2.0 | 405 | 750 |
| 184 | CLL/SLL | CLL/SLL | Purified | GSE21029 | GSM525327 | Affymetrix U133 plus 2.0 | 440 | 427 |
| 185 | CLL/SLL | CLL/SLL | Purified | GSE21029 | GSM525328 | Affymetrix U133 plus 2.0 | 441 | 428 |
| 186 | CLL/SLL | CLL/SLL | Purified | GSE21029 | GSM525329 | Affymetrix U133 plus 2.0 | 387 | 458 |
| 187 | CLL/SLL | CLL/SLL | Purified | GSE21029 | GSM525330 | Affymetrix U133 plus 2.0 | 420 | 485 |
| 188 | CLL/SLL | CLL/SLL | Purified | GSE21029 | GSM525331 | Affymetrix U133 plus 2.0 | 418 | 504 |
| 189 | CLL/SLL | CLL/SLL | Purified | GSE21029 | GSM525332 | Affymetrix U133 plus 2.0 | 416 | 502 |
| 190 | CLL/SLL | CLL/SLL | Purified | GSE21029 | GSM525333 | Affymetrix U133 plus 2.0 | 410 | 449 |
| 191 | CLL/SLL | CLL/SLL | Purified | GSE21029 | GSM525334 | Affymetrix U133 plus 2.0 | 394 | 460 |
| 192 | CLL/SLL | CLL/SLL | Purified | GSE21029 | GSM525335 | Affymetrix U133 plus 2.0 | 392 | 464 |
| 193 | CLL/SLL | CLL/SLL | Purified | GSE21029 | GSM525336 | Affymetrix U133 plus 2.0 | 437 | 439 |
| 194 | CLL/SLL | CLL/SLL | Purified | GSE21029 | GSM525337 | Affymetrix U133 plus 2.0 | 414 | 447 |
| 195 | CLL/SLL | CLL/SLL | Purified | GSE21029 | GSM525338 | Affymetrix U133 plus 2.0 | 390 | 470 |
| 196 | CLL/SLL | CLL/SLL | Purified | GSE21029 | GSM525339 | Affymetrix U133 plus 2.0 | 435 | 437 |
| 197 | CLL/SLL | CLL/SLL | Non-purified | GSE50006 | GSM1212022 | Affymetrix U133 plus 2.0 | 562 | 388 |
| 198 | CLL/SLL | CLL/SLL | Non-purified | GSE50006 | GSM1212023 | Affymetrix U133 plus 2.0 | 554 | 287 |
| 199 | CLL/SLL | CLL/SLL | Non-purified | GSE50006 | GSM1212024 | Affymetrix U133 plus 2.0 | 499 | 305 |
| 200 | CLL/SLL | CLL/SLL | Non-purified | GSE50006 | GSM1212025 | Affymetrix U133 plus 2.0 | 549 | 290 |
| 201 | CLL/SLL | CLL/SLL | Non-purified | GSE50006 | GSM1212027 | Affymetrix U133 plus 2.0 | 560 | 753 |
| 202 | CLL/SLL | CLL/SLL | Non-purified | GSE50006 | GSM1212028 | Affymetrix U133 plus 2.0 | 551 | 314 |
| 203 | CLL/SLL | CLL/SLL | Non-purified | GSE50006 | GSM1212029 | Affymetrix U133 plus 2.0 | 555 | 288 |
| 204 | CLL/SLL | CLL/SLL | Non-purified | GSE50006 | GSM1212030 | Affymetrix U133 plus 2.0 | 531 | 579 |
| 205 | CLL/SLL | CLL/SLL | Non-purified | GSE50006 | GSM1212031 | Affymetrix U133 plus 2.0 | 552 | 298 |
| 206 | CLL/SLL | CLL/SLL | Non-purified | GSE50006 | GSM1212032 | Affymetrix U133 plus 2.0 | 550 | 420 |
| 207 | CLL/SLL | CLL/SLL | Non-purified | GSE50006 | GSM1212033 | Affymetrix U133 plus 2.0 | 553 | 289 |
| 208 | CLL/SLL | CLL/SLL | Non-purified | GSE50006 | GSM1212034 | Affymetrix U133 plus 2.0 | 561 | 354 |
| 209 | CLL/SLL | CLL/SLL | Non-purified | GSE50006 | GSM1212035 | Affymetrix U133 plus 2.0 | 563 | 348 |
| 210 | CLL/SLL | CLL/SLL | Non-purified | GSE50006 | GSM1212036 | Affymetrix U133 plus 2.0 | 508 | 385 |
| 211 | CLL/SLL | CLL/SLL | Non-purified | GSE50006 | GSM1212037 | Affymetrix U133 plus 2.0 | 514 | 357 |
| 212 | CLL/SLL | CLL/SLL | Non-purified | GSE50006 | GSM1212038 | Affymetrix U133 plus 2.0 | 516 | 333 |
| 213 | CLL/SLL | CLL/SLL | Non-purified | GSE50006 | GSM1212039 | Affymetrix U133 plus 2.0 | 523 | 323 |
| 214 | CLL/SLL | CLL/SLL | Non-purified | GSE50006 | GSM1212040 | Affymetrix U133 plus 2.0 | 589 | 418 |
| 215 | CLL/SLL | CLL/SLL | Non-purified | GSE50006 | GSM1212041 | Affymetrix U133 plus 2.0 | 500 | 295 |
| 216 | CLL/SLL | CLL/SLL | Non-purified | GSE50006 | GSM1212042 | Affymetrix U133 plus 2.0 | 493 | 524 |
| 217 | CLL/SLL | CLL/SLL | Non-purified | GSE50006 | GSM1212043 | Affymetrix U133 plus 2.0 | 501 | 372 |
| 218 | CLL/SLL | CLL/SLL | Non-purified | GSE50006 | GSM1212044 | Affymetrix U133 plus 2.0 | 494 | 525 |
| 219 | CLL/SLL | CLL/SLL | Non-purified | GSE50006 | GSM1212045 | Affymetrix U133 plus 2.0 | 524 | 326 |
| 220 | CLL/SLL | CLL/SLL | Non-purified | GSE50006 | GSM1212046 | Affymetrix U133 plus 2.0 | 511 | 349 |
| 221 | CLL/SLL | CLL/SLL | Non-purified | GSE50006 | GSM1212047 | Affymetrix U133 plus 2.0 | 518 | 327 |
| 222 | CLL/SLL | CLL/SLL | Non-purified | GSE50006 | GSM1212048 | Affymetrix U133 plus 2.0 | 597 | 376 |
| 223 | CLL/SLL | CLL/SLL | Non-purified | GSE50006 | GSM1212049 | Affymetrix U133 plus 2.0 | 573 | 403 |
| 224 | CLL/SLL | CLL/SLL | Non-purified | GSE50006 | GSM1212050 | Affymetrix U133 plus 2.0 | 574 | 358 |
| 225 | CLL/SLL | CLL/SLL | Non-purified | GSE50006 | GSM1212051 | Affymetrix U133 plus 2.0 | 532 | 580 |
| 226 | CLL/SLL | CLL/SLL | Non-purified | GSE50006 | GSM1212052 | Affymetrix U133 plus 2.0 | 611 | 382 |
| 227 | CLL/SLL | CLL/SLL | Non-purified | GSE50006 | GSM1212053 | Affymetrix U133 plus 2.0 | 533 | 300 |
| 228 | CLL/SLL | CLL/SLL | Non-purified | GSE50006 | GSM1212054 | Affymetrix U133 plus 2.0 | 495 | 526 |
| 229 | CLL/SLL | CLL/SLL | Non-purified | GSE50006 | GSM1212055 | Affymetrix U133 plus 2.0 | 520 | 336 |
| 230 | CLL/SLL | CLL/SLL | Non-purified | GSE50006 | GSM1212056 | Affymetrix U133 plus 2.0 | 521 | 393 |
| 231 | CLL/SLL | CLL/SLL | Non-purified | GSE50006 | GSM1212057 | Affymetrix U133 plus 2.0 | 592 | 367 |
| 232 | CLL/SLL | CLL/SLL | Non-purified | GSE50006 | GSM1212058 | Affymetrix U133 plus 2.0 | 519 | 352 |
| 233 | CLL/SLL | CLL/SLL | Non-purified | GSE50006 | GSM1212059 | Affymetrix U133 plus 2.0 | 509 | 386 |
| 234 | CLL/SLL | CLL/SLL | Non-purified | GSE50006 | GSM1212060 | Affymetrix U133 plus 2.0 | 515 | 355 |
| 235 | CLL/SLL | CLL/SLL | Non-purified | GSE50006 | GSM1212061 | Affymetrix U133 plus 2.0 | 517 | 383 |
| 236 | CLL/SLL | CLL/SLL | Non-purified | GSE50006 | GSM1212062 | Affymetrix U133 plus 2.0 | 639 | 390 |
| 237 | CLL/SLL | CLL/SLL | Non-purified | GSE50006 | GSM1212063 | Affymetrix U133 plus 2.0 | 522 | 379 |
| 238 | CLL/SLL | CLL/SLL | Non-purified | GSE50006 | GSM1212064 | Affymetrix U133 plus 2.0 | 502 | 410 |
| 239 | CLL/SLL | CLL/SLL | Non-purified | GSE50006 | GSM1212065 | Affymetrix U133 plus 2.0 | 503 | 315 |
| 240 | CLL/SLL | CLL/SLL | Non-purified | GSE50006 | GSM1212066 | Affymetrix U133 plus 2.0 | 588 | 419 |
| 241 | CLL/SLL | CLL/SLL | Non-purified | GSE50006 | GSM1212067 | Affymetrix U133 plus 2.0 | 633 | 359 |
| 242 | CLL/SLL | CLL/SLL | Non-purified | GSE50006 | GSM1212068 | Affymetrix U133 plus 2.0 | 612 | 283 |
| 243 | CLL/SLL | CLL/SLL | Non-purified | GSE50006 | GSM1212069 | Affymetrix U133 plus 2.0 | 506 | 339 |
| 244 | CLL/SLL | CLL/SLL | Non-purified | GSE50006 | GSM1212070 | Affymetrix U133 plus 2.0 | 638 | 389 |
| 245 | CLL/SLL | CLL/SLL | Non-purified | GSE50006 | GSM1212071 | Affymetrix U133 plus 2.0 | 616 | 335 |
| 246 | CLL/SLL | CLL/SLL | Non-purified | GSE50006 | GSM1212072 | Affymetrix U133 plus 2.0 | 535 | 334 |
| 247 | CLL/SLL | CLL/SLL | Non-purified | GSE50006 | GSM1212073 | Affymetrix U133 plus 2.0 | 582 | 404 |
| 248 | CLL/SLL | CLL/SLL | Non-purified | GSE50006 | GSM1212074 | Affymetrix U133 plus 2.0 | 510 | 377 |
| 249 | CLL/SLL | CLL/SLL | Non-purified | GSE50006 | GSM1212075 | Affymetrix U133 plus 2.0 | 512 | 411 |
| 250 | CLL/SLL | CLL/SLL | Non-purified | GSE50006 | GSM1212076 | Affymetrix U133 plus 2.0 | 634 | 321 |
| 251 | CLL/SLL | CLL/SLL | Non-purified | GSE50006 | GSM1212077 | Affymetrix U133 plus 2.0 | 504 | 373 |
| 252 | CLL/SLL | CLL/SLL | Non-purified | GSE50006 | GSM1212078 | Affymetrix U133 plus 2.0 | 575 | 324 |
| 253 | CLL/SLL | CLL/SLL | Non-purified | GSE50006 | GSM1212079 | Affymetrix U133 plus 2.0 | 637 | 422 |
| 254 | CLL/SLL | CLL/SLL | Non-purified | GSE50006 | GSM1212080 | Affymetrix U133 plus 2.0 | 576 | 392 |
| 255 | CLL/SLL | CLL/SLL | Non-purified | GSE50006 | GSM1212081 | Affymetrix U133 plus 2.0 | 584 | 397 |
| 256 | CLL/SLL | CLL/SLL | Non-purified | GSE50006 | GSM1212082 | Affymetrix U133 plus 2.0 | 583 | 394 |
| 257 | CLL/SLL | CLL/SLL | Non-purified | GSE50006 | GSM1212083 | Affymetrix U133 plus 2.0 | 568 | 363 |
| 258 | CLL/SLL | CLL/SLL | Non-purified | GSE50006 | GSM1212084 | Affymetrix U133 plus 2.0 | 565 | 398 |
| 259 | CLL/SLL | CLL/SLL | Non-purified | GSE50006 | GSM1212085 | Affymetrix U133 plus 2.0 | 566 | 337 |
| 260 | CLL/SLL | CLL/SLL | Non-purified | GSE50006 | GSM1212086 | Affymetrix U133 plus 2.0 | 570 | 401 |
| 261 | CLL/SLL | CLL/SLL | Non-purified | GSE50006 | GSM1212087 | Affymetrix U133 plus 2.0 | 564 | 415 |
| 262 | CLL/SLL | CLL/SLL | Non-purified | GSE50006 | GSM1212088 | Affymetrix U133 plus 2.0 | 569 | 395 |
| 263 | CLL/SLL | CLL/SLL | Non-purified | GSE50006 | GSM1212089 | Affymetrix U133 plus 2.0 | 567 | 399 |
| 264 | CLL/SLL | CLL/SLL | Non-purified | GSE50006 | GSM1212090 | Affymetrix U133 plus 2.0 | 571 | 423 |
| 265 | CLL/SLL | CLL/SLL | Non-purified | GSE50006 | GSM1212092 | Affymetrix U133 plus 2.0 | 496 | 527 |
| 266 | CLL/SLL | CLL/SLL | Non-purified | GSE50006 | GSM1212093 | Affymetrix U133 plus 2.0 | 590 | 421 |
| 267 | CLL/SLL | CLL/SLL | Non-purified | GSE50006 | GSM1212094 | Affymetrix U133 plus 2.0 | 614 | 407 |
| 268 | CLL/SLL | CLL/SLL | Non-purified | GSE50006 | GSM1212095 | Affymetrix U133 plus 2.0 | 608 | 293 |
| 269 | CLL/SLL | CLL/SLL | Non-purified | GSE50006 | GSM1212096 | Affymetrix U133 plus 2.0 | 607 | 296 |
| 270 | CLL/SLL | CLL/SLL | Non-purified | GSE50006 | GSM1212097 | Affymetrix U133 plus 2.0 | 615 | 408 |
| 271 | CLL/SLL | CLL/SLL | Non-purified | GSE50006 | GSM1212098 | Affymetrix U133 plus 2.0 | 605 | 328 |
| 272 | CLL/SLL | CLL/SLL | Non-purified | GSE50006 | GSM1212099 | Affymetrix U133 plus 2.0 | 613 | 356 |
| 273 | CLL/SLL | CLL/SLL | Non-purified | GSE50006 | GSM1212100 | Affymetrix U133 plus 2.0 | 629 | 350 |
| 274 | CLL/SLL | CLL/SLL | Non-purified | GSE50006 | GSM1212101 | Affymetrix U133 plus 2.0 | 621 | 319 |
| 275 | CLL/SLL | CLL/SLL | Non-purified | GSE50006 | GSM1212102 | Affymetrix U133 plus 2.0 | 593 | 303 |
| 276 | CLL/SLL | CLL/SLL | Non-purified | GSE50006 | GSM1212103 | Affymetrix U133 plus 2.0 | 622 | 351 |
| 277 | CLL/SLL | CLL/SLL | Non-purified | GSE50006 | GSM1212104 | Affymetrix U133 plus 2.0 | 635 | 345 |
| 278 | CLL/SLL | CLL/SLL | Non-purified | GSE50006 | GSM1212105 | Affymetrix U133 plus 2.0 | 604 | 291 |
| 279 | CLL/SLL | CLL/SLL | Non-purified | GSE50006 | GSM1212106 | Affymetrix U133 plus 2.0 | 606 | 311 |
| 280 | CLL/SLL | CLL/SLL | Non-purified | GSE50006 | GSM1212107 | Affymetrix U133 plus 2.0 | 598 | 369 |
| 281 | CLL/SLL | CLL/SLL | Non-purified | GSE50006 | GSM1212108 | Affymetrix U133 plus 2.0 | 623 | 353 |
| 282 | CLL/SLL | CLL/SLL | Non-purified | GSE50006 | GSM1212109 | Affymetrix U133 plus 2.0 | 586 | 416 |
| 283 | CLL/SLL | CLL/SLL | Non-purified | GSE50006 | GSM1212110 | Affymetrix U133 plus 2.0 | 599 | 304 |
| 284 | CLL/SLL | CLL/SLL | Non-purified | GSE50006 | GSM1212111 | Affymetrix U133 plus 2.0 | 580 | 342 |
| 285 | CLL/SLL | CLL/SLL | Non-purified | GSE50006 | GSM1212112 | Affymetrix U133 plus 2.0 | 578 | 307 |
| 286 | CLL/SLL | CLL/SLL | Non-purified | GSE50006 | GSM1212113 | Affymetrix U133 plus 2.0 | 579 | 364 |
| 287 | CLL/SLL | CLL/SLL | Non-purified | GSE50006 | GSM1212114 | Affymetrix U133 plus 2.0 | 624 | 365 |
| 288 | CLL/SLL | CLL/SLL | Non-purified | GSE50006 | GSM1212115 | Affymetrix U133 plus 2.0 | 618 | 320 |
| 289 | CLL/SLL | CLL/SLL | Non-purified | GSE50006 | GSM1212116 | Affymetrix U133 plus 2.0 | 577 | 424 |
| 290 | CLL/SLL | CLL/SLL | Non-purified | GSE50006 | GSM1212117 | Affymetrix U133 plus 2.0 | 594 | 325 |
| 291 | CLL/SLL | CLL/SLL | Non-purified | GSE50006 | GSM1212118 | Affymetrix U133 plus 2.0 | 619 | 378 |
| 292 | CLL/SLL | CLL/SLL | Non-purified | GSE50006 | GSM1212119 | Affymetrix U133 plus 2.0 | 617 | 406 |
| 293 | CLL/SLL | CLL/SLL | Non-purified | GSE50006 | GSM1212120 | Affymetrix U133 plus 2.0 | 620 | 366 |
| 294 | CLL/SLL | CLL/SLL | Non-purified | GSE50006 | GSM1212121 | Affymetrix U133 plus 2.0 | 640 | 316 |
| 295 | CLL/SLL | CLL/SLL | Non-purified | GSE50006 | GSM1212122 | Affymetrix U133 plus 2.0 | 585 | 346 |
| 296 | CLL/SLL | CLL/SLL | Non-purified | GSE50006 | GSM1212123 | Affymetrix U133 plus 2.0 | 626 | 361 |
| 297 | CLL/SLL | CLL/SLL | Non-purified | GSE50006 | GSM1212124 | Affymetrix U133 plus 2.0 | 627 | 360 |
| 298 | CLL/SLL | CLL/SLL | Non-purified | GSE50006 | GSM1212125 | Affymetrix U133 plus 2.0 | 602 | 308 |
| 299 | CLL/SLL | CLL/SLL | Non-purified | GSE50006 | GSM1212126 | Affymetrix U133 plus 2.0 | 572 | 396 |
| 300 | CLL/SLL | CLL/SLL | Non-purified | GSE50006 | GSM1212127 | Affymetrix U133 plus 2.0 | 636 | 344 |
| 301 | CLL/SLL | CLL/SLL | Non-purified | GSE50006 | GSM1212128 | Affymetrix U133 plus 2.0 | 600 | 292 |
| 302 | CLL/SLL | CLL/SLL | Non-purified | GSE50006 | GSM1212129 | Affymetrix U133 plus 2.0 | 505 | 362 |
| 303 | CLL/SLL | CLL/SLL | Non-purified | GSE50006 | GSM1212130 | Affymetrix U133 plus 2.0 | 603 | 285 |
| 304 | CLL/SLL | CLL/SLL | Non-purified | GSE50006 | GSM1212131 | Affymetrix U133 plus 2.0 | 631 | 402 |
| 305 | CLL/SLL | CLL/SLL | Non-purified | GSE50006 | GSM1212132 | Affymetrix U133 plus 2.0 | 581 | 343 |
| 306 | CLL/SLL | CLL/SLL | Non-purified | GSE50006 | GSM1212133 | Affymetrix U133 plus 2.0 | 601 | 286 |
| 307 | CLL/SLL | CLL/SLL | Non-purified | GSE50006 | GSM1212134 | Affymetrix U133 plus 2.0 | 625 | 318 |
| 308 | CLL/SLL | CLL/SLL | Non-purified | GSE50006 | GSM1212135 | Affymetrix U133 plus 2.0 | 609 | 294 |
| 309 | CLL/SLL | CLL/SLL | Non-purified | GSE50006 | GSM1212136 | Affymetrix U133 plus 2.0 | 507 | 340 |
| 310 | CLL/SLL | CLL/SLL | Non-purified | GSE50006 | GSM1212137 | Affymetrix U133 plus 2.0 | 632 | 409 |
| 311 | CLL/SLL | CLL/SLL | Non-purified | GSE50006 | GSM1212138 | Affymetrix U133 plus 2.0 | 628 | 371 |
| 312 | CLL/SLL | CLL/SLL | Non-purified | GSE50006 | GSM1212139 | Affymetrix U133 plus 2.0 | 610 | 317 |
| 313 | CLL/SLL | CLL/SLL | Non-purified | GSE50006 | GSM1212140 | Affymetrix U133 plus 2.0 | 630 | 322 |
| 314 | CLL/SLL | CLL/SLL | Non-purified | GSE50006 | GSM1212141 | Affymetrix U133 plus 2.0 | 641 | 391 |
| 315 | CLL/SLL | CLL/SLL | Non-purified | GSE50006 | GSM1212145 | Affymetrix U133 plus 2.0 | 528 | 338 |
| 316 | CLL/SLL | CLL/SLL | Non-purified | GSE50006 | GSM1212146 | Affymetrix U133 plus 2.0 | 529 | 301 |
| 317 | CLL/SLL | CLL/SLL | Non-purified | GSE50006 | GSM1212147 | Affymetrix U133 plus 2.0 | 471 | 297 |
| 318 | CLL/SLL | CLL/SLL | Non-purified | GSE50006 | GSM1212148 | Affymetrix U133 plus 2.0 | 591 | 284 |
| 319 | CLL/SLL | CLL/SLL | Non-purified | GSE50006 | GSM1212149 | Affymetrix U133 plus 2.0 | 587 | 417 |
| 320 | CLL/SLL | CLL/SLL | Non-purified | GSE50006 | GSM1212150 | Affymetrix U133 plus 2.0 | 530 | 384 |
| 321 | CLL/SLL | CLL/SLL | Non-purified | GSE50006 | GSM1212151 | Affymetrix U133 plus 2.0 | 497 | 306 |
| 322 | CLL/SLL | CLL/SLL | Non-purified | GSE50006 | GSM1212152 | Affymetrix U133 plus 2.0 | 534 | 578 |
| 323 | CLL/SLL | CLL/SLL | Non-purified | GSE50006 | GSM1212153 | Affymetrix U133 plus 2.0 | 542 | 330 |
| 324 | CLL/SLL | CLL/SLL | Non-purified | GSE50006 | GSM1212154 | Affymetrix U133 plus 2.0 | 498 | 302 |
| 325 | CLL/SLL | CLL/SLL | Non-purified | GSE50006 | GSM1212155 | Affymetrix U133 plus 2.0 | 536 | 368 |
| 326 | CLL/SLL | CLL/SLL | Non-purified | GSE50006 | GSM1212156 | Affymetrix U133 plus 2.0 | 537 | 374 |
| 327 | CLL/SLL | CLL/SLL | Non-purified | GSE50006 | GSM1212157 | Affymetrix U133 plus 2.0 | 547 | 313 |
| 328 | CLL/SLL | CLL/SLL | Non-purified | GSE50006 | GSM1212158 | Affymetrix U133 plus 2.0 | 595 | 299 |
| 329 | CLL/SLL | CLL/SLL | Non-purified | GSE50006 | GSM1212159 | Affymetrix U133 plus 2.0 | 525 | 380 |
| 330 | CLL/SLL | CLL/SLL | Non-purified | GSE50006 | GSM1212160 | Affymetrix U133 plus 2.0 | 527 | 332 |
| 331 | CLL/SLL | CLL/SLL | Non-purified | GSE50006 | GSM1212161 | Affymetrix U133 plus 2.0 | 513 | 347 |
| 332 | CLL/SLL | CLL/SLL | Non-purified | GSE50006 | GSM1212162 | Affymetrix U133 plus 2.0 | 539 | 413 |
| 333 | CLL/SLL | CLL/SLL | Non-purified | GSE50006 | GSM1212163 | Affymetrix U133 plus 2.0 | 543 | 331 |
| 334 | CLL/SLL | CLL/SLL | Non-purified | GSE50006 | GSM1212164 | Affymetrix U133 plus 2.0 | 548 | 312 |
| 335 | CLL/SLL | CLL/SLL | Non-purified | GSE50006 | GSM1212165 | Affymetrix U133 plus 2.0 | 526 | 381 |
| 336 | CLL/SLL | CLL/SLL | Non-purified | GSE50006 | GSM1212166 | Affymetrix U133 plus 2.0 | 559 | 400 |
| 337 | CLL/SLL | CLL/SLL | Non-purified | GSE50006 | GSM1212167 | Affymetrix U133 plus 2.0 | 541 | 329 |
| 338 | CLL/SLL | CLL/SLL | Non-purified | GSE50006 | GSM1212168 | Affymetrix U133 plus 2.0 | 538 | 309 |
| 339 | CLL/SLL | CLL/SLL | Non-purified | GSE50006 | GSM1212169 | Affymetrix U133 plus 2.0 | 596 | 370 |
| 340 | CLL/SLL | CLL/SLL | Non-purified | GSE50006 | GSM1212170 | Affymetrix U133 plus 2.0 | 546 | 414 |
| 341 | CLL/SLL | CLL/SLL | Non-purified | GSE50006 | GSM1212171 | Affymetrix U133 plus 2.0 | 540 | 310 |
| 342 | CLL/SLL | CLL/SLL | Non-purified | GSE50006 | GSM1212172 | Affymetrix U133 plus 2.0 | 545 | 375 |
| 343 | CLL/SLL | CLL/SLL | Non-purified | GSE50006 | GSM1212173 | Affymetrix U133 plus 2.0 | 544 | 412 |
| 344 | FL | FL | Purified | GSE16455 | GSM413651 | Affymetrix U133 plus 2.0 | 253 | 628 |
| 345 | FL | FL | Purified | GSE16455 | GSM413652 | Affymetrix U133 plus 2.0 | 249 | 619 |
| 346 | FL | FL | Purified | GSE16455 | GSM413653 | Affymetrix U133 plus 2.0 | 200 | 861 |
| 347 | FL | FL | Purified | GSE16455 | GSM413654 | Affymetrix U133 plus 2.0 | 244 | 622 |
| 348 | FL | FL | Purified | GSE16455 | GSM413655 | Affymetrix U133 plus 2.0 | 251 | 630 |
| 349 | FL | FL | Purified | GSE16455 | GSM413656 | Affymetrix U133 plus 2.0 | 242 | 626 |
| 350 | FL | FL | Purified | GSE16455 | GSM413657 | Affymetrix U133 plus 2.0 | 240 | 624 |
| 351 | FL | FL | Purified | GSE79196 | GSM2087693 | Affymetrix U133 plus 2.0 | 254 | 629 |
| 352 | FL | FL | Purified | GSE79196 | GSM2087694 | Affymetrix U133 plus 2.0 | 250 | 620 |
| 353 | FL | FL | Purified | GSE79196 | GSM2087695 | Affymetrix U133 plus 2.0 | 241 | 625 |
| 354 | FL | FL | Purified | GSE79196 | GSM2087696 | Affymetrix U133 plus 2.0 | 245 | 623 |
| 355 | FL | FL | Purified | GSE79196 | GSM2087697 | Affymetrix U133 plus 2.0 | 201 | 862 |
| 356 | FL | FL | Purified | GSE79196 | GSM2087698 | Affymetrix U133 plus 2.0 | 243 | 627 |
| 357 | FL | FL | Purified | GSE79196 | GSM2087699 | Affymetrix U133 plus 2.0 | 247 | 632 |
| 358 | FL | FL | Purified | GSE79196 | GSM2087700 | Affymetrix U133 plus 2.0 | 252 | 631 |
| 359 | FL | FL | Purified | GSE79196 | GSM2087701 | Affymetrix U133 plus 2.0 | 248 | 633 |
| 360 | FL | FL | Purified | GSE79196 | GSM2087735 | Affymetrix U133 plus 2.0 | 129 | 550 |
| 361 | FL | FL | Purified | GSE79196 | GSM2087887 | Affymetrix U133 plus 2.0 | 283 | 621 |
| 362 | FL | FL | Purified | GSE79196 | GSM2087888 | Affymetrix U133 plus 2.0 | 246 | 634 |
| 363 | FL | FL | Purified | GSE12453 | GSM312845 | Affymetrix U133 plus 2.0 | 736 | 31 |
| 364 | FL | FL | Purified | GSE12453 | GSM312846 | Affymetrix U133 plus 2.0 | 735 | 33 |
| 365 | FL | FL | Purified | GSE12453 | GSM312847 | Affymetrix U133 plus 2.0 | 734 | 32 |
| 366 | FL | FL | Purified | GSE12453 | GSM312848 | Affymetrix U133 plus 2.0 | 733 | 30 |
| 367 | FL | FL | Purified | GSE12453 | GSM312849 | Affymetrix U133 plus 2.0 | 737 | 34 |
| 368 | FL | FL | Purified | GSE56311 | GSM3164216 | Affymetrix U133 plus 2.0 | 7 | 665 |
| 369 | FL | FL | Purified | GSE56311 | GSM3164217 | Affymetrix U133 plus 2.0 | 10 | 670 |
| 370 | FL | FL | Purified | GSE56311 | GSM3164218 | Affymetrix U133 plus 2.0 | 15 | 663 |
| 371 | FL | FL | Purified | GSE56311 | GSM3164219 | Affymetrix U133 plus 2.0 | 38 | 646 |
| 372 | FL | FL | Purified | GSE56311 | GSM3164220 | Affymetrix U133 plus 2.0 | 40 | 642 |
| 373 | FL | FL | Purified | GSE56311 | GSM3164221 | Affymetrix U133 plus 2.0 | 41 | 643 |
| 374 | FL | FL | Purified | GSE56311 | GSM3164222 | Affymetrix U133 plus 2.0 | 37 | 647 |
| 375 | FL | FL | Purified | GSE56311 | GSM3164223 | Affymetrix U133 plus 2.0 | 6 | 615 |
| 376 | FL | FL | Purified | GSE56311 | GSM3164224 | Affymetrix U133 plus 2.0 | 29 | 651 |
| 377 | FL | FL | Purified | GSE56311 | GSM3164225 | Affymetrix U133 plus 2.0 | 30 | 638 |
| 378 | FL | FL | Purified | GSE56311 | GSM3164226 | Affymetrix U133 plus 2.0 | 39 | 641 |
| 379 | FL | FL | Purified | GSE56311 | GSM3164227 | Affymetrix U133 plus 2.0 | 816 | 909 |
| 380 | FL | FL | Purified | GSE56311 | GSM3164228 | Affymetrix U133 plus 2.0 | 19 | 659 |
| 381 | FL | FL | Purified | GSE56311 | GSM3164229 | Affymetrix U133 plus 2.0 | 20 | 660 |
| 382 | FL | FL | Purified | GSE56311 | GSM3164230 | Affymetrix U133 plus 2.0 | 21 | 658 |
| 383 | FL | FL | Purified | GSE56311 | GSM3164231 | Affymetrix U133 plus 2.0 | 22 | 664 |
| 384 | FL | FL | Purified | GSE56311 | GSM3164232 | Affymetrix U133 plus 2.0 | 26 | 648 |
| 385 | FL | FL | Purified | GSE56311 | GSM3164233 | Affymetrix U133 plus 2.0 | 27 | 649 |
| 386 | FL | FL | Purified | GSE56311 | GSM3164234 | Affymetrix U133 plus 2.0 | 28 | 650 |
| 387 | FL | FL | Purified | GSE56311 | GSM3164235 | Affymetrix U133 plus 2.0 | 35 | 617 |
| 388 | FL | FL | Purified | GSE56311 | GSM3164236 | Affymetrix U133 plus 2.0 | 36 | 618 |
| 389 | FL | FL | Purified | GSE56311 | GSM3164237 | Affymetrix U133 plus 2.0 | 34 | 616 |
| 390 | FL | FL | Purified | GSE56311 | GSM3164238 | Affymetrix U133 plus 2.0 | 24 | 661 |
| 391 | FL | FL | Purified | GSE56311 | GSM3164239 | Affymetrix U133 plus 2.0 | 25 | 662 |
| 392 | FL | FL | Purified | GSE56311 | GSM3164240 | Affymetrix U133 plus 2.0 | 32 | 652 |
| 393 | FL | FL | Purified | GSE56311 | GSM3164241 | Affymetrix U133 plus 2.0 | 33 | 653 |
| 394 | FL | FL | Purified | GSE56311 | GSM3164242 | Affymetrix U133 plus 2.0 | 31 | 657 |
| 395 | FL | FL | Purified | GSE56311 | GSM3164243 | Affymetrix U133 plus 2.0 | 14 | 667 |
| 396 | FL | FL | Purified | GSE56311 | GSM3164244 | Affymetrix U133 plus 2.0 | 8 | 666 |
| 397 | FL | FL | Purified | GSE56311 | GSM3164245 | Affymetrix U133 plus 2.0 | 23 | 654 |
| 398 | FL | FL | Purified | GSE56311 | GSM3164246 | Affymetrix U133 plus 2.0 | 16 | 668 |
| 399 | FL | FL | Purified | GSE56311 | GSM3164247 | Affymetrix U133 plus 2.0 | 17 | 655 |
| 400 | FL | FL | Purified | GSE56311 | GSM3164248 | Affymetrix U133 plus 2.0 | 18 | 656 |
| 401 | FL | FL | Purified | GSE56311 | GSM3164249 | Affymetrix U133 plus 2.0 | 12 | 644 |
| 402 | FL | FL | Purified | GSE56311 | GSM3164250 | Affymetrix U133 plus 2.0 | 13 | 645 |
| 403 | FL | FL | Non-purified | GSE21554 | GSM868103 | Affymetrix U133 plus 2.0 | 741 | 127 |
| 404 | FL | FL | Non-purified | GSE21554 | GSM868104 | Affymetrix U133 plus 2.0 | 946 | 250 |
| 405 | FL | FL | Non-purified | GSE21554 | GSM868105 | Affymetrix U133 plus 2.0 | 746 | 130 |
| 406 | FL | FL | Non-purified | GSE21554 | GSM868106 | Affymetrix U133 plus 2.0 | 743 | 136 |
| 407 | FL | FL | Non-purified | GSE21554 | GSM868107 | Affymetrix U133 plus 2.0 | 740 | 132 |
| 408 | FL | FL | Non-purified | GSE39577 | GSM971906 | Affymetrix U133 plus 2.0 | 992 | 114 |
| 409 | FL | FL | Non-purified | GSE39577 | GSM971907 | Affymetrix U133 plus 2.0 | 1023 | 223 |
| 410 | FL | FL | Non-purified | GSE53820 | GSM1301481 | Affymetrix U133 plus 2.0 | 982 | 195 |
| 411 | FL | FL | Non-purified | GSE53820 | GSM1301482 | Affymetrix U133 plus 2.0 | 922 | 201 |
| 412 | FL | FL | Non-purified | GSE53820 | GSM1301483 | Affymetrix U133 plus 2.0 | 916 | 125 |
| 413 | FL | FL | Non-purified | GSE53820 | GSM1301484 | Affymetrix U133 plus 2.0 | 973 | 189 |
| 414 | FL | FL | Non-purified | GSE53820 | GSM1301485 | Affymetrix U133 plus 2.0 | 928 | 184 |
| 415 | FL | FL | Non-purified | GSE53820 | GSM1301486 | Affymetrix U133 plus 2.0 | 929 | 185 |
| 416 | FL | FL | Non-purified | GSE53820 | GSM1301487 | Affymetrix U133 plus 2.0 | 930 | 197 |
| 417 | FL | FL | Non-purified | GSE53820 | GSM1301488 | Affymetrix U133 plus 2.0 | 917 | 126 |
| 418 | FL | FL | Non-purified | GSE53820 | GSM1301489 | Affymetrix U133 plus 2.0 | 958 | 166 |
| 419 | FL | FL | Non-purified | GSE53820 | GSM1301490 | Affymetrix U133 plus 2.0 | 914 | 160 |
| 420 | FL | FL | Non-purified | GSE53820 | GSM1301491 | Affymetrix U133 plus 2.0 | 951 | 146 |
| 421 | FL | FL | Non-purified | GSE53820 | GSM1301492 | Affymetrix U133 plus 2.0 | 974 | 190 |
| 422 | FL | FL | Non-purified | GSE53820 | GSM1301493 | Affymetrix U133 plus 2.0 | 979 | 199 |
| 423 | FL | FL | Non-purified | GSE53820 | GSM1301494 | Affymetrix U133 plus 2.0 | 985 | 191 |
| 424 | FL | FL | Non-purified | GSE53820 | GSM1301495 | Affymetrix U133 plus 2.0 | 975 | 198 |
| 425 | FL | FL | Non-purified | GSE53820 | GSM1301496 | Affymetrix U133 plus 2.0 | 953 | 174 |
| 426 | FL | FL | Non-purified | GSE53820 | GSM1301498 | Affymetrix U133 plus 2.0 | 955 | 141 |
| 427 | FL | FL | Non-purified | GSE53820 | GSM1301499 | Affymetrix U133 plus 2.0 | 986 | 186 |
| 428 | FL | FL | Non-purified | GSE53820 | GSM1301500 | Affymetrix U133 plus 2.0 | 744 | 133 |
| 429 | FL | FL | Non-purified | GSE53820 | GSM1301502 | Affymetrix U133 plus 2.0 | 980 | 176 |
| 430 | FL | FL | Non-purified | GSE53820 | GSM1301503 | Affymetrix U133 plus 2.0 | 933 | 148 |
| 431 | FL | FL | Non-purified | GSE53820 | GSM1301504 | Affymetrix U133 plus 2.0 | 959 | 173 |
| 432 | FL | FL | Non-purified | GSE53820 | GSM1301505 | Affymetrix U133 plus 2.0 | 984 | 194 |
| 433 | FL | FL | Non-purified | GSE53820 | GSM1301506 | Affymetrix U133 plus 2.0 | 942 | 167 |
| 434 | FL | FL | Non-purified | GSE53820 | GSM1301507 | Affymetrix U133 plus 2.0 | 943 | 158 |
| 435 | FL | FL | Non-purified | GSE53820 | GSM1301509 | Affymetrix U133 plus 2.0 | 918 | 180 |
| 436 | FL | FL | Non-purified | GSE53820 | GSM1301510 | Affymetrix U133 plus 2.0 | 742 | 131 |
| 437 | FL | FL | Non-purified | GSE53820 | GSM1301511 | Affymetrix U133 plus 2.0 | 965 | 152 |
| 438 | FL | FL | Non-purified | GSE53820 | GSM1301512 | Affymetrix U133 plus 2.0 | 949 | 143 |
| 439 | FL | FL | Non-purified | GSE53820 | GSM1301513 | Affymetrix U133 plus 2.0 | 931 | 210 |
| 440 | FL | FL | Non-purified | GSE53820 | GSM1301514 | Affymetrix U133 plus 2.0 | 947 | 139 |
| 441 | FL | FL | Non-purified | GSE53820 | GSM1301515 | Affymetrix U133 plus 2.0 | 960 | 154 |
| 442 | FL | FL | Non-purified | GSE53820 | GSM1301516 | Affymetrix U133 plus 2.0 | 981 | 181 |
| 443 | FL | FL | Non-purified | GSE53820 | GSM1301517 | Affymetrix U133 plus 2.0 | 948 | 140 |
| 444 | FL | FL | Non-purified | GSE53820 | GSM1301518 | Affymetrix U133 plus 2.0 | 919 | 187 |
| 445 | FL | FL | Non-purified | GSE53820 | GSM1301519 | Affymetrix U133 plus 2.0 | 971 | 159 |
| 446 | FL | FL | Non-purified | GSE53820 | GSM1301520 | Affymetrix U133 plus 2.0 | 964 | 179 |
| 447 | FL | FL | Non-purified | GSE53820 | GSM1301522 | Affymetrix U133 plus 2.0 | 915 | 161 |
| 448 | FL | FL | Non-purified | GSE53820 | GSM1301523 | Affymetrix U133 plus 2.0 | 747 | 135 |
| 449 | FL | FL | Non-purified | GSE53820 | GSM1301524 | Affymetrix U133 plus 2.0 | 939 | 164 |
| 450 | FL | FL | Non-purified | GSE53820 | GSM1301525 | Affymetrix U133 plus 2.0 | 968 | 123 |
| 451 | FL | FL | Non-purified | GSE53820 | GSM1301526 | Affymetrix U133 plus 2.0 | 944 | 162 |
| 452 | FL | FL | Non-purified | GSE53820 | GSM1301527 | Affymetrix U133 plus 2.0 | 962 | 156 |
| 453 | FL | FL | Non-purified | GSE53820 | GSM1301528 | Affymetrix U133 plus 2.0 | 926 | 182 |
| 454 | FL | FL | Non-purified | GSE53820 | GSM1301529 | Affymetrix U133 plus 2.0 | 927 | 177 |
| 455 | FL | FL | Non-purified | GSE53820 | GSM1301530 | Affymetrix U133 plus 2.0 | 940 | 165 |
| 456 | FL | FL | Non-purified | GSE53820 | GSM1301531 | Affymetrix U133 plus 2.0 | 983 | 196 |
| 457 | FL | FL | Non-purified | GSE53820 | GSM1301532 | Affymetrix U133 plus 2.0 | 935 | 117 |
| 458 | FL | FL | Non-purified | GSE53820 | GSM1301533 | Affymetrix U133 plus 2.0 | 936 | 163 |
| 459 | FL | FL | Non-purified | GSE53820 | GSM1301534 | Affymetrix U133 plus 2.0 | 961 | 155 |
| 460 | FL | FL | Non-purified | GSE53820 | GSM1301535 | Affymetrix U133 plus 2.0 | 976 | 200 |
| 461 | FL | FL | Non-purified | GSE53820 | GSM1301536 | Affymetrix U133 plus 2.0 | 970 | 188 |
| 462 | FL | FL | Non-purified | GSE53820 | GSM1301538 | Affymetrix U133 plus 2.0 | 923 | 202 |
| 463 | FL | FL | Non-purified | GSE53820 | GSM1301539 | Affymetrix U133 plus 2.0 | 925 | 183 |
| 464 | FL | FL | Non-purified | GSE53820 | GSM1301540 | Affymetrix U133 plus 2.0 | 967 | 138 |
| 465 | FL | FL | Non-purified | GSE53820 | GSM1301541 | Affymetrix U133 plus 2.0 | 934 | 150 |
| 466 | FL | FL | Non-purified | GSE53820 | GSM1301542 | Affymetrix U133 plus 2.0 | 924 | 134 |
| 467 | FL | FL | Non-purified | GSE53820 | GSM1301543 | Affymetrix U133 plus 2.0 | 969 | 145 |
| 468 | FL | FL | Non-purified | GSE53820 | GSM1301544 | Affymetrix U133 plus 2.0 | 966 | 153 |
| 469 | FL | FL | Non-purified | GSE53820 | GSM1301545 | Affymetrix U133 plus 2.0 | 745 | 128 |
| 470 | FL | FL | Non-purified | GSE53820 | GSM1301546 | Affymetrix U133 plus 2.0 | 950 | 144 |
| 471 | FL | FL | Non-purified | GSE53820 | GSM1301547 | Affymetrix U133 plus 2.0 | 956 | 142 |
| 472 | FL | FL | Non-purified | GSE53820 | GSM1301548 | Affymetrix U133 plus 2.0 | 963 | 157 |
| 473 | FL | FL | Non-purified | GSE53820 | GSM1301549 | Affymetrix U133 plus 2.0 | 957 | 149 |
| 474 | FL | FL | Non-purified | GSE53820 | GSM1301550 | Affymetrix U133 plus 2.0 | 938 | 129 |
| 475 | FL | FL | Non-purified | GSE53820 | GSM1301551 | Affymetrix U133 plus 2.0 | 977 | 192 |
| 476 | FL | FL | Non-purified | GSE53820 | GSM1301552 | Affymetrix U133 plus 2.0 | 978 | 193 |
| 477 | FL | FL | Non-purified | GSE53820 | GSM1301553 | Affymetrix U133 plus 2.0 | 748 | 124 |
| 478 | FL | FL | Non-purified | GSE53820 | GSM1301554 | Affymetrix U133 plus 2.0 | 945 | 151 |
| 479 | FL | FL | Non-purified | GSE53820 | GSM1301556 | Affymetrix U133 plus 2.0 | 952 | 147 |
| 480 | FL | FL | Non-purified | GSE53820 | GSM1301557 | Affymetrix U133 plus 2.0 | 1024 | 244 |
| 481 | FL | FL | Non-purified | GSE53820 | GSM1301558 | Affymetrix U133 plus 2.0 | 954 | 175 |
| 482 | FL | FL | Non-purified | GSE53820 | GSM1301559 | Affymetrix U133 plus 2.0 | 972 | 178 |
| 483 | FL | FL | Non-purified | GSE53820 | GSM1301560 | Affymetrix U133 plus 2.0 | 937 | 118 |
| 484 | FL | FL | Non-purified | GSE53820 | GSM1301561 | Affymetrix U133 plus 2.0 | 932 | 211 |
| 485 | FL | FL | Non-purified | GSE55267 | GSM1332984 | Affymetrix U133 plus 2.0 | 1006 | 248 |
| 486 | FL | FL | Non-purified | GSE55267 | GSM1332985 | Affymetrix U133 plus 2.0 | 882 | 215 |
| 487 | FL | FL | Non-purified | GSE55267 | GSM1332986 | Affymetrix U133 plus 2.0 | 871 | 259 |
| 488 | FL | FL | Non-purified | GSE55267 | GSM1332987 | Affymetrix U133 plus 2.0 | 885 | 275 |
| 489 | FL | FL | Non-purified | GSE55267 | GSM1332988 | Affymetrix U133 plus 2.0 | 883 | 281 |
| 490 | FL | FL | Non-purified | GSE55267 | GSM1332989 | Affymetrix U133 plus 2.0 | 886 | 276 |
| 491 | FL | FL | Non-purified | GSE55267 | GSM1332990 | Affymetrix U133 plus 2.0 | 1005 | 232 |
| 492 | FL | FL | Non-purified | GSE55267 | GSM1332991 | Affymetrix U133 plus 2.0 | 878 | 253 |
| 493 | FL | FL | Non-purified | GSE55267 | GSM1332992 | Affymetrix U133 plus 2.0 | 880 | 273 |
| 494 | FL | FL | Non-purified | GSE55267 | GSM1332993 | Affymetrix U133 plus 2.0 | 911 | 261 |
| 495 | FL | FL | Non-purified | GSE55267 | GSM1332994 | Affymetrix U133 plus 2.0 | 870 | 267 |
| 496 | FL | FL | Non-purified | GSE55267 | GSM1332995 | Affymetrix U133 plus 2.0 | 884 | 274 |
| 497 | FL | FL | Non-purified | GSE55267 | GSM1332996 | Affymetrix U133 plus 2.0 | 891 | 251 |
| 498 | FL | FL | Non-purified | GSE55267 | GSM1332997 | Affymetrix U133 plus 2.0 | 873 | 256 |
| 499 | FL | FL | Non-purified | GSE55267 | GSM1332998 | Affymetrix U133 plus 2.0 | 894 | 246 |
| 500 | FL | FL | Non-purified | GSE55267 | GSM1332999 | Affymetrix U133 plus 2.0 | 71 | 221 |
| 501 | FL | FL | Non-purified | GSE55267 | GSM1333000 | Affymetrix U133 plus 2.0 | 1007 | 249 |
| 502 | FL | FL | Non-purified | GSE55267 | GSM1333001 | Affymetrix U133 plus 2.0 | 895 | 247 |
| 503 | FL | FL | Non-purified | GSE55267 | GSM1333002 | Affymetrix U133 plus 2.0 | 902 | 255 |
| 504 | FL | FL | Non-purified | GSE55267 | GSM1333003 | Affymetrix U133 plus 2.0 | 892 | 252 |
| 505 | FL | FL | Non-purified | GSE55267 | GSM1333004 | Affymetrix U133 plus 2.0 | 887 | 279 |
| 506 | FL | FL | Non-purified | GSE55267 | GSM1333005 | Affymetrix U133 plus 2.0 | 1008 | 170 |
| 507 | FL | FL | Non-purified | GSE55267 | GSM1333006 | Affymetrix U133 plus 2.0 | 893 | 277 |
| 508 | FL | FL | Non-purified | GSE55267 | GSM1333007 | Affymetrix U133 plus 2.0 | 898 | 280 |
| 509 | FL | FL | Non-purified | GSE55267 | GSM1333008 | Affymetrix U133 plus 2.0 | 881 | 272 |
| 510 | FL | FL | Non-purified | GSE55267 | GSM1333009 | Affymetrix U133 plus 2.0 | 879 | 254 |
| 511 | FL | FL | Non-purified | GSE55267 | GSM1333010 | Affymetrix U133 plus 2.0 | 877 | 245 |
| 512 | FL | FL | Non-purified | GSE55267 | GSM1333011 | Affymetrix U133 plus 2.0 | 888 | 171 |
| 513 | FL | FL | Non-purified | GSE55267 | GSM1333012 | Affymetrix U133 plus 2.0 | 875 | 258 |
| 514 | FL | FL | Non-purified | GSE55267 | GSM1333013 | Affymetrix U133 plus 2.0 | 889 | 282 |
| 515 | FL | FL | Non-purified | GSE55267 | GSM1333014 | Affymetrix U133 plus 2.0 | 890 | 278 |
| 516 | FL | FL | Non-purified | GSE55267 | GSM1333021 | Affymetrix U133 plus 2.0 | 755 | 36 |
| 517 | FL | FL | Non-purified | GSE55267 | GSM1333022 | Affymetrix U133 plus 2.0 | 749 | 35 |
| 518 | FL | FL | Non-purified | GSE55267 | GSM1333023 | Affymetrix U133 plus 2.0 | 753 | 38 |
| 519 | FL | FL | Non-purified | GSE55267 | GSM1333024 | Affymetrix U133 plus 2.0 | 752 | 39 |
| 520 | FL | FL | Non-purified | GSE55267 | GSM1333025 | Affymetrix U133 plus 2.0 | 756 | 42 |
| 521 | FL | FL | Non-purified | GSE55267 | GSM1333026 | Affymetrix U133 plus 2.0 | 908 | 239 |
| 522 | FL | FL | Non-purified | GSE55267 | GSM1333027 | Affymetrix U133 plus 2.0 | 754 | 37 |
| 523 | FL | FL | Non-purified | GSE55267 | GSM1333028 | Affymetrix U133 plus 2.0 | 912 | 262 |
| 524 | FL | FL | Non-purified | GSE55267 | GSM1333029 | Affymetrix U133 plus 2.0 | 896 | 233 |
| 525 | FL | FL | Non-purified | GSE55267 | GSM1333030 | Affymetrix U133 plus 2.0 | 868 | 264 |
| 526 | FL | FL | Non-purified | GSE55267 | GSM1333031 | Affymetrix U133 plus 2.0 | 913 | 242 |
| 527 | FL | FL | Non-purified | GSE55267 | GSM1333032 | Affymetrix U133 plus 2.0 | 901 | 137 |
| 528 | FL | FL | Non-purified | GSE55267 | GSM1333033 | Affymetrix U133 plus 2.0 | 869 | 265 |
| 529 | FL | FL | Non-purified | GSE55267 | GSM1333034 | Affymetrix U133 plus 2.0 | 904 | 236 |
| 530 | FL | FL | Non-purified | GSE55267 | GSM1333035 | Affymetrix U133 plus 2.0 | 906 | 240 |
| 531 | FL | FL | Non-purified | GSE55267 | GSM1333036 | Affymetrix U133 plus 2.0 | 750 | 40 |
| 532 | FL | FL | Non-purified | GSE55267 | GSM1333037 | Affymetrix U133 plus 2.0 | 905 | 237 |
| 533 | FL | FL | Non-purified | GSE55267 | GSM1333038 | Affymetrix U133 plus 2.0 | 751 | 41 |
| 534 | FL | FL | Non-purified | GSE55267 | GSM1333039 | Affymetrix U133 plus 2.0 | 903 | 43 |
| 535 | FL | FL | Non-purified | GSE55267 | GSM1333040 | Affymetrix U133 plus 2.0 | 910 | 263 |
| 536 | FL | FL | Non-purified | GSE55267 | GSM1333041 | Affymetrix U133 plus 2.0 | 72 | 222 |
| 537 | FL | FL | Non-purified | GSE55267 | GSM1333042 | Affymetrix U133 plus 2.0 | 876 | 257 |
| 538 | FL | FL | Non-purified | GSE55267 | GSM1333043 | Affymetrix U133 plus 2.0 | 1010 | 228 |
| 539 | FL | FL | Non-purified | GSE55267 | GSM1333044 | Affymetrix U133 plus 2.0 | 872 | 268 |
| 540 | FL | FL | Non-purified | GSE55267 | GSM1333045 | Affymetrix U133 plus 2.0 | 899 | 243 |
| 541 | FL | FL | Non-purified | GSE55267 | GSM1333046 | Affymetrix U133 plus 2.0 | 909 | 266 |
| 542 | FL | FL | Non-purified | GSE55267 | GSM1333047 | Affymetrix U133 plus 2.0 | 897 | 234 |
| 543 | FL | FL | Non-purified | GSE55267 | GSM1333048 | Affymetrix U133 plus 2.0 | 1009 | 169 |
| 544 | FL | FL | Non-purified | GSE55267 | GSM1333049 | Affymetrix U133 plus 2.0 | 907 | 238 |
| 545 | FL | FL | Non-purified | GSE55267 | GSM1333050 | Affymetrix U133 plus 2.0 | 900 | 241 |
| 546 | FL | FL | Non-purified | GSE55267 | GSM1333051 | Affymetrix U133 plus 2.0 | 1011 | 229 |
| 547 | FL | FL | Non-purified | GSE55267 | GSM1333052 | Affymetrix U133 plus 2.0 | 874 | 260 |
| 548 | FL | FL | Non-purified | GSE65135 | GSM1587831 | Affymetrix U133 plus 2.0 | 57 | 204 |
| 549 | FL | FL | Non-purified | GSE65135 | GSM1587832 | Affymetrix U133 plus 2.0 | 58 | 208 |
| 550 | FL | FL | Non-purified | GSE65135 | GSM1587833 | Affymetrix U133 plus 2.0 | 62 | 270 |
| 551 | FL | FL | Non-purified | GSE65135 | GSM1587834 | Affymetrix U133 plus 2.0 | 68 | 269 |
| 552 | FL | FL | Non-purified | GSE65135 | GSM1587835 | Affymetrix U133 plus 2.0 | 69 | 212 |
| 553 | FL | FL | Non-purified | GSE65135 | GSM1587836 | Affymetrix U133 plus 2.0 | 59 | 205 |
| 554 | FL | FL | Non-purified | GSE65135 | GSM1587837 | Affymetrix U133 plus 2.0 | 66 | 209 |
| 555 | FL | FL | Non-purified | GSE65135 | GSM1587838 | Affymetrix U133 plus 2.0 | 63 | 203 |
| 556 | FL | FL | Non-purified | GSE65135 | GSM1587839 | Affymetrix U133 plus 2.0 | 60 | 213 |
| 557 | FL | FL | Non-purified | GSE65135 | GSM1587840 | Affymetrix U133 plus 2.0 | 64 | 206 |
| 558 | FL | FL | Non-purified | GSE65135 | GSM1587841 | Affymetrix U133 plus 2.0 | 67 | 271 |
| 559 | FL | FL | Non-purified | GSE65135 | GSM1587842 | Affymetrix U133 plus 2.0 | 65 | 207 |
| 560 | FL | FL | Non-purified | GSE65135 | GSM1587843 | Affymetrix U133 plus 2.0 | 56 | 227 |
| 561 | FL | FL | Non-purified | GSE65135 | GSM1587844 | Affymetrix U133 plus 2.0 | 61 | 214 |
| 562 | FL | FL | Non-purified | GSE86613 | GSM2306870 | Affymetrix U133 plus 2.0 | 76 | 217 |
| 563 | FL | FL | Non-purified | GSE86613 | GSM2306871 | Affymetrix U133 plus 2.0 | 79 | 235 |
| 564 | FL | FL | Non-purified | GSE86613 | GSM2306872 | Affymetrix U133 plus 2.0 | 78 | 219 |
| 565 | FL | FL | Non-purified | GSE86613 | GSM2306873 | Affymetrix U133 plus 2.0 | 73 | 224 |
| 566 | FL | FL | Non-purified | GSE86613 | GSM2306874 | Affymetrix U133 plus 2.0 | 74 | 225 |
| 567 | FL | FL | Non-purified | GSE86613 | GSM2306875 | Affymetrix U133 plus 2.0 | 80 | 226 |
| 568 | FL | FL | Non-purified | GSE86613 | GSM2306876 | Affymetrix U133 plus 2.0 | 77 | 218 |
| 569 | FL | FL | Non-purified | GSE86613 | GSM2306877 | Affymetrix U133 plus 2.0 | 70 | 216 |
| 570 | FL | FL | Non-purified | GSE86613 | GSM2306878 | Affymetrix U133 plus 2.0 | 75 | 220 |
| 571 | cMCL | cMCL | Purified | GSE36000 | GSM879123 | Affymetrix U133 plus 2.0 | 150 | 702 |
| 572 | cMCL | cMCL | Purified | GSE36000 | GSM879124 | Affymetrix U133 plus 2.0 | 217 | 705 |
| 573 | cMCL | cMCL | Purified | GSE36000 | GSM879149 | Affymetrix U133 plus 2.0 | 139 | 726 |
| 574 | cMCL | cMCL | Purified | GSE36000 | GSM879118 | Affymetrix U133 plus 2.0 | 215 | 694 |
| 575 | cMCL | cMCL | Purified | GSE36000 | GSM879119 | Affymetrix U133 plus 2.0 | 169 | 719 |
| 576 | cMCL | cMCL | Purified | GSE36000 | GSM879120 | Affymetrix U133 plus 2.0 | 153 | 722 |
| 577 | cMCL | cMCL | Purified | GSE36000 | GSM879121 | Affymetrix U133 plus 2.0 | 167 | 716 |
| 578 | cMCL | cMCL | Purified | GSE36000 | GSM879122 | Affymetrix U133 plus 2.0 | 221 | 697 |
| 579 | cMCL | cMCL | Purified | GSE36000 | GSM879125 | Affymetrix U133 plus 2.0 | 219 | 683 |
| 580 | cMCL | cMCL | Purified | GSE36000 | GSM879126 | Affymetrix U133 plus 2.0 | 303 | 672 |
| 581 | cMCL | cMCL | Purified | GSE36000 | GSM879127 | Affymetrix U133 plus 2.0 | 164 | 707 |
| 582 | cMCL | cMCL | Purified | GSE36000 | GSM879129 | Affymetrix U133 plus 2.0 | 234 | 689 |
| 583 | cMCL | cMCL | Purified | GSE36000 | GSM879147 | Affymetrix U133 plus 2.0 | 172 | 713 |
| 584 | cMCL | cMCL | Purified | GSE36000 | GSM879148 | Affymetrix U133 plus 2.0 | 174 | 709 |
| 585 | cMCL | cMCL | Purified | GSE36000 | GSM879155 | Affymetrix U133 plus 2.0 | 236 | 680 |
| 586 | cMCL | cMCL | Purified | GSE16455 | GSM413610 | Affymetrix U133 plus 2.0 | 461 | 693 |
| 587 | cMCL | cMCL | Purified | GSE16455 | GSM413611 | Affymetrix U133 plus 2.0 | 155 | 718 |
| 588 | cMCL | cMCL | Purified | GSE16455 | GSM413612 | Affymetrix U133 plus 2.0 | 152 | 721 |
| 589 | cMCL | cMCL | Purified | GSE16455 | GSM413613 | Affymetrix U133 plus 2.0 | 166 | 715 |
| 590 | cMCL | cMCL | Purified | GSE16455 | GSM413614 | Affymetrix U133 plus 2.0 | 459 | 696 |
| 591 | cMCL | cMCL | Purified | GSE16455 | GSM413615 | Affymetrix U133 plus 2.0 | 171 | 712 |
| 592 | cMCL | cMCL | Purified | GSE16455 | GSM413616 | Affymetrix U133 plus 2.0 | 159 | 728 |
| 593 | cMCL | cMCL | Purified | GSE16455 | GSM413618 | Affymetrix U133 plus 2.0 | 460 | 679 |
| 594 | cMCL | cMCL | Purified | GSE16455 | GSM413619 | Affymetrix U133 plus 2.0 | 160 | 729 |
| 595 | cMCL | cMCL | Purified | GSE16455 | GSM413620 | Affymetrix U133 plus 2.0 | 161 | 730 |
| 596 | cMCL | cMCL | Purified | GSE16455 | GSM413622 | Affymetrix U133 plus 2.0 | 462 | 691 |
| 597 | cMCL | cMCL | Purified | GSE16455 | GSM413623 | Affymetrix U133 plus 2.0 | 149 | 701 |
| 598 | cMCL | cMCL | Purified | GSE16455 | GSM413624 | Affymetrix U133 plus 2.0 | 464 | 704 |
| 599 | cMCL | cMCL | Purified | GSE79196 | GSM2087797 | Affymetrix U133 plus 2.0 | 154 | 723 |
| 600 | cMCL | cMCL | Purified | GSE79196 | GSM2087798 | Affymetrix U133 plus 2.0 | 151 | 703 |
| 601 | cMCL | cMCL | Purified | GSE79196 | GSM2087807 | Affymetrix U133 plus 2.0 | 140 | 727 |
| 602 | cMCL | cMCL | Purified | GSE79196 | GSM2087810 | Affymetrix U133 plus 2.0 | 222 | 698 |
| 603 | cMCL | cMCL | Purified | GSE79196 | GSM2087811 | Affymetrix U133 plus 2.0 | 163 | 725 |
| 604 | cMCL | cMCL | Purified | GSE79196 | GSM2087812 | Affymetrix U133 plus 2.0 | 218 | 706 |
| 605 | cMCL | cMCL | Purified | GSE79196 | GSM2087818 | Affymetrix U133 plus 2.0 | 237 | 681 |
| 606 | cMCL | cMCL | Purified | GSE79196 | GSM2087822 | Affymetrix U133 plus 2.0 | 168 | 717 |
| 607 | cMCL | cMCL | Purified | GSE79196 | GSM2087823 | Affymetrix U133 plus 2.0 | 216 | 695 |
| 608 | cMCL | cMCL | Purified | GSE79196 | GSM2087825 | Affymetrix U133 plus 2.0 | 165 | 708 |
| 609 | cMCL | cMCL | Purified | GSE79196 | GSM2087826 | Affymetrix U133 plus 2.0 | 235 | 690 |
| 610 | cMCL | cMCL | Purified | GSE79196 | GSM2087828 | Affymetrix U133 plus 2.0 | 170 | 720 |
| 611 | cMCL | cMCL | Purified | GSE79196 | GSM2087829 | Affymetrix U133 plus 2.0 | 304 | 673 |
| 612 | cMCL | cMCL | Purified | GSE79196 | GSM2087831 | Affymetrix U133 plus 2.0 | 173 | 714 |
| 613 | cMCL | cMCL | Purified | GSE79196 | GSM2087832 | Affymetrix U133 plus 2.0 | 227 | 677 |
| 614 | cMCL | cMCL | Purified | GSE79196 | GSM2087834 | Affymetrix U133 plus 2.0 | 220 | 684 |
| 615 | cMCL | cMCL | Purified | GSE79196 | GSM2087869 | Affymetrix U133 plus 2.0 | 175 | 710 |
| 616 | cMCL | cMCL | Purified | GSE79196 | GSM2087870 | Affymetrix U133 plus 2.0 | 176 | 711 |
| 617 | cMCL | cMCL | Purified | GSE79196 | GSM2087872 | Affymetrix U133 plus 2.0 | 238 | 688 |
| 618 | cMCL | cMCL | Purified | GSE79196 | GSM2087873 | Affymetrix U133 plus 2.0 | 280 | 699 |
| 619 | cMCL | cMCL | Purified | GSE79196 | GSM2087875 | Affymetrix U133 plus 2.0 | 230 | 692 |
| 620 | cMCL | cMCL | Purified | GSE79196 | GSM2087876 | Affymetrix U133 plus 2.0 | 231 | 686 |
| 621 | cMCL | cMCL | Purified | GSE79196 | GSM2087877 | Affymetrix U133 plus 2.0 | 239 | 682 |
| 622 | cMCL | cMCL | Purified | GSE79196 | GSM2087878 | Affymetrix U133 plus 2.0 | 228 | 676 |
| 623 | cMCL | cMCL | Purified | GSE79196 | GSM2087879 | Affymetrix U133 plus 2.0 | 232 | 685 |
| 624 | cMCL | cMCL | Purified | GSE79196 | GSM2087880 | Affymetrix U133 plus 2.0 | 233 | 687 |
| 625 | cMCL | cMCL | Purified | GSE79196 | GSM2087882 | Affymetrix U133 plus 2.0 | 224 | 700 |
| 626 | cMCL | cMCL | Purified | GSE79196 | GSM2087883 | Affymetrix U133 plus 2.0 | 226 | 674 |
| 627 | cMCL | cMCL | Purified | GSE79196 | GSM2087884 | Affymetrix U133 plus 2.0 | 223 | 675 |
| 628 | cMCL | cMCL | Purified | GSE79196 | GSM2087885 | Affymetrix U133 plus 2.0 | 229 | 678 |
| 629 | cMCL | cMCL | Non-purified | GSE21554 | GSM868108 | Affymetrix U133 plus 2.0 | 739 | 64 |
| 630 | cMCL | cMCL | Non-purified | GSE21554 | GSM868111 | Affymetrix U133 plus 2.0 | 1015 | 46 |
| 631 | cMCL | cMCL | Non-purified | GSE21554 | GSM868112 | Affymetrix U133 plus 2.0 | 738 | 74 |
| 632 | nnMCL | nnMCL | Purified | GSE36000 | GSM879131 | Affymetrix U133 plus 2.0 | 297 | 773 |
| 633 | nnMCL | nnMCL | Purified | GSE36000 | GSM879134 | Affymetrix U133 plus 2.0 | 202 | 844 |
| 634 | nnMCL | nnMCL | Purified | GSE36000 | GSM879135 | Affymetrix U133 plus 2.0 | 277 | 785 |
| 635 | nnMCL | nnMCL | Purified | GSE36000 | GSM879136 | Affymetrix U133 plus 2.0 | 211 | 816 |
| 636 | nnMCL | nnMCL | Purified | GSE36000 | GSM879141 | Affymetrix U133 plus 2.0 | 269 | 765 |
| 637 | nnMCL | nnMCL | Purified | GSE36000 | GSM879143 | Affymetrix U133 plus 2.0 | 288 | 811 |
| 638 | nnMCL | nnMCL | Purified | GSE36000 | GSM879144 | Affymetrix U133 plus 2.0 | 213 | 813 |
| 639 | nnMCL | nnMCL | Purified | GSE36000 | GSM879145 | Affymetrix U133 plus 2.0 | 286 | 827 |
| 640 | nnMCL | nnMCL | Purified | GSE36000 | GSM879146 | Affymetrix U133 plus 2.0 | 299 | 776 |
| 641 | nnMCL | nnMCL | Purified | GSE36000 | GSM879150 | Affymetrix U133 plus 2.0 | 147 | 757 |
| 642 | nnMCL | nnMCL | Purified | GSE36000 | GSM879151 | Affymetrix U133 plus 2.0 | 257 | 789 |
| 643 | nnMCL | nnMCL | Purified | GSE36000 | GSM879152 | Affymetrix U133 plus 2.0 | 259 | 792 |
| 644 | nnMCL | nnMCL | Purified | GSE36000 | GSM879153 | Affymetrix U133 plus 2.0 | 255 | 787 |
| 645 | nnMCL | nnMCL | Purified | GSE36000 | GSM879140 | Affymetrix U133 plus 2.0 | 284 | 759 |
| 646 | nnMCL | nnMCL | Purified | GSE36000 | GSM879154 | Affymetrix U133 plus 2.0 | 295 | 770 |
| 647 | nnMCL | nnMCL | Purified | GSE16455 | GSM413603 | Affymetrix U133 plus 2.0 | 469 | 775 |
| 648 | nnMCL | nnMCL | Purified | GSE16455 | GSM413604 | Affymetrix U133 plus 2.0 | 468 | 767 |
| 649 | nnMCL | nnMCL | Purified | GSE16455 | GSM413605 | Affymetrix U133 plus 2.0 | 470 | 772 |
| 650 | nnMCL | nnMCL | Purified | GSE16455 | GSM413606 | Affymetrix U133 plus 2.0 | 467 | 794 |
| 651 | nnMCL | nnMCL | Purified | GSE16455 | GSM413607 | Affymetrix U133 plus 2.0 | 156 | 778 |
| 652 | nnMCL | nnMCL | Purified | GSE16455 | GSM413608 | Affymetrix U133 plus 2.0 | 465 | 826 |
| 653 | nnMCL | nnMCL | Purified | GSE16455 | GSM413609 | Affymetrix U133 plus 2.0 | 203 | 845 |
| 654 | nnMCL | nnMCL | Purified | GSE79196 | GSM2087799 | Affymetrix U133 plus 2.0 | 296 | 771 |
| 655 | nnMCL | nnMCL | Purified | GSE79196 | GSM2087800 | Affymetrix U133 plus 2.0 | 289 | 812 |
| 656 | nnMCL | nnMCL | Purified | GSE79196 | GSM2087801 | Affymetrix U133 plus 2.0 | 214 | 814 |
| 657 | nnMCL | nnMCL | Purified | GSE79196 | GSM2087802 | Affymetrix U133 plus 2.0 | 264 | 840 |
| 658 | nnMCL | nnMCL | Purified | GSE79196 | GSM2087803 | Affymetrix U133 plus 2.0 | 278 | 786 |
| 659 | nnMCL | nnMCL | Purified | GSE79196 | GSM2087804 | Affymetrix U133 plus 2.0 | 258 | 790 |
| 660 | nnMCL | nnMCL | Purified | GSE79196 | GSM2087805 | Affymetrix U133 plus 2.0 | 260 | 793 |
| 661 | nnMCL | nnMCL | Purified | GSE79196 | GSM2087806 | Affymetrix U133 plus 2.0 | 148 | 758 |
| 662 | nnMCL | nnMCL | Purified | GSE79196 | GSM2087808 | Affymetrix U133 plus 2.0 | 256 | 788 |
| 663 | nnMCL | nnMCL | Purified | GSE79196 | GSM2087809 | Affymetrix U133 plus 2.0 | 268 | 769 |
| 664 | nnMCL | nnMCL | Purified | GSE79196 | GSM2087813 | Affymetrix U133 plus 2.0 | 298 | 774 |
| 665 | nnMCL | nnMCL | Purified | GSE79196 | GSM2087814 | Affymetrix U133 plus 2.0 | 212 | 817 |
| 666 | nnMCL | nnMCL | Purified | GSE79196 | GSM2087815 | Affymetrix U133 plus 2.0 | 146 | 762 |
| 667 | nnMCL | nnMCL | Purified | GSE79196 | GSM2087816 | Affymetrix U133 plus 2.0 | 142 | 756 |
| 668 | nnMCL | nnMCL | Purified | GSE79196 | GSM2087817 | Affymetrix U133 plus 2.0 | 144 | 764 |
| 669 | nnMCL | nnMCL | Purified | GSE79196 | GSM2087819 | Affymetrix U133 plus 2.0 | 272 | 796 |
| 670 | nnMCL | nnMCL | Purified | GSE79196 | GSM2087820 | Affymetrix U133 plus 2.0 | 300 | 777 |
| 671 | nnMCL | nnMCL | Purified | GSE79196 | GSM2087821 | Affymetrix U133 plus 2.0 | 270 | 766 |
| 672 | nnMCL | nnMCL | Purified | GSE79196 | GSM2087824 | Affymetrix U133 plus 2.0 | 262 | 780 |
| 673 | nnMCL | nnMCL | Purified | GSE79196 | GSM2087827 | Affymetrix U133 plus 2.0 | 287 | 828 |
| 674 | nnMCL | nnMCL | Purified | GSE79196 | GSM2087833 | Affymetrix U133 plus 2.0 | 285 | 760 |
| 675 | nnMCL | nnMCL | Purified | GSE79196 | GSM2087871 | Affymetrix U133 plus 2.0 | 279 | 798 |
| 676 | nnMCL | nnMCL | Purified | GSE79196 | GSM2087874 | Affymetrix U133 plus 2.0 | 281 | 791 |
| 677 | nnMCL | nnMCL | Purified | GSE79196 | GSM2087881 | Affymetrix U133 plus 2.0 | 225 | 799 |
| 678 | nnMCL | nnMCL | Purified | GSE36000 | GSM879128 | Affymetrix U133 plus 2.0 | 162 | 724 |
| 679 | nnMCL | nnMCL | Purified | GSE36000 | GSM879130 | Affymetrix U133 plus 2.0 | 267 | 768 |
| 680 | nnMCL | nnMCL | Purified | GSE36000 | GSM879132 | Affymetrix U133 plus 2.0 | 271 | 795 |
| 681 | nnMCL | nnMCL | Purified | GSE36000 | GSM879133 | Affymetrix U133 plus 2.0 | 261 | 779 |
| 682 | nnMCL | nnMCL | Purified | GSE36000 | GSM879137 | Affymetrix U133 plus 2.0 | 145 | 761 |
| 683 | nnMCL | nnMCL | Purified | GSE36000 | GSM879138 | Affymetrix U133 plus 2.0 | 141 | 755 |
| 684 | nnMCL | nnMCL | Purified | GSE36000 | GSM879139 | Affymetrix U133 plus 2.0 | 143 | 763 |
| 685 | nnMCL | nnMCL | Purified | GSE36000 | GSM879142 | Affymetrix U133 plus 2.0 | 263 | 839 |
| 686 | nnMCL | nnMCL | Purified | GSE16455 | GSM413617 | Affymetrix U133 plus 2.0 | 276 | 784 |
| 687 | nnMCL | nnMCL | Purified | GSE16455 | GSM413621 | Affymetrix U133 plus 2.0 | 466 | 815 |
| 688 | nnMCL | nnMCL | Non-purified | GSE21554 | GSM868109 | Affymetrix U133 plus 2.0 | 920 | 47 |
| 689 | nnMCL | nnMCL | Non-purified | GSE21554 | GSM868110 | Affymetrix U133 plus 2.0 | 921 | 48 |
| 690 | Other | HCL | Purified | GSE16455 | GSM413626 | Affymetrix U133 plus 2.0 | 180 | 864 |
| 691 | Other | HCL | Purified | GSE16455 | GSM413627 | Affymetrix U133 plus 2.0 | 182 | 866 |
| 692 | Other | HCL | Purified | GSE16455 | GSM413628 | Affymetrix U133 plus 2.0 | 183 | 868 |
| 693 | Other | HCL | Purified | GSE79196 | GSM2087703 | Affymetrix U133 plus 2.0 | 178 | 869 |
| 694 | Other | HCL | Purified | GSE79196 | GSM2087705 | Affymetrix U133 plus 2.0 | 179 | 863 |
| 695 | Other | HCL | Purified | GSE79196 | GSM2087706 | Affymetrix U133 plus 2.0 | 177 | 867 |
| 696 | Other | HCL | Purified | GSE79196 | GSM2087709 | Affymetrix U133 plus 2.0 | 181 | 865 |
| 697 | Other | HCLv | Purified | GSE16455 | GSM413625 | Affymetrix U133 plus 2.0 | 198 | 888 |
| 698 | Other | HCLv | Purified | GSE16455 | GSM413629 | Affymetrix U133 plus 2.0 | 199 | 887 |
| 699 | Other | HCLv | Purified | GSE79196 | GSM2087837 | Affymetrix U133 plus 2.0 | 186 | 882 |
| 700 | Other | HCLv | Purified | GSE79196 | GSM2087845 | Affymetrix U133 plus 2.0 | 184 | 886 |
| 701 | Other | HCLv | Purified | GSE79196 | GSM2087853 | Affymetrix U133 plus 2.0 | 188 | 877 |
| 702 | Other | HCLv | Purified | GSE79196 | GSM2087866 | Affymetrix U133 plus 2.0 | 185 | 880 |
| 703 | Other | SDRPL | Purified | GSE79196 | GSM2087712 | Affymetrix U133 plus 2.0 | 193 | 884 |
| 704 | Other | SDRPL | Purified | GSE79196 | GSM2087789 | Affymetrix U133 plus 2.0 | 209 | 875 |
| 705 | Other | SDRPL | Purified | GSE79196 | GSM2087886 | Affymetrix U133 plus 2.0 | 210 | 879 |
| 706 | Other | SDRPL | Purified | GSE79196 | GSM2087889 | Affymetrix U133 plus 2.0 | 158 | 783 |
| 707 | LPL/WM | LPL/WM | Purified | GSE79196 | GSM2087776 | Affymetrix U133 plus 2.0 | 290 | 810 |
| 708 | LPL/WM | LPL/WM | Purified | GSE79196 | GSM2087777 | Affymetrix U133 plus 2.0 | 265 | 850 |
| 709 | LPL/WM | LPL/WM | Purified | GSE79196 | GSM2087842 | Affymetrix U133 plus 2.0 | 292 | 857 |
| 710 | LPL/WM | LPL/WM | Purified | GSE79196 | GSM2087864 | Affymetrix U133 plus 2.0 | 273 | 855 |
| 711 | LPL/WM | LPL/WM | Purified | GSE9656 | GSM243838 | Affymetrix U133 plus 2.0 | 694 | 989 |
| 712 | LPL/WM | LPL/WM | Purified | GSE9656 | GSM243840 | Affymetrix U133 plus 2.0 | 696 | 984 |
| 713 | LPL/WM | LPL/WM | Purified | GSE9656 | GSM243841 | Affymetrix U133 plus 2.0 | 699 | 985 |
| 714 | LPL/WM | LPL/WM | Purified | GSE9656 | GSM243842 | Affymetrix U133 plus 2.0 | 692 | 995 |
| 715 | LPL/WM | LPL/WM | Purified | GSE9656 | GSM243843 | Affymetrix U133 plus 2.0 | 698 | 993 |
| 716 | LPL/WM | LPL/WM | Purified | GSE9656 | GSM243844 | Affymetrix U133 plus 2.0 | 688 | 991 |
| 717 | LPL/WM | LPL/WM | Purified | GSE9656 | GSM243847 | Affymetrix U133 plus 2.0 | 695 | 990 |
| 718 | LPL/WM | LPL/WM | Purified | GSE9656 | GSM243848 | Affymetrix U133 plus 2.0 | 702 | 982 |
| 719 | LPL/WM | LPL/WM | Purified | GSE9656 | GSM243849 | Affymetrix U133 plus 2.0 | 700 | 994 |
| 720 | LPL/WM | LPL/WM | Purified | GSE9656 | GSM243850 | Affymetrix U133 plus 2.0 | 475 | 1014 |
| 721 | LPL/WM | LPL/WM | Purified | GSE9656 | GSM243851 | Affymetrix U133 plus 2.0 | 701 | 981 |
| 722 | LPL/WM | LPL/WM | Purified | GSE9656 | GSM243852 | Affymetrix U133 plus 2.0 | 689 | 986 |
| 723 | LPL/WM | LPL/WM | Purified | GSE9656 | GSM243853 | Affymetrix U133 plus 2.0 | 728 | 976 |
| 724 | LPL/WM | LPL/WM | Purified | GSE9656 | GSM243857 | Affymetrix U133 plus 2.0 | 724 | 751 |
| 725 | LPL/WM | LPL/WM | Purified | GSE9656 | GSM243858 | Affymetrix U133 plus 2.0 | 731 | 979 |
| 726 | LPL/WM | LPL/WM | Purified | GSE9656 | GSM243859 | Affymetrix U133 plus 2.0 | 732 | 987 |
| 727 | LPL/WM | LPL/WM | Purified | GSE9656 | GSM243860 | Affymetrix U133 plus 2.0 | 730 | 980 |
| 728 | LPL/WM | LPL/WM | Purified | GSE9656 | GSM243861 | Affymetrix U133 plus 2.0 | 725 | 752 |
| 729 | LPL/WM | LPL/WM | Purified | GSE9656 | GSM243862 | Affymetrix U133 plus 2.0 | 729 | 978 |
| 730 | LPL/WM | LPL/WM | Purified | GSE9656 | GSM243863 | Affymetrix U133 plus 2.0 | 723 | 754 |
| 731 | LPL/WM | LPL/WM | Purified | GSE9656 | GSM243864 | Affymetrix U133 plus 2.0 | 727 | 977 |
| 732 | LPL/WM | LPL/WM | Purified | GSE9656 | GSM243866 | Affymetrix U133 plus 2.0 | 690 | 996 |
| 733 | LPL/WM | LPL/WM | Purified | GSE9656 | GSM243867 | Affymetrix U133 plus 2.0 | 691 | 988 |
| 734 | LPL/WM | LPL/WM | Purified | GSE9656 | GSM243868 | Affymetrix U133 plus 2.0 | 703 | 992 |
| 735 | LPL/WM | LPL/WM | Purified | GSE9656 | GSM243872 | Affymetrix U133 plus 2.0 | 726 | 27 |
| 736 | LPL/WM | LPL/WM | Purified | GSE9656 | GSM243873 | Affymetrix U133 plus 2.0 | 697 | 983 |
| 737 | LPL/WM | LPL/WM | Purified | GSE9656 | GSM243874 | Affymetrix U133 plus 2.0 | 693 | 997 |
| 738 | MZL | SMZL | Purified | GSE16455 | GSM413630 | Affymetrix U133 plus 2.0 | 463 | 852 |
| 739 | MZL | SMZL | Purified | GSE16455 | GSM413631 | Affymetrix U133 plus 2.0 | 204 | 847 |
| 740 | MZL | SMZL | Purified | GSE16455 | GSM413632 | Affymetrix U133 plus 2.0 | 208 | 876 |
| 741 | MZL | SMZL | Purified | GSE16455 | GSM413633 | Affymetrix U133 plus 2.0 | 196 | 870 |
| 742 | MZL | SMZL | Purified | GSE79196 | GSM2087702 | Affymetrix U133 plus 2.0 | 194 | 889 |
| 743 | MZL | SMZL | Purified | GSE79196 | GSM2087710 | Affymetrix U133 plus 2.0 | 195 | 885 |
| 744 | MZL | SMZL | Purified | GSE79196 | GSM2087711 | Affymetrix U133 plus 2.0 | 187 | 881 |
| 745 | MZL | SMZL | Purified | GSE79196 | GSM2087778 | Affymetrix U133 plus 2.0 | 324 | 823 |
| 746 | MZL | SMZL | Purified | GSE79196 | GSM2087780 | Affymetrix U133 plus 2.0 | 190 | 873 |
| 747 | MZL | SMZL | Purified | GSE79196 | GSM2087781 | Affymetrix U133 plus 2.0 | 308 | 853 |
| 748 | MZL | SMZL | Purified | GSE79196 | GSM2087782 | Affymetrix U133 plus 2.0 | 205 | 860 |
| 749 | MZL | SMZL | Purified | GSE79196 | GSM2087784 | Affymetrix U133 plus 2.0 | 206 | 848 |
| 750 | MZL | SMZL | Purified | GSE79196 | GSM2087786 | Affymetrix U133 plus 2.0 | 293 | 858 |
| 751 | MZL | SMZL | Purified | GSE79196 | GSM2087790 | Affymetrix U133 plus 2.0 | 274 | 842 |
| 752 | MZL | SMZL | Purified | GSE79196 | GSM2087791 | Affymetrix U133 plus 2.0 | 301 | 841 |
| 753 | MZL | SMZL | Purified | GSE79196 | GSM2087792 | Affymetrix U133 plus 2.0 | 266 | 851 |
| 754 | MZL | SMZL | Purified | GSE79196 | GSM2087793 | Affymetrix U133 plus 2.0 | 302 | 843 |
| 755 | MZL | SMZL | Purified | GSE79196 | GSM2087794 | Affymetrix U133 plus 2.0 | 197 | 871 |
| 756 | MZL | SMZL | Purified | GSE79196 | GSM2087795 | Affymetrix U133 plus 2.0 | 294 | 856 |
| 757 | MZL | SMZL | Purified | GSE79196 | GSM2087840 | Affymetrix U133 plus 2.0 | 309 | 854 |
| 758 | MZL | SMZL | Purified | GSE79196 | GSM2087843 | Affymetrix U133 plus 2.0 | 307 | 883 |
| 759 | MZL | SMZL | Purified | GSE79196 | GSM2087847 | Affymetrix U133 plus 2.0 | 189 | 878 |
| 760 | MZL | SMZL | Purified | GSE79196 | GSM2087848 | Affymetrix U133 plus 2.0 | 207 | 846 |
| 761 | MZL | SMZL | Purified | GSE79196 | GSM2087858 | Affymetrix U133 plus 2.0 | 191 | 874 |
| 762 | MZL | SMZL | Purified | GSE79196 | GSM2087859 | Affymetrix U133 plus 2.0 | 291 | 849 |
| 763 | MZL | SMZL | Purified | GSE79196 | GSM2087861 | Affymetrix U133 plus 2.0 | 192 | 872 |
| 764 | MZL | SMZL | Purified | GSE79196 | GSM2087868 | Affymetrix U133 plus 2.0 | 275 | 859 |
| 765 | MZL | SMZL | Non-purified | GSE21554 | GSM868089 | Affymetrix U133 plus 2.0 | 46 | 62 |
| 766 | MZL | SMZL | Non-purified | GSE21554 | GSM868090 | Affymetrix U133 plus 2.0 | 48 | 56 |
| 767 | MZL | SMZL | Non-purified | GSE21554 | GSM868091 | Affymetrix U133 plus 2.0 | 42 | 65 |
| 768 | MZL | SMZL | Non-purified | GSE21554 | GSM868092 | Affymetrix U133 plus 2.0 | 43 | 66 |
| 769 | MZL | SMZL | Non-purified | GSE21554 | GSM868093 | Affymetrix U133 plus 2.0 | 50 | 52 |
| 770 | MZL | SMZL | Non-purified | GSE21554 | GSM868094 | Affymetrix U133 plus 2.0 | 44 | 59 |
| 771 | MZL | SMZL | Non-purified | GSE21554 | GSM868095 | Affymetrix U133 plus 2.0 | 45 | 60 |
| 772 | MZL | SMZL | Non-purified | GSE21554 | GSM868096 | Affymetrix U133 plus 2.0 | 47 | 63 |
| 773 | MZL | SMZL | Non-purified | GSE21554 | GSM868097 | Affymetrix U133 plus 2.0 | 54 | 53 |
| 774 | MZL | SMZL | Non-purified | GSE21554 | GSM868098 | Affymetrix U133 plus 2.0 | 52 | 58 |
| 775 | MZL | SMZL | Non-purified | GSE21554 | GSM868099 | Affymetrix U133 plus 2.0 | 51 | 57 |
| 776 | MZL | SMZL | Non-purified | GSE21554 | GSM868100 | Affymetrix U133 plus 2.0 | 55 | 54 |
| 777 | MZL | SMZL | Non-purified | GSE21554 | GSM868101 | Affymetrix U133 plus 2.0 | 53 | 61 |
| 778 | MZL | SMZL | Non-purified | GSE21554 | GSM868102 | Affymetrix U133 plus 2.0 | 49 | 55 |
| 779 | MZL | MALTL | Non-purified | GSE39577 | GSM971908 | Affymetrix U133 plus 2.0 | 1020 | 119 |
| 780 | MZL | MALTL | Non-purified | GSE39577 | GSM971909 | Affymetrix U133 plus 2.0 | 1016 | 45 |
| 781 | MZL | MALTL | Non-purified | GSE39577 | GSM971910 | Affymetrix U133 plus 2.0 | 1012 | 49 |
| 782 | MZL | MALTL | Non-purified | GSE39577 | GSM971911 | Affymetrix U133 plus 2.0 | 1021 | 121 |
| 783 | MZL | MALTL | Non-purified | GSE39577 | GSM971912 | Affymetrix U133 plus 2.0 | 1017 | 44 |
| 784 | MZL | MALTL | Non-purified | GSE39577 | GSM971913 | Affymetrix U133 plus 2.0 | 1013 | 50 |
| 785 | MZL | MALTL | Non-purified | GSE39577 | GSM971914 | Affymetrix U133 plus 2.0 | 1014 | 51 |
| 786 | MZL | MALTL | Non-purified | GSE39577 | GSM971915 | Affymetrix U133 plus 2.0 | 1022 | 120 |
| 787 | MZL | MALTL | Non-purified | GSE39577 | GSM971916 | Affymetrix U133 plus 2.0 | 1025 | 107 |
| 788 | MZL | MALTL | Non-purified | GSE39577 | GSM971917 | Affymetrix U133 plus 2.0 | 1026 | 108 |
| 789 | MZL | MALTL | Non-purified | GSE39577 | GSM971918 | Affymetrix U133 plus 2.0 | 997 | 101 |
| 790 | MZL | MALTL | Non-purified | GSE39577 | GSM971919 | Affymetrix U133 plus 2.0 | 990 | 96 |
| 791 | MZL | MALTL | Non-purified | GSE39577 | GSM971920 | Affymetrix U133 plus 2.0 | 1002 | 112 |
| 792 | MZL | MALTL | Non-purified | GSE39577 | GSM971921 | Affymetrix U133 plus 2.0 | 993 | 115 |
| 793 | MZL | MALTL | Non-purified | GSE39577 | GSM971922 | Affymetrix U133 plus 2.0 | 999 | 102 |
| 794 | MZL | MALTL | Non-purified | GSE39577 | GSM971923 | Affymetrix U133 plus 2.0 | 1001 | 105 |
| 795 | MZL | MALTL | Non-purified | GSE39577 | GSM971924 | Affymetrix U133 plus 2.0 | 998 | 106 |
| 796 | MZL | MALTL | Non-purified | GSE39577 | GSM971925 | Affymetrix U133 plus 2.0 | 991 | 97 |
| 797 | MZL | MALTL | Non-purified | GSE39577 | GSM971926 | Affymetrix U133 plus 2.0 | 987 | 109 |
| 798 | MZL | MALTL | Non-purified | GSE39577 | GSM971927 | Affymetrix U133 plus 2.0 | 1028 | 116 |
| 799 | MZL | MALTL | Non-purified | GSE39577 | GSM971928 | Affymetrix U133 plus 2.0 | 996 | 103 |
| 800 | MZL | MALTL | Non-purified | GSE39577 | GSM971929 | Affymetrix U133 plus 2.0 | 994 | 110 |
| 801 | MZL | MALTL | Non-purified | GSE39577 | GSM971930 | Affymetrix U133 plus 2.0 | 1000 | 104 |
| 802 | MZL | MALTL | Non-purified | GSE39577 | GSM971931 | Affymetrix U133 plus 2.0 | 988 | 98 |
| 803 | MZL | MALTL | Non-purified | GSE39577 | GSM971932 | Affymetrix U133 plus 2.0 | 1003 | 111 |
| 804 | MZL | MALTL | Non-purified | GSE39577 | GSM971933 | Affymetrix U133 plus 2.0 | 1004 | 100 |
| 805 | MZL | MALTL | Non-purified | GSE39577 | GSM971934 | Affymetrix U133 plus 2.0 | 1029 | 122 |
| 806 | MZL | MALTL | Non-purified | GSE39577 | GSM971935 | Affymetrix U133 plus 2.0 | 1027 | 168 |
| 807 | MZL | MALTL | Non-purified | GSE39577 | GSM971936 | Affymetrix U133 plus 2.0 | 989 | 99 |
| 808 | MZL | MALTL | Non-purified | GSE39577 | GSM971937 | Affymetrix U133 plus 2.0 | 995 | 113 |
| 809 | Control | FLT | Control | GSE66384 | GSM1621298 | Affymetrix U133 plus 2.0 | 802 | 901 |
| 810 | Control | FLT | Control | GSE66384 | GSM1621299 | Affymetrix U133 plus 2.0 | 794 | 897 |
| 811 | Control | FLT | Control | GSE66384 | GSM1621300 | Affymetrix U133 plus 2.0 | 803 | 896 |
| 812 | Control | FLT | Control | GSE66384 | GSM1621301 | Affymetrix U133 plus 2.0 | 804 | 902 |
| 813 | Control | FLT | Control | GSE66384 | GSM1621302 | Affymetrix U133 plus 2.0 | 805 | 903 |
| 814 | Control | FLT | Control | GSE66384 | GSM1621303 | Affymetrix U133 plus 2.0 | 795 | 898 |
| 815 | Control | FLT | Control | GSE66384 | GSM1621304 | Affymetrix U133 plus 2.0 | 796 | 899 |
| 816 | Control | FLT | Control | GSE66384 | GSM1621305 | Affymetrix U133 plus 2.0 | 799 | 894 |
| 817 | Control | FLT | Control | GSE66384 | GSM1621306 | Affymetrix U133 plus 2.0 | 798 | 893 |
| 818 | Control | FLT | Control | GSE66384 | GSM1621307 | Affymetrix U133 plus 2.0 | 797 | 900 |
| 819 | Control | FLT | Control | GSE66384 | GSM1621308 | Affymetrix U133 plus 2.0 | 800 | 892 |
| 820 | Control | FLT | Control | GSE66384 | GSM1621309 | Affymetrix U133 plus 2.0 | 801 | 895 |
| 821 | Control | FLT | Control | GSE66384 | GSM1621310 | Affymetrix U133 plus 2.0 | 792 | 890 |
| 822 | Control | FLT | Control | GSE66384 | GSM1621311 | Affymetrix U133 plus 2.0 | 793 | 891 |
| 823 | Control | FLT | Control | GSE27928 | GSM690689 | Affymetrix U133 plus 2.0 | 778 | 957 |
| 824 | Control | FLT | Control | GSE27928 | GSM690690 | Affymetrix U133 plus 2.0 | 775 | 950 |
| 825 | Control | FLT | Control | GSE27928 | GSM690691 | Affymetrix U133 plus 2.0 | 780 | 959 |
| 826 | Control | FLT | Control | GSE27928 | GSM690692 | Affymetrix U133 plus 2.0 | 773 | 954 |
| 827 | Control | FLT | Control | GSE27928 | GSM690693 | Affymetrix U133 plus 2.0 | 790 | 948 |
| 828 | Control | FLT | Control | GSE27928 | GSM690694 | Affymetrix U133 plus 2.0 | 776 | 951 |
| 829 | Control | FLT | Control | GSE27928 | GSM690695 | Affymetrix U133 plus 2.0 | 781 | 960 |
| 830 | Control | FLT | Control | GSE27928 | GSM690696 | Affymetrix U133 plus 2.0 | 772 | 958 |
| 831 | Control | FLT | Control | GSE27928 | GSM690697 | Affymetrix U133 plus 2.0 | 779 | 953 |
| 832 | Control | FLT | Control | GSE27928 | GSM690698 | Affymetrix U133 plus 2.0 | 777 | 955 |
| 833 | Control | FLT | Control | GSE27928 | GSM690699 | Affymetrix U133 plus 2.0 | 774 | 952 |
| 834 | Control | FLT | Control | GSE27928 | GSM690700 | Affymetrix U133 plus 2.0 | 757 | 956 |
| 835 | Control | FLT | Control | GSE27928 | GSM690701 | Affymetrix U133 plus 2.0 | 758 | 973 |
| 836 | Control | FLT | Control | GSE27928 | GSM690702 | Affymetrix U133 plus 2.0 | 759 | 971 |
| 837 | Control | FLT | Control | GSE27928 | GSM690703 | Affymetrix U133 plus 2.0 | 760 | 974 |
| 838 | Control | FLT | Control | GSE27928 | GSM690704 | Affymetrix U133 plus 2.0 | 788 | 968 |
| 839 | Control | FLT | Control | GSE27928 | GSM690705 | Affymetrix U133 plus 2.0 | 789 | 972 |
| 840 | Control | FLT | Control | GSE27928 | GSM690706 | Affymetrix U133 plus 2.0 | 761 | 969 |
| 841 | Control | FLT | Control | GSE27928 | GSM690707 | Affymetrix U133 plus 2.0 | 762 | 970 |
| 842 | Control | FLT | Control | GSE27928 | GSM690708 | Affymetrix U133 plus 2.0 | 763 | 975 |
| 843 | Control | FLT | Control | GSE27928 | GSM690709 | Affymetrix U133 plus 2.0 | 791 | 949 |
| 844 | Control | FLT | Control | GSE27928 | GSM690710 | Affymetrix U133 plus 2.0 | 786 | 942 |
| 845 | Control | FLT | Control | GSE27928 | GSM690711 | Affymetrix U133 plus 2.0 | 787 | 943 |
| 846 | Control | FLT | Control | GSE27928 | GSM690712 | Affymetrix U133 plus 2.0 | 764 | 941 |
| 847 | Control | FLT | Control | GSE27928 | GSM690713 | Affymetrix U133 plus 2.0 | 782 | 944 |
| 848 | Control | FLT | Control | GSE27928 | GSM690714 | Affymetrix U133 plus 2.0 | 784 | 946 |
| 849 | Control | FLT | Control | GSE27928 | GSM690715 | Affymetrix U133 plus 2.0 | 785 | 947 |
| 850 | Control | FLT | Control | GSE27928 | GSM690716 | Affymetrix U133 plus 2.0 | 783 | 945 |
| 851 | Control | FLT | Control | GSE27928 | GSM690717 | Affymetrix U133 plus 2.0 | 766 | 962 |
| 852 | Control | FLT | Control | GSE27928 | GSM690718 | Affymetrix U133 plus 2.0 | 769 | 966 |
| 853 | Control | FLT | Control | GSE27928 | GSM690719 | Affymetrix U133 plus 2.0 | 765 | 961 |
| 854 | Control | FLT | Control | GSE27928 | GSM690720 | Affymetrix U133 plus 2.0 | 767 | 963 |
| 855 | Control | FLT | Control | GSE27928 | GSM690721 | Affymetrix U133 plus 2.0 | 768 | 965 |
| 856 | Control | FLT | Control | GSE27928 | GSM690722 | Affymetrix U133 plus 2.0 | 770 | 967 |
| 857 | Control | FLT | Control | GSE27928 | GSM690723 | Affymetrix U133 plus 2.0 | 771 | 964 |
| 858 | Control | FLT | Control | GSE56311 | GSM3164251 | Affymetrix U133 plus 2.0 | 826 | 916 |
| 859 | Control | FLT | Control | GSE56311 | GSM3164252 | Affymetrix U133 plus 2.0 | 11 | 671 |
| 860 | Control | FLT | Control | GSE56311 | GSM3164253 | Affymetrix U133 plus 2.0 | 828 | 917 |
| 861 | Control | FLT | Control | GSE56311 | GSM3164254 | Affymetrix U133 plus 2.0 | 811 | 905 |
| 862 | Control | FLT | Control | GSE56311 | GSM3164255 | Affymetrix U133 plus 2.0 | 812 | 906 |
| 863 | Control | FLT | Control | GSE56311 | GSM3164256 | Affymetrix U133 plus 2.0 | 810 | 907 |
| 864 | Control | FLT | Control | GSE56311 | GSM3164257 | Affymetrix U133 plus 2.0 | 814 | 912 |
| 865 | Control | FLT | Control | GSE56311 | GSM3164258 | Affymetrix U133 plus 2.0 | 815 | 913 |
| 866 | Control | FLT | Control | GSE56311 | GSM3164259 | Affymetrix U133 plus 2.0 | 813 | 904 |
| 867 | Control | FLT | Control | GSE56311 | GSM3164260 | Affymetrix U133 plus 2.0 | 835 | 919 |
| 868 | Control | FLT | Control | GSE56311 | GSM3164261 | Affymetrix U133 plus 2.0 | 818 | 929 |
| 869 | Control | FLT | Control | GSE56311 | GSM3164262 | Affymetrix U133 plus 2.0 | 819 | 930 |
| 870 | Control | FLT | Control | GSE56311 | GSM3164263 | Affymetrix U133 plus 2.0 | 9 | 669 |
| 871 | Control | FLT | Control | GSE56311 | GSM3164264 | Affymetrix U133 plus 2.0 | 820 | 928 |
| 872 | Control | FLT | Control | GSE56311 | GSM3164265 | Affymetrix U133 plus 2.0 | 821 | 939 |
| 873 | Control | FLT | Control | GSE56311 | GSM3164266 | Affymetrix U133 plus 2.0 | 824 | 940 |
| 874 | Control | FLT | Control | GSE56311 | GSM3164267 | Affymetrix U133 plus 2.0 | 825 | 938 |
| 875 | Control | FLT | Control | GSE56311 | GSM3164268 | Affymetrix U133 plus 2.0 | 817 | 911 |
| 876 | Control | FLT | Control | GSE56311 | GSM3164269 | Affymetrix U133 plus 2.0 | 833 | 914 |
| 877 | Control | FLT | Control | GSE56311 | GSM3164270 | Affymetrix U133 plus 2.0 | 834 | 915 |
| 878 | Control | FLT | Control | GSE56311 | GSM3164271 | Affymetrix U133 plus 2.0 | 829 | 927 |
| 879 | Control | FLT | Control | GSE56311 | GSM3164272 | Affymetrix U133 plus 2.0 | 831 | 918 |
| 880 | Control | FLT | Control | GSE56311 | GSM3164273 | Affymetrix U133 plus 2.0 | 830 | 924 |
| 881 | Control | FLT | Control | GSE56311 | GSM3164274 | Affymetrix U133 plus 2.0 | 827 | 925 |
| 882 | Control | FLT | Control | GSE56311 | GSM3164275 | Affymetrix U133 plus 2.0 | 832 | 926 |
| 883 | Control | FLT | Control | GSE56311 | GSM3164276 | Affymetrix U133 plus 2.0 | 823 | 936 |
| 884 | Control | FLT | Control | GSE56311 | GSM3164277 | Affymetrix U133 plus 2.0 | 836 | 920 |
| 885 | Control | FLT | Control | GSE56311 | GSM3164278 | Affymetrix U133 plus 2.0 | 822 | 937 |
| 886 | Control | FLT | Control | GSE56311 | GSM3164279 | Affymetrix U133 plus 2.0 | 837 | 910 |
| 887 | Control | FLT | Control | GSE56311 | GSM3164280 | Affymetrix U133 plus 2.0 | 806 | 932 |
| 888 | Control | FLT | Control | GSE56311 | GSM3164281 | Affymetrix U133 plus 2.0 | 807 | 933 |
| 889 | Control | FLT | Control | GSE56311 | GSM3164282 | Affymetrix U133 plus 2.0 | 808 | 931 |
| 890 | Control | FLT | Control | GSE56311 | GSM3164283 | Affymetrix U133 plus 2.0 | 809 | 908 |
| 891 | Control | B-cell | Control | GSE50006 | GSM1211898 | Affymetrix U133 plus 2.0 | 478 | 731 |
| 892 | Control | B-cell | Control | GSE50006 | GSM1211899 | Affymetrix U133 plus 2.0 | 479 | 732 |
| 893 | Control | B-cell | Control | GSE50006 | GSM1211908 | Affymetrix U133 plus 2.0 | 488 | 736 |
| 894 | Control | B-cell | Control | GSE50006 | GSM1211909 | Affymetrix U133 plus 2.0 | 491 | 744 |
| 895 | Control | B-cell | Control | GSE50006 | GSM1211910 | Affymetrix U133 plus 2.0 | 473 | 1011 |
| 896 | Control | B-cell | Control | GSE50006 | GSM1211911 | Affymetrix U133 plus 2.0 | 492 | 745 |
| 897 | Control | B-cell | Control | GSE50006 | GSM1211912 | Affymetrix U133 plus 2.0 | 480 | 742 |
| 898 | Control | B-cell | Control | GSE50006 | GSM1211913 | Affymetrix U133 plus 2.0 | 481 | 743 |
| 899 | Control | B-cell | Control | GSE50006 | GSM1211914 | Affymetrix U133 plus 2.0 | 489 | 739 |
| 900 | Control | B-cell | Control | GSE50006 | GSM1211915 | Affymetrix U133 plus 2.0 | 483 | 741 |
| 901 | Control | B-cell | Control | GSE50006 | GSM1211916 | Affymetrix U133 plus 2.0 | 487 | 740 |
| 902 | Control | B-cell | Control | GSE50006 | GSM1211917 | Affymetrix U133 plus 2.0 | 484 | 738 |
| 903 | Control | B-cell | Control | GSE50006 | GSM1211918 | Affymetrix U133 plus 2.0 | 490 | 746 |
| 904 | Control | B-cell | Control | GSE50006 | GSM1211919 | Affymetrix U133 plus 2.0 | 482 | 733 |
| 905 | Control | B-cell | Control | GSE50006 | GSM1211920 | Affymetrix U133 plus 2.0 | 485 | 747 |
| 906 | Control | B-cell | Control | GSE50006 | GSM1211921 | Affymetrix U133 plus 2.0 | 486 | 737 |
| 907 | Control | B-cell | Control | GSE50006 | GSM1211928 | Affymetrix U133 plus 2.0 | 305 | 829 |
| 908 | Control | B-cell | Control | GSE50006 | GSM1211929 | Affymetrix U133 plus 2.0 | 311 | 833 |
| 909 | Control | B-cell | Control | GSE50006 | GSM1211930 | Affymetrix U133 plus 2.0 | 310 | 831 |
| 910 | Control | B-cell | Control | GSE50006 | GSM1211931 | Affymetrix U133 plus 2.0 | 306 | 830 |
| 911 | Control | B-cell | Control | GSE50006 | GSM1211961 | Affymetrix U133 plus 2.0 | 312 | 834 |
| 912 | Control | B-cell | Control | GSE50006 | GSM1211962 | Affymetrix U133 plus 2.0 | 327 | 825 |
| 913 | Control | B-cell | Control | GSE50006 | GSM1211963 | Affymetrix U133 plus 2.0 | 316 | 832 |
| 914 | Control | B-cell | Control | GSE50006 | GSM1211964 | Affymetrix U133 plus 2.0 | 313 | 837 |
| 915 | Control | B-cell | Control | GSE50006 | GSM1211965 | Affymetrix U133 plus 2.0 | 314 | 835 |
| 916 | Control | B-cell | Control | GSE50006 | GSM1211966 | Affymetrix U133 plus 2.0 | 315 | 836 |
| 917 | Control | B-cell | Control | GSE50006 | GSM1212006 | Affymetrix U133 plus 2.0 | 317 | 838 |
| 918 | Control | B-cell | Control | GSE50006 | GSM1212007 | Affymetrix U133 plus 2.0 | 379 | 570 |
| 919 | Control | B-cell | Control | GSE50006 | GSM1212142 | Affymetrix U133 plus 2.0 | 476 | 734 |
| 920 | Control | B-cell | Control | GSE50006 | GSM1212143 | Affymetrix U133 plus 2.0 | 477 | 735 |
| 921 | Control | LN | Control | GSE55267 | GSM1333015 | Affymetrix U133 plus 2.0 | 721 | 613 |
| 922 | Control | LN | Control | GSE55267 | GSM1333016 | Affymetrix U133 plus 2.0 | 722 | 614 |
| 923 | Control | LN | Control | GSE55267 | GSM1333017 | Affymetrix U133 plus 2.0 | 719 | 611 |
| 924 | Control | LN | Control | GSE55267 | GSM1333018 | Affymetrix U133 plus 2.0 | 720 | 612 |
| 925 | Control | LN | Control | GSE55267 | GSM1333019 | Affymetrix U133 plus 2.0 | 717 | 609 |
| 926 | Control | LN | Control | GSE55267 | GSM1333020 | Affymetrix U133 plus 2.0 | 718 | 610 |
| 927 | Control | Tonsil | Control | GSE65135 | GSM1587845 | Affymetrix U133 plus 2.0 | 4 | 636 |
| 928 | Control | Tonsil | Control | GSE65135 | GSM1587846 | Affymetrix U133 plus 2.0 | 1 | 639 |
| 929 | Control | Tonsil | Control | GSE65135 | GSM1587847 | Affymetrix U133 plus 2.0 | 5 | 637 |
| 930 | Control | Tonsil | Control | GSE65135 | GSM1587848 | Affymetrix U133 plus 2.0 | 3 | 635 |
| 931 | Control | Tonsil | Control | GSE65135 | GSM1587849 | Affymetrix U133 plus 2.0 | 2 | 640 |
| 932 | Control | LN | Control | GSE3526 | GSM80735 | Affymetrix U133 plus 2.0 | 673 | 83 |
| 933 | Control | LN | Control | GSE3526 | GSM80736 | Affymetrix U133 plus 2.0 | 661 | 28 |
| 934 | Control | LN | Control | GSE3526 | GSM80737 | Affymetrix U133 plus 2.0 | 675 | 85 |
| 935 | Control | LN | Control | GSE3526 | GSM80738 | Affymetrix U133 plus 2.0 | 677 | 81 |
| 936 | Control | LN | Control | GSE7307 | GSM176431 | Affymetrix U133 plus 2.0 | 674 | 84 |
| 937 | Control | LN | Control | GSE7307 | GSM176432 | Affymetrix U133 plus 2.0 | 662 | 29 |
| 938 | Control | LN | Control | GSE7307 | GSM176433 | Affymetrix U133 plus 2.0 | 676 | 86 |
| 939 | Control | LN | Control | GSE7307 | GSM176434 | Affymetrix U133 plus 2.0 | 678 | 82 |
| 940 | Control | LN | Control | GSE99316 | GSM1060755 | Affymetrix U133 plus 2.0 | 1018 | 230 |
| 941 | Control | LN | Control | GSE7788 | GSM188680 | Affymetrix U133 plus 2.0 | 941 | 172 |
| 942 | Control | Tonsil | Control | GSE3526 | GSM80886 | Affymetrix U133 plus 2.0 | 669 | 75 |
| 943 | Control | Tonsil | Control | GSE3526 | GSM80889 | Affymetrix U133 plus 2.0 | 671 | 77 |
| 944 | Control | Tonsil | Control | GSE3526 | GSM80901 | Affymetrix U133 plus 2.0 | 667 | 79 |
| 945 | Control | Tonsil | Control | GSE65135 | GSM1587850 | Affymetrix U133 plus 2.0 | 840 | 922 |
| 946 | Control | Tonsil | Control | GSE65135 | GSM1587851 | Affymetrix U133 plus 2.0 | 838 | 935 |
| 947 | Control | Tonsil | Control | GSE65135 | GSM1587852 | Affymetrix U133 plus 2.0 | 841 | 923 |
| 948 | Control | Tonsil | Control | GSE65135 | GSM1587853 | Affymetrix U133 plus 2.0 | 839 | 934 |
| 949 | Control | Tonsil | Control | GSE65135 | GSM1587854 | Affymetrix U133 plus 2.0 | 842 | 921 |
| 950 | Control | Tonsil | Control | GSE7307 | GSM175976 | Affymetrix U133 plus 2.0 | 670 | 76 |
| 951 | Control | Tonsil | Control | GSE7307 | GSM176015 | Affymetrix U133 plus 2.0 | 672 | 78 |
| 952 | Control | Tonsil | Control | GSE7307 | GSM176114 | Affymetrix U133 plus 2.0 | 668 | 80 |
| 953 | Control | Tonsil | Control | GSE99316 | GSM1060748 | Affymetrix U133 plus 2.0 | 1019 | 231 |
| 954 | Control | Spleen | Control | GSE18490 | GSM463935 | Affymetrix U133 plus 2.0 | 1034 | 72 |
| 955 | Control | Spleen | Control | GSE18674 | GSM463935 | Affymetrix U133 plus 2.0 | 1035 | 73 |
| 956 | Control | Spleen | Control | GSE33846 | GSM837765 | Affymetrix U133 plus 2.0 | 1033 | 71 |
| 957 | Control | Spleen | Control | GSE3526 | GSM80807 | Affymetrix U133 plus 2.0 | 680 | 92 |
| 958 | Control | Spleen | Control | GSE3526 | GSM80808 | Affymetrix U133 plus 2.0 | 684 | 90 |
| 959 | Control | Spleen | Control | GSE3526 | GSM80825 | Affymetrix U133 plus 2.0 | 682 | 88 |
| 960 | Control | Spleen | Control | GSE3526 | GSM80826 | Affymetrix U133 plus 2.0 | 686 | 94 |
| 961 | Control | Spleen | Control | GSE57520 | GSM1384133 | Affymetrix U133 plus 2.0 | 1038 | 68 |
| 962 | Control | Spleen | Control | GSE57520 | GSM1384134 | Affymetrix U133 plus 2.0 | 1037 | 67 |
| 963 | Control | Spleen | Control | GSE57520 | GSM1384135 | Affymetrix U133 plus 2.0 | 1039 | 69 |
| 964 | Control | Spleen | Control | GSE7307 | GSM175941 | Affymetrix U133 plus 2.0 | 679 | 87 |
| 965 | Control | Spleen | Control | GSE7307 | GSM176328 | Affymetrix U133 plus 2.0 | 681 | 93 |
| 966 | Control | Spleen | Control | GSE7307 | GSM176329 | Affymetrix U133 plus 2.0 | 685 | 91 |
| 967 | Control | Spleen | Control | GSE7307 | GSM176330 | Affymetrix U133 plus 2.0 | 683 | 89 |
| 968 | Control | Spleen | Control | GSE7307 | GSM176331 | Affymetrix U133 plus 2.0 | 687 | 95 |
| 969 | Control | Spleen | Control | GSE99316 | GSM1060753 | Affymetrix U133 plus 2.0 | 1036 | 70 |
| 970 | Control | Stamoch | Control | GSE18674 | GSM463936 | Affymetrix U133 plus 2.0 | 1031 | 4 |
| 971 | Control | Stamoch | Control | GSE33846 | GSM837766 | Affymetrix U133 plus 2.0 | 1032 | 5 |
| 972 | Control | Stamoch | Control | GSE3526 | GSM80781 | Affymetrix U133 plus 2.0 | 663 | 8 |
| 973 | Control | Stamoch | Control | GSE3526 | GSM80782 | Affymetrix U133 plus 2.0 | 642 | 6 |
| 974 | Control | Stamoch | Control | GSE3526 | GSM80783 | Affymetrix U133 plus 2.0 | 649 | 14 |
| 975 | Control | Stamoch | Control | GSE3526 | GSM80809 | Affymetrix U133 plus 2.0 | 655 | 10 |
| 976 | Control | Stamoch | Control | GSE3526 | GSM80810 | Affymetrix U133 plus 2.0 | 653 | 18 |
| 977 | Control | Stamoch | Control | GSE3526 | GSM80811 | Affymetrix U133 plus 2.0 | 665 | 1 |
| 978 | Control | Stamoch | Control | GSE3526 | GSM80812 | Affymetrix U133 plus 2.0 | 657 | 20 |
| 979 | Control | Stamoch | Control | GSE3526 | GSM80813 | Affymetrix U133 plus 2.0 | 659 | 12 |
| 980 | Control | Stamoch | Control | GSE3526 | GSM80814 | Affymetrix U133 plus 2.0 | 644 | 22 |
| 981 | Control | Stamoch | Control | GSE3526 | GSM80815 | Affymetrix U133 plus 2.0 | 647 | 25 |
| 982 | Control | Stamoch | Control | GSE3526 | GSM80816 | Affymetrix U133 plus 2.0 | 651 | 16 |
| 983 | Control | Stamoch | Control | GSE7307 | GSM175822 | Affymetrix U133 plus 2.0 | 664 | 9 |
| 984 | Control | Stamoch | Control | GSE7307 | GSM175823 | Affymetrix U133 plus 2.0 | 643 | 7 |
| 985 | Control | Stamoch | Control | GSE7307 | GSM175824 | Affymetrix U133 plus 2.0 | 650 | 15 |
| 986 | Control | Stamoch | Control | GSE7307 | GSM175943 | Affymetrix U133 plus 2.0 | 646 | 24 |
| 987 | Control | Stamoch | Control | GSE7307 | GSM176336 | Affymetrix U133 plus 2.0 | 656 | 11 |
| 988 | Control | Stamoch | Control | GSE7307 | GSM176337 | Affymetrix U133 plus 2.0 | 654 | 19 |
| 989 | Control | Stamoch | Control | GSE7307 | GSM176338 | Affymetrix U133 plus 2.0 | 666 | 2 |
| 990 | Control | Stamoch | Control | GSE7307 | GSM176339 | Affymetrix U133 plus 2.0 | 658 | 21 |
| 991 | Control | Stamoch | Control | GSE7307 | GSM176340 | Affymetrix U133 plus 2.0 | 660 | 13 |
| 992 | Control | Stamoch | Control | GSE7307 | GSM176341 | Affymetrix U133 plus 2.0 | 645 | 23 |
| 993 | Control | Stamoch | Control | GSE7307 | GSM176342 | Affymetrix U133 plus 2.0 | 648 | 26 |
| 994 | Control | Stamoch | Control | GSE7307 | GSM176343 | Affymetrix U133 plus 2.0 | 652 | 17 |
| 995 | Control | Stamoch | Control | GSE99316 | GSM1060738 | Affymetrix U133 plus 2.0 | 1030 | 3 |
| 996 | Control | PB | Control | GSE26725 | GSM658010 | Affymetrix U133 plus 2.0 | 321 | 819 |
| 997 | Control | PB | Control | GSE26725 | GSM658011 | Affymetrix U133 plus 2.0 | 319 | 821 |
| 998 | Control | PB | Control | GSE26725 | GSM658012 | Affymetrix U133 plus 2.0 | 320 | 822 |
| 999 | Control | PB | Control | GSE26725 | GSM658013 | Affymetrix U133 plus 2.0 | 322 | 820 |
| 1000 | Control | PB | Control | GSE26725 | GSM658014 | Affymetrix U133 plus 2.0 | 318 | 818 |
| 1001 | Control | PB | Control | GSE99316 | GSM1060759 | Affymetrix U133 plus 2.0 | 474 | 1012 |
| 1002 | Control | BM | Control | GSE11504 | GSM289612 | Affymetrix U133 plus 2.0 | 843 | 1015 |
| 1003 | Control | BM | Control | GSE11504 | GSM289613 | Affymetrix U133 plus 2.0 | 858 | 1018 |
| 1004 | Control | BM | Control | GSE11504 | GSM289614 | Affymetrix U133 plus 2.0 | 860 | 1016 |
| 1005 | Control | BM | Control | GSE11504 | GSM289615 | Affymetrix U133 plus 2.0 | 861 | 1017 |
| 1006 | Control | BM | Control | GSE11504 | GSM289616 | Affymetrix U133 plus 2.0 | 859 | 1019 |
| 1007 | Control | BM | Control | GSE11504 | GSM289617 | Affymetrix U133 plus 2.0 | 844 | 1020 |
| 1008 | Control | BM | Control | GSE11504 | GSM289618 | Affymetrix U133 plus 2.0 | 850 | 1030 |
| 1009 | Control | BM | Control | GSE11504 | GSM289619 | Affymetrix U133 plus 2.0 | 848 | 1021 |
| 1010 | Control | BM | Control | GSE11504 | GSM289620 | Affymetrix U133 plus 2.0 | 851 | 1033 |
| 1011 | Control | BM | Control | GSE11504 | GSM289621 | Affymetrix U133 plus 2.0 | 866 | 1031 |
| 1012 | Control | BM | Control | GSE11504 | GSM289622 | Affymetrix U133 plus 2.0 | 853 | 1023 |
| 1013 | Control | BM | Control | GSE11504 | GSM289623 | Affymetrix U133 plus 2.0 | 846 | 1025 |
| 1014 | Control | BM | Control | GSE11504 | GSM289624 | Affymetrix U133 plus 2.0 | 847 | 1026 |
| 1015 | Control | BM | Control | GSE11504 | GSM289625 | Affymetrix U133 plus 2.0 | 849 | 1022 |
| 1016 | Control | BM | Control | GSE11504 | GSM289626 | Affymetrix U133 plus 2.0 | 845 | 1024 |
| 1017 | Control | BM | Control | GSE11504 | GSM289627 | Affymetrix U133 plus 2.0 | 852 | 1034 |
| 1018 | Control | BM | Control | GSE11504 | GSM289628 | Affymetrix U133 plus 2.0 | 854 | 1032 |
| 1019 | Control | BM | Control | GSE11504 | GSM289629 | Affymetrix U133 plus 2.0 | 856 | 1028 |
| 1020 | Control | BM | Control | GSE11504 | GSM289630 | Affymetrix U133 plus 2.0 | 857 | 1029 |
| 1021 | Control | BM | Control | GSE11504 | GSM289631 | Affymetrix U133 plus 2.0 | 855 | 1027 |
| 1022 | Control | BM | Control | GSE11504 | GSM289632 | Affymetrix U133 plus 2.0 | 867 | 1035 |
| 1023 | Control | BM | Control | GSE11504 | GSM289633 | Affymetrix U133 plus 2.0 | 862 | 1038 |
| 1024 | Control | BM | Control | GSE11504 | GSM289634 | Affymetrix U133 plus 2.0 | 864 | 1036 |
| 1025 | Control | BM | Control | GSE11504 | GSM289635 | Affymetrix U133 plus 2.0 | 865 | 1037 |
| 1026 | Control | BM | Control | GSE11504 | GSM289636 | Affymetrix U133 plus 2.0 | 863 | 1039 |
| 1027 | Control | BM | Control | GSE18674 | GSM463920 | Affymetrix U133 plus 2.0 | 705 | 999 |
| 1028 | Control | BM | Control | GSE33846 | GSM837750 | Affymetrix U133 plus 2.0 | 706 | 1000 |
| 1029 | Control | BM | Control | GSE3526 | GSM80576 | Affymetrix U133 plus 2.0 | 711 | 1007 |
| 1030 | Control | BM | Control | GSE3526 | GSM80577 | Affymetrix U133 plus 2.0 | 713 | 1009 |
| 1031 | Control | BM | Control | GSE3526 | GSM80602 | Affymetrix U133 plus 2.0 | 709 | 1005 |
| 1032 | Control | BM | Control | GSE3526 | GSM80603 | Affymetrix U133 plus 2.0 | 710 | 1006 |
| 1033 | Control | BM | Control | GSE3526 | GSM80604 | Affymetrix U133 plus 2.0 | 707 | 1003 |
| 1034 | Control | BM | Control | GSE7307 | GSM175906 | Affymetrix U133 plus 2.0 | 715 | 1001 |
| 1035 | Control | BM | Control | GSE7307 | GSM175951 | Affymetrix U133 plus 2.0 | 716 | 1002 |
| 1036 | Control | BM | Control | GSE7307 | GSM175974 | Affymetrix U133 plus 2.0 | 712 | 1008 |
| 1037 | Control | BM | Control | GSE7307 | GSM175975 | Affymetrix U133 plus 2.0 | 714 | 1010 |
| 1038 | Control | BM | Control | GSE7307 | GSM176300 | Affymetrix U133 plus 2.0 | 708 | 1004 |
| 1039 | Control | BM | Control | GSE99316 | GSM1060758 | Affymetrix U133 plus 2.0 | 704 | 998 |

Abbreviations: CLL/SLL, chronic lymphocytic leukemia/small lymphocytic lymphoma; cMCL, conventional mantle cell lymphoma; MCL, mantle cell lymphoma; nnMCL, leukemic non-nodal mantle cell lymphoma; FL, follicular lymphoma; MZL, marginal zone lymphoma; SMZL, splenic marginal zone lymphoma; MALTL, extranodal marginal zone lymphoma of mucosa-associated lymphoid tissue; NMZL, nodal marginal zone lymphoma; LPL/WM, lymphoplasmacytic lymphoma/Waldenström’s macroglobulinemia; HCL, hairy cell leukemia; HCLv, hairy cell leukemia-variant; SDRPL, splenic diffuse red pulp small B-cell lymphoma; FLT, T-cells of follicular lymphoma; LN, lymph node; BM, bone marrow.

**Supplementary Table S9. Genes that selected to include in a pilot NanoString codeset**

| **Subgroups** | **Strategy of gene selection** | **No of genes selected** | **Differentially expressed genes (upregulated)** |
| --- | --- | --- | --- |
| Upregulated in CLL/SLL compared with other SBCLNs | One-vs-rest | 16 | *ARHGAP44, CLNK, CCDC88A, ABCA6, ADTRP, ZBTB32, ZBTB24, LEF1, FMOD, ROR1, FCER2, GPM6A, TCF4, TGFBR3, CTLA4, EBF1 (Downregulated)* |
| Upregulated in FL compared with other SBCLNs | One-vs-rest | 15 | *EML6, MME, RGS13, MYBL1, SYBU, IGF2BP3, GPR82, BCL6, SORL1, FAM169A, PAG1, ELL3, CDCA7, RASSF6, CD24* |
| Upregulated in cMCL/nnMCL compared with other SBCLNs | One-vs-rest | 2 | *CCND1, FAM129C* |
| Upregulated in cMCL compared with nnMCL/nnMCL | One-vs-one | 14 | *SOX11, PLEKHG4B, HDGFRP3, CNR1, ZNF711, DCHS1, FNBP1L, PON2, NREP, FARP1, PLXNB1, PAWR, CRIM1, DBN1* |
| Upregulated in nnMCL/nnMCL compared with cMCL | One-vs-one | 7 | *PELI1, COBLL1, NR4A2, CCSER1, SLAMF1, BTLA, CD200* |
| Upregulated in HCL compared with other SBCLNs | One-vs-rest | 20 | *MYF6, SYT1, SUSD5, IL1R2, MFAP5, SOX5, MYOF, PSD3, PTPRM, EMP1, PLOD2, HOPX, TFEC, SLAMF7, TJP1, CSGALNACT1, PCDH9, ZNF226, FCRLA, GLTSCR2* |
| Upregulated in HCLv compared with other SBCLNs | One-vs-rest | 19 | *LRP1B, NETO1, RBPMS2, MPP6, WASF3, MS4A14, NRCAM, NEB, SLC11A1, MID1, THEMIS2, FGD4, ANK3, ITGB2-AS1, ZNF331, HCK, SIGLEC6, RGS2, USP9Y* |
| Upregulated in HCLv/SDRPL compared with other SBCLNs | One-vs-rest | 3 | *MACROD2, RRAGD, MS4A1* |
| Upregulated in SDRPL compared with other SBCLNs | One-vs-rest | 6 | *MEF2A, GEN1, RHOBTB3, ANK2, KRAS, CCDC85A* |
| Upregulated in LPL/WM compared with other SBCLNs | One-vs-rest | 13 | *SDC1, EGFR, FKBP11, KDELR3, PRDM1, PSAT1, CCR2, FNDC3B, ELL2, IGF1, TNFRSF17, LHCGR, IGHM* |
| Upregulated in MALTL/SMZL compared with other SBCLNs | One-vs-rest | 1 | *ANXA4* |
| Upregulated in SMZL compared with other SBCLNs | One-vs-rest | 1 | *FGFBP2* |
| Upregulated in MALTL compared with other SBCLNs | One-vs-rest | 19 | *MMP1, SULF1, STEAP1, POSTN, GREM1, GPNMB, CTGF, DCN, BHLHE41, MYLK, ACTA2, CXCL13, MGP, BASP1, VCAN, CHIT1, BCL10, FAM129A, FYN* |
| Background and other interested genes | Previous studies | 18 | *BCL2, BTG1, CD79B, CLN8, COL1A1, COL3A1, CXCR4, CYR61, DUSP4, HDAC9, IGFBP7, IGHD, KLHL14, MAF, MYC, PAX5, RORA, SEPP1* |
| Housekeeping genes | geNorm algorithm | 11 | *ACTB, GAPDH，CADM2, CFTR, DNAH7, GATM, GRIK2, GUK1, GUSB, PDZD2, RIMS1, SUN5, TMEM239* |

Abbreviations: CLL/SLL, chronic lymphocytic leukemia/small lymphocytic lymphoma; cMCL, conventional mantle cell lymphoma; MCL, mantle cell lymphoma; nnMCL, leukemic non-nodal mantle cell lymphoma; FL, follicular lymphoma; MZL, marginal zone lymphoma; SMZL, splenic marginal zone lymphoma; MALTL, extranodal marginal zone lymphoma of mucosa-associated lymphoid tissue; NMZL, nodal marginal zone lymphoma; LPL/WM, lymphoplasmacytic lymphoma/Waldenström’s macroglobulinemia; HCL, hairy cell leukemia; HCLv, hairy cell leukemia-variant; SDRPL, splenic diffuse red pulp small B-cell lymphoma.

**Supplementary Table S10. DBI of each entity**

| **Entity** | **DBI** |
| --- | --- |
| MZL | 4.11544087 |
| nnMCL | 3.185795719 |
| FL | 3.146564578 |
| cMCL | 2.101356083 |
| CLL/SLL | 1.754652463 |
| LPL/WM | 1.000558708 |
| Lymph node/Waldeyer's ring | 3.942637358 |
| Extranodal tissues | 2.405167702 |
| Peripheral blood/Bone marrow | 1.193768331 |

Abbreviations: CLL/SLL, chronic lymphocytic leukemia/small lymphocytic lymphoma; cMCL, conventional mantle cell lymphoma; MCL, mantle cell lymphoma; nnMCL, leukemic non-nodal mantle cell lymphoma; FL, follicular lymphoma; MZL, marginal zone lymphoma; LPL/WM, lymphoplasmacytic lymphoma/Waldenström’s macroglobulinemia; DBI, Davies-Bouldin Index.

**Supplementary Table S11. Genes that finally selected in building the molecular classifier.**

| **SBCLN entity** | **Gene** | **Expression** | **Gini index** | **Classifier generated from** |
| --- | --- | --- | --- | --- |
| CLL/SLL | *BTLA* | Upregulated | 0.1444 | CLL-vs-remaining |
| CLL/SLL | *ARHGAP44* | Upregulated | 0.1426 | CLL-vs-remaining |
| CLL/SLL | *ZBTB24* | Upregulated | 0.1211 | CLL-vs-remaining |
| CLL/SLL | *CLNK* | Upregulated | 0.0969 | CLL-vs-remaining |
| CLL/SLL | *CD200* | Upregulated | 0.0815 | CLL-vs-remaining |
| cMCL | *SOX11* | Upregulated | 0.2321 | cMCL-vs-remaining |
| cMCL | *PLEKHG4B* | Upregulated | 0.1681 | cMCL-vs-remaining |
| cMCL | *ZNF711* | Upregulated | 0.0892 | cMCL-vs-remaining |
| cMCL | *CCND1* | Upregulated | 0.0881 | cMCL-vs-remaining |
| cMCL | *FAM129C* | Upregulated | 0.0629 | cMCL-vs-remaining |
| cMCL | *ABCA6* | Upregulated | 0.055 | cMCL-vs-remaining |
| FL | *BCL2* | Upregulated | 0.2473 | FL-vs-remaining |
| FL | *EML6* | Upregulated | 0.1262 | FL-vs-remaining |
| FL | *ELL3* | Upregulated | 0.094 | FL-vs-remaining |
| FL | *CTLA4* | Upregulated | 0.0866 | FL-vs-remaining |
| FL | *FCER2* | Upregulated | 0.0596 | FL-vs-remaining |
| FL | *IGF2BP3* | Upregulated | 0.0566 | FL-vs-remaining |
| FL | *RGS13* | Upregulated | 0.0556 | FL-vs-remaining |
| FL | *EBF1* | Upregulated | 0.0387 | FL-vs-remaining |
| nnMCL | *CCND1* | Upregulated | 0.1093 | nnMCL-vs-remaining |
| nnMCL | *ZNF331* | Upregulated | 0.1075 | nnMCL-vs-remaining |
| nnMCL | *PAX5* | Upregulated | 0.0682 | nnMCL-vs-remaining |
| nnMCL | *CNR1* | Upregulated | 0.0622 | nnMCL-vs-remaining |
| MZL | *FCRLA* | Upregulated | 0.1427 | MZL-vs-remaining |
| MZL | *HDAC9* | Upregulated | 0.1282 | MZL-vs-remaining |
| MZL | *MS4A1* | Upregulated | 0.1118 | MZL-vs-remaining |
| MZL | *SIGLEC6* | Upregulated | 0.1047 | MZL-vs-remaining |
| MZL | *ZBTB32* | Upregulated | 0.0847 | MZL-vs-remaining |
| MZL | *BHLHE41* | Upregulated | 0.0709 | MZL-vs-remaining |
| LPL/WM | *CCR2* | - | 0.1 | LPL-vs-remaining |
| LPL/WM | *HOPX* | - | 0.0556 | LPL-vs-remaining |
| LPL/WM | *FKBP11* | - | 0.0556 | LPL-vs-remaining |
| LPL/WM | *ANK3* | - | 0.0556 | LPL-vs-remaining |
| LPL/WM | *ZNF226* | - | 0.0444 | LPL-vs-remaining |
| LPL/WM | *MFAP5* | - | 0.0444 | LPL-vs-remaining |
| LPL/WM | *MEF2A* | - | 0.0444 | LPL-vs-remaining |

Abbreviations: CLL/SLL, chronic lymphocytic leukemia/small lymphocytic lymphoma; cMCL, conventional mantle cell lymphoma; MCL, mantle cell lymphoma; nnMCL, leukemic non-nodal mantle cell lymphoma; FL, follicular lymphoma; MZL, marginal zone lymphoma; LPL/WM, lymphoplasmacytic lymphoma/Waldenström’s macroglobulinemia.

**Supplementary Table S12. The prediction power of the trained model evaluated by leave-one-out cross-validation strategy.**

| **SBCLN entity** | **CLL/SLL** | **cMCL** | **FL** | **nnMCL** | **MZL** | **LPL/WM** | **Control cases** |
| --- | --- | --- | --- | --- | --- | --- | --- |
| CLL/SLL | 7 | 0 | 0 | 0 | 0 | 0 | 0 |
| cMCL | 0 | 9 | 0 | 0 | 0 | 0 | 0 |
| FL | 0 | 0 | 12 | 0 | 0 | 0 | 1 |
| nnMCL | 0 | 0 | 0 | 3 | 0 | 0 | 1 |
| MZL | 0 | 0 | 0 | 0 | 16 | 0 | 3 |
| LPL/WM | 0 | 0 | 0 | 0 | 0 | 5 | 0 |
| Control cases | 0 | 0 | 0 | 1 | 1 | 0 | 100 |

Abbreviations: CLL/SLL, chronic lymphocytic leukemia/small lymphocytic lymphoma; cMCL, conventional mantle cell lymphoma; MCL, mantle cell lymphoma; nnMCL, leukemic non-nodal mantle cell lymphoma; FL, follicular lymphoma; MZL, marginal zone lymphoma; LPL/WM, lymphoplasmacytic lymphoma/Waldenström’s macroglobulinemia.

**Supplementary Table S13. Sensitivity and specificity of our model indicated by validation cohort.**

| **Tumor cell content no less than** | **Sensitivity** | **Specificity** | **Sensitivity (Fresh/Frozen)** | **Specificity (Fresh/Frozen)** | **Sensitivity (FFPE)** | **Specificity (FFPE)** | **Sensitivity (modified model)** | **Specificity (modified model)** |
| --- | --- | --- | --- | --- | --- | --- | --- | --- |
| 0.94 | 1.000 | 1.000 | 1.000 | 1.000 | 1.000 | 1.000 | 1.000 | 1.000 |
| 0.93 | 1.000 | 1.000 | 1.000 | 1.000 | 1.000 | 1.000 | 1.000 | 1.000 |
| 0.92 | 1.000 | 1.000 | 1.000 | 1.000 | 1.000 | 1.000 | 1.000 | 1.000 |
| 0.91 | 1.000 | 1.000 | 1.000 | 1.000 | 1.000 | 1.000 | 1.000 | 1.000 |
| 0.9 | 1.000 | 1.000 | 1.000 | 1.000 | 1.000 | 1.000 | 1.000 | 1.000 |
| 0.89 | 1.000 | 1.000 | 1.000 | 1.000 | 1.000 | 1.000 | 1.000 | 1.000 |
| 0.88 | 1.000 | 1.000 | 1.000 | 1.000 | 1.000 | 1.000 | 1.000 | 1.000 |
| 0.87 | 1.000 | 1.000 | 1.000 | 1.000 | 1.000 | 1.000 | 1.000 | 1.000 |
| 0.86 | 1.000 | 1.000 | 1.000 | 1.000 | 1.000 | 1.000 | 1.000 | 1.000 |
| 0.85 | 1.000 | 1.000 | 1.000 | 1.000 | 1.000 | 1.000 | 1.000 | 1.000 |
| 0.84 | 1.000 | 1.000 | 1.000 | 1.000 | 1.000 | 1.000 | 1.000 | 1.000 |
| 0.83 | 1.000 | 1.000 | 1.000 | 1.000 | 1.000 | 1.000 | 1.000 | 1.000 |
| 0.82 | 1.000 | 0.974 | 1.000 | 1.000 | 0.971 | 0.971 | 1.000 | 1.000 |
| 0.81 | 1.000 | 0.979 | 1.000 | 1.000 | 0.975 | 0.975 | 1.000 | 1.000 |
| 0.8 | 1.000 | 0.979 | 1.000 | 1.000 | 0.976 | 0.976 | 1.000 | 1.000 |
| 0.79 | 1.000 | 0.979 | 1.000 | 1.000 | 0.976 | 0.976 | 1.000 | 1.000 |
| 0.78 | 0.964 | 0.981 | 1.000 | 1.000 | 0.938 | 0.978 | 0.964 | 1.000 |
| 0.77 | 0.964 | 0.981 | 1.000 | 1.000 | 0.939 | 0.979 | 0.964 | 1.000 |
| 0.76 | 0.968 | 0.983 | 1.000 | 1.000 | 0.944 | 0.981 | 0.968 | 1.000 |
| 0.75 | 0.969 | 0.984 | 1.000 | 1.000 | 0.946 | 0.981 | 0.969 | 1.000 |
| 0.74 | 0.956 | 0.985 | 1.000 | 1.000 | 0.933 | 0.982 | 0.956 | 1.000 |
| 0.73 | 0.948 | 0.986 | 1.000 | 1.000 | 0.928 | 0.985 | 0.961 | 1.000 |
| 0.72 | 0.952 | 0.987 | 1.000 | 1.000 | 0.932 | 0.986 | 0.964 | 1.000 |
| 0.71 | 0.924 | 0.988 | 1.000 | 1.000 | 0.900 | 0.986 | 0.957 | 1.000 |
| 0.7 | 0.916 | 0.989 | 1.000 | 1.000 | 0.892 | 0.987 | 0.958 | 1.000 |
| 0.69 | 0.917 | 0.989 | 1.000 | 1.000 | 0.893 | 0.987 | 0.958 | 1.000 |
| 0.68 | 0.900 | 0.989 | 1.000 | 1.000 | 0.874 | 0.987 | 0.960 | 1.000 |
| 0.67 | 0.877 | 0.989 | 1.000 | 1.000 | 0.849 | 0.988 | 0.953 | 1.000 |
| 0.66 | 0.865 | 0.979 | 0.929 | 0.929 | 0.835 | 0.988 | 0.928 | 0.990 |
| 0.65 | 0.868 | 0.980 | 0.929 | 0.929 | 0.840 | 0.988 | 0.930 | 0.991 |
| 0.64 | 0.853 | 0.980 | 0.867 | 0.929 | 0.832 | 0.988 | 0.922 | 0.991 |
| 0.63 | 0.847 | 0.981 | 0.875 | 0.933 | 0.824 | 0.989 | 0.911 | 0.991 |
| 0.62 | 0.836 | 0.981 | 0.875 | 0.933 | 0.813 | 0.989 | 0.914 | 0.991 |
| 0.61 | 0.828 | 0.973 | 0.778 | 0.933 | 0.810 | 0.979 | 0.903 | 0.983 |
| 0.6 | 0.822 | 0.973 | 0.778 | 0.933 | 0.803 | 0.979 | 0.904 | 0.984 |
| 0.59 | 0.818 | 0.973 | 0.778 | 0.933 | 0.798 | 0.979 | 0.898 | 0.984 |
| 0.58 | 0.824 | 0.974 | 0.789 | 0.938 | 0.805 | 0.980 | 0.901 | 0.984 |
| 0.57 | 0.814 | 0.975 | 0.750 | 0.938 | 0.800 | 0.980 | 0.903 | 0.985 |
| 0.56 | 0.803 | 0.975 | 0.762 | 0.941 | 0.786 | 0.981 | 0.901 | 0.985 |
| 0.55 | 0.795 | 0.976 | 0.727 | 0.941 | 0.784 | 0.981 | 0.904 | 0.986 |
| 0.54 | 0.785 | 0.976 | 0.696 | 0.941 | 0.778 | 0.981 | 0.905 | 0.986 |
| 0.53 | 0.772 | 0.976 | 0.667 | 0.941 | 0.768 | 0.981 | 0.901 | 0.986 |
| 0.52 | 0.762 | 0.976 | 0.667 | 0.941 | 0.757 | 0.981 | 0.896 | 0.986 |
| 0.51 | 0.753 | 0.976 | 0.640 | 0.941 | 0.752 | 0.981 | 0.892 | 0.986 |
| 0.5 | 0.753 | 0.976 | 0.640 | 0.941 | 0.752 | 0.981 | 0.892 | 0.986 |
| 0.49 | 0.753 | 0.976 | 0.640 | 0.941 | 0.752 | 0.981 | 0.892 | 0.986 |
| 0.48 | 0.754 | 0.977 | 0.654 | 0.944 | 0.752 | 0.982 | 0.889 | 0.987 |
| 0.47 | 0.753 | 0.977 | 0.654 | 0.944 | 0.750 | 0.982 | 0.891 | 0.987 |
| 0.46 | 0.754 | 0.977 | 0.654 | 0.944 | 0.752 | 0.982 | 0.891 | 0.987 |
| 0.45 | 0.742 | 0.977 | 0.654 | 0.944 | 0.737 | 0.982 | 0.882 | 0.987 |
| 0.44 | 0.737 | 0.977 | 0.630 | 0.944 | 0.737 | 0.982 | 0.883 | 0.987 |
| 0.43 | 0.733 | 0.977 | 0.630 | 0.944 | 0.732 | 0.982 | 0.883 | 0.987 |
| 0.42 | 0.732 | 0.978 | 0.643 | 0.947 | 0.729 | 0.983 | 0.885 | 0.988 |
| 0.41 | 0.726 | 0.978 | 0.600 | 0.947 | 0.731 | 0.983 | 0.887 | 0.988 |
| 0.4 | 0.726 | 0.978 | 0.600 | 0.947 | 0.731 | 0.983 | 0.887 | 0.988 |
| 0.39 | 0.718 | 0.978 | 0.600 | 0.947 | 0.722 | 0.983 | 0.883 | 0.988 |
| 0.38 | 0.711 | 0.978 | 0.563 | 0.947 | 0.722 | 0.983 | 0.884 | 0.988 |
| 0.37 | 0.708 | 0.978 | - | - | 0.719 | 0.983 | 0.880 | 0.988 |
| 0.36 | 0.705 | 0.978 | - | - | 0.720 | 0.983 | 0.881 | 0.988 |
| 0.35 | 0.706 | 0.978 | - | - | 0.716 | 0.983 | 0.881 | 0.988 |
| 0.34 | 0.699 | 0.978 | - | - | 0.707 | 0.983 | 0.883 | 0.988 |
| 0.33 | 0.699 | 0.978 | - | - | 0.707 | 0.983 | 0.883 | 0.988 |
| 0.32 | 0.695 | 0.978 | - | - | 0.703 | 0.983 | 0.883 | 0.989 |

Abbreviations: FFPE, formalin-fixed paraffin-embedded.
